# GNNenrich: a novel method for pathway enrichment analysis based on graph neural network

**DOI:** 10.1093/bioinformatics/btaf478

**Published:** 2025-09-08

**Authors:** Mallek Mziou-Sallami, Pierrick Roger, Arnaud Gloaguen, Claire Dandine-Roulland, Thierry Jiogho Ngaho, Solène Brohard, Kévin Muret, Florian Sandron, Eric Bonnet, Jean-Francois Deleuze, Edith Le Floch, Vincent Meyer

**Affiliations:** Centre National de Recherche en Génomique Humaine, Institut François Jacob CEA Université Paris-Saclay, Évry-Courcouronnes 91000, France; Centre National de Recherche en Génomique Humaine, Institut François Jacob CEA Université Paris-Saclay, Évry-Courcouronnes 91000, France; Centre National de Recherche en Génomique Humaine, Institut François Jacob CEA Université Paris-Saclay, Évry-Courcouronnes 91000, France; Centre National de Recherche en Génomique Humaine, Institut François Jacob CEA Université Paris-Saclay, Évry-Courcouronnes 91000, France; Centre National de Recherche en Génomique Humaine, Institut François Jacob CEA Université Paris-Saclay, Évry-Courcouronnes 91000, France; Centre National de Recherche en Génomique Humaine, Institut François Jacob CEA Université Paris-Saclay, Évry-Courcouronnes 91000, France; Centre National de Recherche en Génomique Humaine, Institut François Jacob CEA Université Paris-Saclay, Évry-Courcouronnes 91000, France; Centre National de Recherche en Génomique Humaine, Institut François Jacob CEA Université Paris-Saclay, Évry-Courcouronnes 91000, France; Centre National de Recherche en Génomique Humaine, Institut François Jacob CEA Université Paris-Saclay, Évry-Courcouronnes 91000, France; Centre National de Recherche en Génomique Humaine, Institut François Jacob CEA Université Paris-Saclay, Évry-Courcouronnes 91000, France; Centre National de Recherche en Génomique Humaine, Institut François Jacob CEA Université Paris-Saclay, Évry-Courcouronnes 91000, France; Centre National de Recherche en Génomique Humaine, Institut François Jacob CEA Université Paris-Saclay, Évry-Courcouronnes 91000, France

## Abstract

**Motivation:**

Graph neural network (GNN) models have emerged in many fields and notably for biological networks constituted by genes or proteins and their interactions. The majority of enrichment study methods apply over-representation analysis and gene/protein set scores according to the existing overlap between pathways. Such methods neglect knowledges coming from the interactions between the gene/protein sets. Here, we introduce a novel GNN-based enrichment analysis method called GNNenrich. GNNenrich, through multiple levels of embedding that integrate protein sequence properties and interactions network, establishes functional relationship to support biological interpretation.

**Results:**

GNNenrich have been tested and compared to over-representation analysis technique (g:Profiler) and graph-based method (EnrichNet). It demonstrates the capacity to reproduce results provided by others approaches and offers new perspectives for interpretation, returning relevant results supported by protein–protein interactions (PPIs).

**Availability and implementation:**

Source code is available at https://gitlab.com/cnrgh/gnn-enrich/gnn-enrich-article-demo.

## 1 Introduction

Pathway enrichment analyses have become necessary methods to provide valuable information about the biological function underlying a list of genes or proteins and their related networks of interactions (Geistlinger *et al.* 2021).

**Figure 1.**
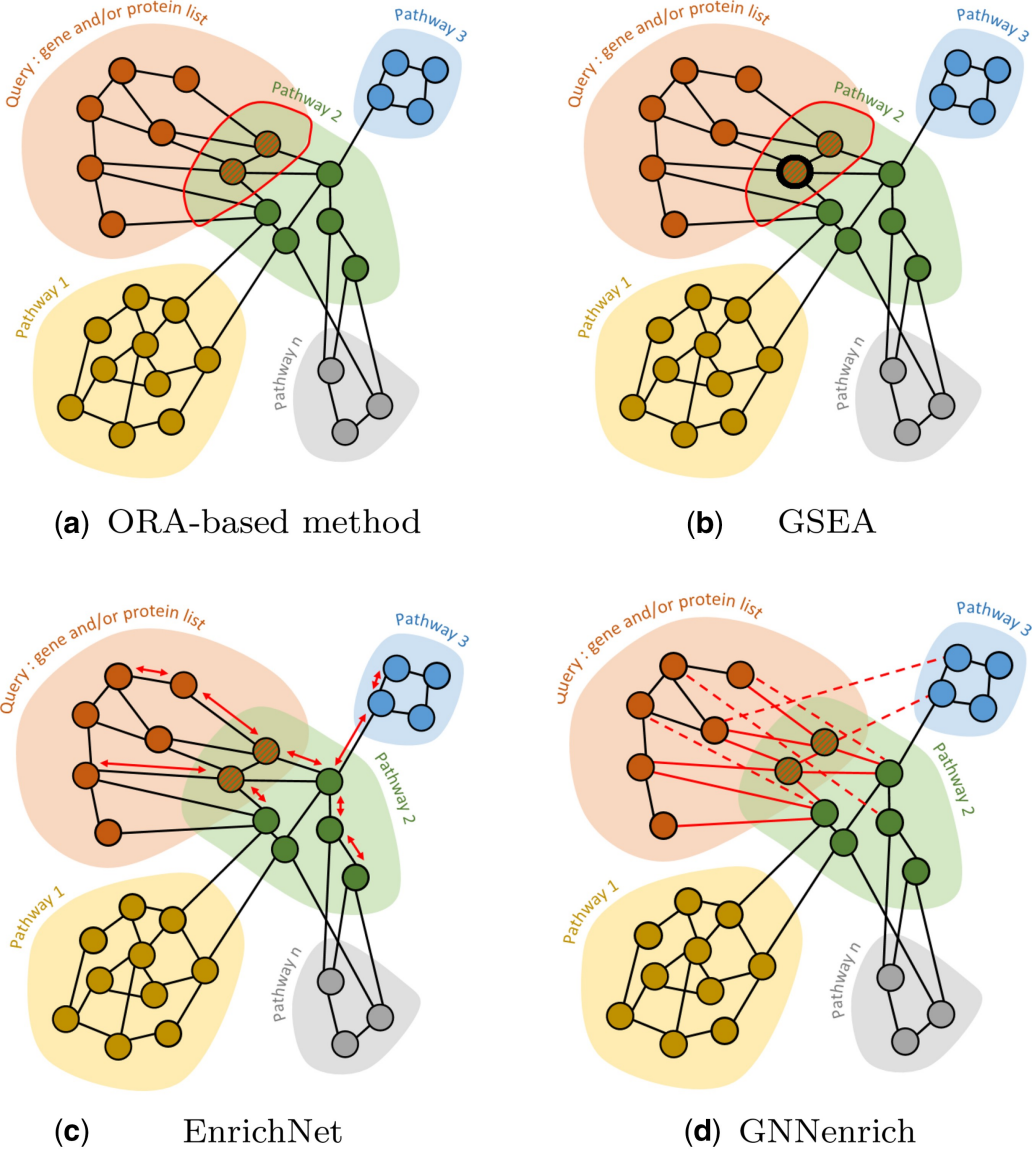
GNNenrich in enrichment methods overviews. (a) ORA-based methods focus on the overlapping genes. (b) GSEA methods focus on shared genes in the overlap and score them according to the phenotype correlation. (c) EnrichNet calculates a distance between graphs. (d) GNNenrich computes a correlation score between the encoding of the two graphs (pathways). The encircled nodes shared across pathways represent the proteins involved in the overlap, thick black circle around node the use of node weighting, the reddouble arrows the use of graph distance, the red dotted links the use of potential identified interactions by strong protein embedding correlation.

Enrichment methods can currently be classified into three main categories: over-representation analysis (ORA) techniques, gene set enrichment analysis (GSEA) methods, and modular enrichment analysis (MEA) (Huang *et al.* 2009). ORA methods, especially represented by g:Profiler (Reimand *et al.* 2007), are based on statistical tests of overrepresentation (Fig. 1a) between a query (genes or proteins of interest) and gene sets related to functional annotations. Such approaches disregard the network topology, and the selected genes are evenly weighted. GSEA methods (Fig. 1b), pioneered by Subramanian *et al.* (2005), introduce a weighting scheme based on gene expression derived from experimental data.

Despite this difference, the algorithms used in the GSEA and ORA techniques remain closely similar (Huang *et al.* 2009). As an alternative, MEA methods have been explored. Through different strategies, those approaches assign to genes weights estimated from pathway’s topology. Among the first developed methods, GANPA (Fang *et al.* 2012) used hypergeometric model to define a functional “importance” for gene weighting and CePa (Gu and Wang 2013) based its weighting methods on gene centrality measurement within pathways. While these approaches incorporate network features that the enrichment score should rely on, variation also appears for scoring methods computation depending on statistical tests based on existing overlaps or complementary measurement. Other methods such as Enrichnet (Glaab *et al.* 2012) scores the association between pathways and a list of genes/proteins using a random walk procedure to measure distances (Fig. 1c). However, proper description of interactions remains available for a limited number of proteins. For this reason, current works try to predict unknown interactions to improve the description of genes or proteins interactions.

Several machine learning-based techniques have been proposed to predict protein–protein interactions. Deep learning methods have been introduced with the use of the structure of amino acid sequences as features in order to vectorize a protein. These works typically use deep neural network (DNN) (Du *et al.* 2017), autoencoder (Sun *et al.* 2017), convolutional neural network (CNN) (Hashemifar *et al.* 2018), Siamese residual CNN (RCNN) (Chen *et al.* 2019) or recurrent neural networks (RNN) (Li *et al.* 2018) to extract protein features from the amino acid sequence.

An evolution of these approaches suggested by Dutta and Saha (2020) and Nambiar *et al.* (2023) integrates protein structure information through several transformer based architecture for protein encoding and protein–protein (PP) interaction.

Recently, graph convolutional networks (GCN) emerged as reliable models designed to process graph-structured data. The GCN framework have also been developed to learn a representation of nodes based on the protein primary structure sequences (Liu *et al.* 2019). The resulting representations of the proteins are used to predict interactions. The topological information provided by the databases referencing protein–protein interactions are progressively used to improve models (Zhang *et al.* 2021).

Lastly, improvement suggested the GNN-PPI model (Lv *et al.* 2021) predicts protein encoding followed by an aggregation step adding graph convolution. This graph-based methods have shown stronger performance for predicting interactions between proteins in comparison with methods relying only on sequence information.

In this paper, we propose GNNenrich: a new method for identifying functional annotations through enrichment analysis based on sequence properties and related protein–protein interactions (Fig. 1d). GNNenrich is a deep learning-based model capable of performing enrichment analysis across multiple types of annotations.

**Figure 2.**
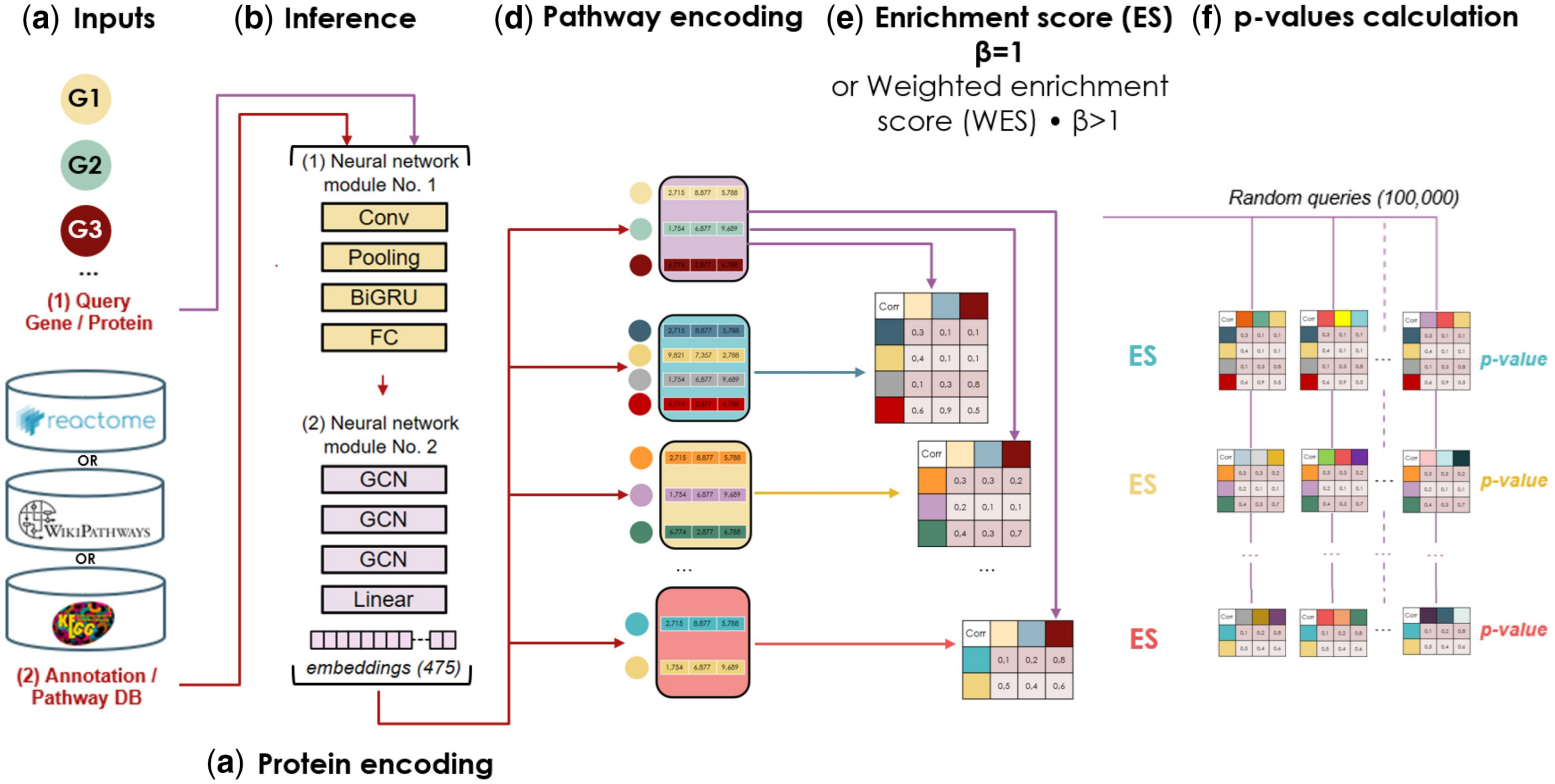
Method overview: (a) GNNenrich requires as input (1) a list of genes or proteins, and (2) a collection of gene sets corresponding to biological pathways and processes. (b) Inference of GNN model to get the proteins encoding of query and pathways. (c) Protein encoding prediction. (d) Using proteins encoding, pathways encoding are obtained. (e) The enrichment score is derived from the correlation between pathway and query encodings. (f) Statistical significance assessment.

The GNNenrich framework is constituted by four components. First, an adaptation of the GNN-PPI model in charge of single protein encoding (Lv *et al.* 2021) is used to then determine a GNN encoding at the pathway level. After that, the comparison between each encoded pathway belonging to a chosen database and an encoded query is formalized by a weighted correlation score emphasizing the contribution of overlaps and/or interactions. Finally, to assess significance of the results, *P*-values are determined for each score obtained against functional gene sets referenced in the database (Fig. 2). Here, GNNenrich introduces a novel approach for integrating PPI data into enrichment analysis. GNNenrich has been evaluated against the ORA technique [g:Profiler (Reimand *et al.* 2007)] and the graph-based method (EnrichNet). It showcases its ability to replicate findings from these approaches while introducing fresh interpretative insights.

## 2 Materials and methods

### 2.1 Training data and experimental setting

The STRING database (Szklarczyk *et al.* 2019) integrates the largest sources of protein-protein interaction information. We selected the *Homo sapiens* data in version 11 including 19 566 proteins. Protein average length is 571.69 and varies between 21 and 35 991 amino acids. These proteins are related to 3 470 906 interactions involving activation, binding, catalysis, expression, inhibition, post-translational modification, and reaction. We divide the interactions set into training set (80%) and test set (20%). The training set involves 16 073 proteins included all types of interactions. All protein interactions referenced for the *Homo sapiens* organism included the protein complete sequences have been used for training (Section 4, available as supplementary data at *Bioinformatics* online) to generate protein embedding based on an adaptation of GNN-PPI (Lv *et al.* 2021) for further aggregation at the pathway level.

### 2.2 Use cases for enrichment evaluation

To evaluate GNNenrich, we used three curated canonical databases from the C2 collection (2023.2.Hs) commonly employed to evaluate enrichment analysis methods (Subramanian *et al.* 2005). Without applying any prior filtering, we used 733 pathways extracted from WikiPathways, 186 pathways from Kyoto Encyclopedia of Genes and Genomes (KEGG) and 1692 pathways from Reactome. To assess our results, we chose to focus our analysis on two use cases previously described in two distinct pathologies: Parkinson’s (PD) disease, which involves 126 genes and 635 PPIs and gastric cancer (GC) with 100 genes and 1829 PPIs (Section 1, available as supplementary data at *Bioinformatics* online). These two queries (Section 14, available as supplementary data at *Bioinformatics* online) share only two genes (*IL1RN* and *IL1B*). From a biological point of view, cancer and Parkinson’s disease differ fundamentally in their causes, symptoms, and treatments.

### 2.3 Method overview

#### 2.3.1 Protein encoding using graph neural network

On the basis of previous work through the GNN-PPI development (Lv *et al.* 2021), describing the benefits related to the integration of the amino-acid properties and sequences for PPI prediction (Section 2, available as supplementary data at *Bioinformatics* online), proteins were set as nodes, primary amino acid sequences and properties as features, and protein–protein interactions as edges *E* to construct the graph *G* and learn the model parameters *w* (Section 3, available as supplementary data at *Bioinformatics* online). The first component for protein embeddings incorporates the architecture (Fig. 2c) previously suggested by GNN-PPI (Lv *et al.* 2021). To train the model on STRING and determine the optimal size of the protein descriptor vector denoted by $h_{P}$, we conducted an automated fine-tuning operation based on Bayesian optimization. All proteins were encoded using the optimal model’s predictions (Section 5, available as supplementary data at *Bioinformatics* online).

#### 2.3.2 Pathways encoding

The second component aggregates the encoding of proteins at the pathway level (Fig. 2d). This embedding brings information about proteins as well as interactions. Pathways containing *N* proteins will be characterized by the concatenation of *N* vectors encoding their related proteins as follows.


(1) $$\begin{array}{c} h_{\mathrm{pathwa}y_{i}}=\left[\begin{array}{c} h_{P_{1}}\\ .\\ h_{P_{k}}\\ .\\ h_{P_{N}} \end{array}\right] \end{array}$$

#### 2.3.3 Enrichment score

A Pearson correlation score measures the similarity between two sets of genes/proteins based on their respective encodings (Fig. 2e). The first set ($h_{\mathrm{query}}$) corresponds to a gene/protein selection of interest, the second ($h_{\mathrm{pathwa}y_{i}}$) to a pathway within annotation database. $h_{P_{1}}$ represents the embedding of $P_{1}$ while $h_{P_{k}}$ corresponds to the embedding of the k-th protein in the query set, and $h_{P_{N}}$ to that of N-th protein. Additionally, $h_{P_{M}}$ denote the embedding of the *M*-th protein within the *i*-th pathway. We denote $h_{\mathrm{query}}$ and $h_{\mathrm{pathwa}y_{i}}$ as:


(2) $$\begin{array}{c} h_{\mathrm{query}}=\left[\begin{array}{c} h_{P_{1}}\\ .\\ h_{P_{k}}\\ .\\ h_{P_{N}} \end{array}\right]\;\text{and}\;h_{\mathrm{pathwa}y_{i}}=\left[\begin{array}{c} h_{P_{1}^{\prime }}\\ .\\ h_{P_{l}^{\prime }}\\ .\\ h_{P_{M}^{\prime }} \end{array}\right] \end{array}$$

We define the correlation matrix as follow:


(3) $$\begin{array}{c} C(\mathrm{query},\mathrm{pathway})=\left(\begin{array}{cccc} C_{h_{P_{1}},h_{P_{1}^{\prime }}} & C_{h_{P_{1}},h_{P_{2}^{\prime }}} & \cdots & C_{h_{P_{1}},h_{P_{M}^{\prime }}}\\ C_{h_{P_{2}},h_{P_{1}^{\prime }}} & C_{h_{P_{2}},h_{P_{2}^{\prime }}} & \cdots & C_{h_{P_{2}},h_{P_{M}^{\prime }}}\\ \vdots & \vdots & \ddots & \vdots \\ C_{h_{P_{N}},h_{P_{1}^{\prime }}} & C_{h_{P_{N}},h_{P_{2}^{\prime }}} & \cdots & C_{h_{P_{N}},h_{P_{M}^{\prime }}} \end{array}\right) \end{array}$$

where $C_{h_{P_{k}},h_{P_{l}^{\prime }}}$ is the Pearson correlation coefficient between $h_{P_{k}}$ and $h_{P_{l}^{\prime }}$. A filtration threshold is then applied in order to only retain genes/proteins with a score >0.9 (while setting others to zero) to exclude weak levels of interactions in the scoring process. Moreover when the correlation between a gene of the query and a gene of the pathway is equal to 1, which means that these two genes are the same, all the correlations between the other genes of the query and this gene are set to 0 in order to reduce the influence of interactions solely induced by overlapping genes. The highest score of each row is selected to associate each protein in the query with the strongest match. We define the enrichment score *ES* as $\mathrm{mean}(max_{C(\mathrm{query},\mathrm{pathway}))}$ where $max_{C}$ is calculated with the expression below:


(4) $$\begin{array}{c} max_{C_{(}q\mathrm{uery},\mathrm{pathway})}=\left[\begin{array}{c} {\text{max}\;}_{l=1..M}C_{h_{P_{1}},h_{P_{l}^{\prime }}}\\ {\text{max}\;}_{l=1..M}C_{h_{P_{2}},h_{P_{l}^{\prime }}}\\ \ldots \\ {\text{max}\;}_{l=1..M}C_{h_{P_{n}},h_{P_{l}^{\prime }}} \end{array}\right] \end{array}$$

This score, derived from the GNN model, estimates the association between the query and the pathway.

#### 2.3.4 Weighted enrichment score

Revisiting the correlation matrix presented in Equation (3), we can decompose, C(query,pathway) as a sum of two matrices $C_{\mathrm{overlap}}(\mathrm{query},\mathrm{pathway})$ and $C_{\mathrm{interaction}}(\mathrm{query},\mathrm{pathway})$, such that ∀(i,j)∈[[1;N]]×[[1;M]]:


$${\left(C_{\mathrm{overlap}}(\mathrm{query},\mathrm{pathway})\right)}_{i,j}=\{\begin{array}{cc} 1 & \text{if}\;C_{h_{P_{i}},h_{P_{j}^{\prime }}}=1\\ 0 & \text{otherwise}. \end{array}$$

$${\left(C_{\mathrm{interaction}}(\mathrm{query},\mathrm{pathway})\right)}_{i,j}=\{\begin{array}{cc} C_{h_{P_{i}},h_{P_{j}^{\prime }}} & \text{if}\;C_{h_{P_{i}},h_{P_{j}^{\prime }}}\neq 1\\ 0 & \text{otherwise}. \end{array}$$

At this stage, the integration of a weighting coefficient for $C_{\mathrm{overlap}}$ matrix allows for differential emphasis on the contribution of overlap relative to interactions in the final scoring.


(5) $$C(\mathrm{query},\mathrm{pathway})=C_{\mathrm{interaction}}(\mathrm{query},\mathrm{pathway})+\beta C_{\mathrm{overlap}}(\mathrm{query},\mathrm{pathway})$$

We investigate the evolution of the result provided by this method for β presenting integer values between [1.0.100]. Weighted enrichment score *WES* is defined as $\mathrm{WES}=\mathrm{mean}(max_{C(\mathrm{query},\mathrm{pathway}))}$ where $max_{C}$ is calculated following the Equation (4).

#### 2.3.5 Statistical significance assessment

In order to statistically assess our enrichment scores, we sampled 100 000 random lists from gene/proteins referenced in the STRING database fitting the number of the genes/proteins included in our two queries GC and PD (Fig. 2f).

For each random list obtained, a score was computed for each pathway. The empirical null distribution of the scores obtained by random sampling was used to estimate the p-value of the observed score obtained for that pathway. According to our current number of permutations, the minimal significant observed *P*-value is .00001. The *P*-values were then corrected with the Benjamini–Hochberg (BH) procedure to control for the false discovery rate (FDR). The adequacy of 100 000 random permutations to sample the null distribution, as well as the impact of random sampling on the scalability of GNNenrich, are further evaluated in Sections 21 and 22, available as supplementary data at *Bioinformatics* online, respectively.

## 3 Results

### 3.1 GNNenrich monitoring of the model’s weighting

A key motivation behind GNNenrich is the incorporation of protein-protein interactions in the enrichment analysis while also accounting for the overlap between the query list and pathways. To achieve this, we introduced the weighting β to balance the influence of interactions relative to the overlap. The impact of β is monitored through our six different scenarios including the enrichment analysis of PD and GC on the three pathways databases KEGG, Reactome, and WikiPathways aiming to understand and determine relevant β for further analysis.

The variation of the number of significant pathways related to the increase of β coefficient is analyzed and compared to significant results within pathways returned by g:Profiler (ORA-based method) and EnrichNet (graph-based method). The pathways identified by GNNenrich presenting a BH<0.05 were selected and compared against g:Profiler pathways showing a BH<0.05 and EnrichNet pathways related to threshold suggested by the regression model for the different use cases (Section 6 and Table 37, available as supplementary data at *Bioinformatics* online).

**Figure 3.**
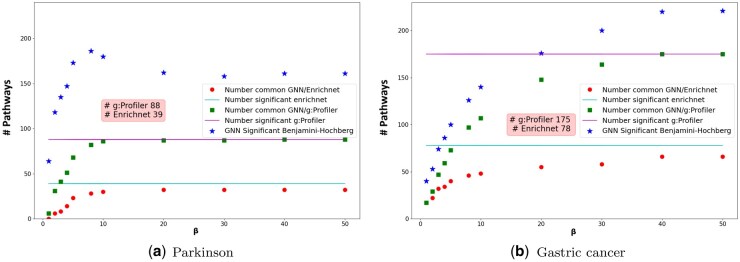
Variation of total (blue star) and shared significant pathways with g:Profiler (green square) and EnrichNet (red dot) for β∈[1,50]. Significant pathways were defined as those meeting the following criteria: for GNNenrich and g:Profiler, a Benjamini–Hochberg adjusted *P*-value (BH) <0.05; for EnrichNet, the significance thresholds were applied as specified in Table S37, available as supplementary data at *Bioinformatics* online.

In a context where the augmentation of β progressively favors pathways sharing proteins, an increase of β coefficient is broadly related to an augmentation of the number of significant pathways (Fig. 3). Inversely, for low values of β where the score of the GNN models is mainly driven by the density of interaction available, a lower number of pathways were detected. This rise of the number of significant pathways generally related to the augmentation of the β coefficient tends to stabilize within the range of 20 to 40 with no evolution up to β=100 (Fig. 3, available as supplementary data at *Bioinformatics* online). A more detailed description shows for the WikiPathways database, in the Parkinson’s case (Fig. 3a), 64 significant pathways are detected for β=1. This number rises and achieves 162 pathways when β=20. For the GC (Fig. 3b), 40 significant pathways are detected for β=1. This number reached 220 for β=40. The observed differences for the different use cases respectively could be explained by the PPIs density between GC and PD (1829 versus 635) which influences the model’s behavior. Additionally, the different numbers of PPIs distinctly referenced in the three databases participated as well by their design to the impact on the model conducts. In this way, the results from the WikiPathways and Reactome larger databases follow a similar pattern in comparison to KEGG. With fewer pathways, KEGG shows a smaller or negligible impact related to β in PD and GC (Fig. 3, available as supplementary data at *Bioinformatics* online). According to our range of studied β, an evolution of the number of similar pathways shared between GNNenrich, g:Profiler and EnrichNet can be observed. The increase in β is systematically associated with an increase in the number of similar pathways detected by the three methods (Fig. 5, available as supplementary data at *Bioinformatics* online). These results highlight that assigning greater weight to overlapping genes between the query and pathway sets leads to increased concordance among GNNenrich, g:Profiler, and EnrichNet results. In contrast, lower values of β enable the identification of pathways uniquely detected by GNNenrich, which exhibit lower gene overlap but higher interaction densities. This behavior, driven by interaction-specific features and the underlying GNN architecture, suggests promising avenues for further exploration of interaction-based enrichment strategies.

**Figure 4.**
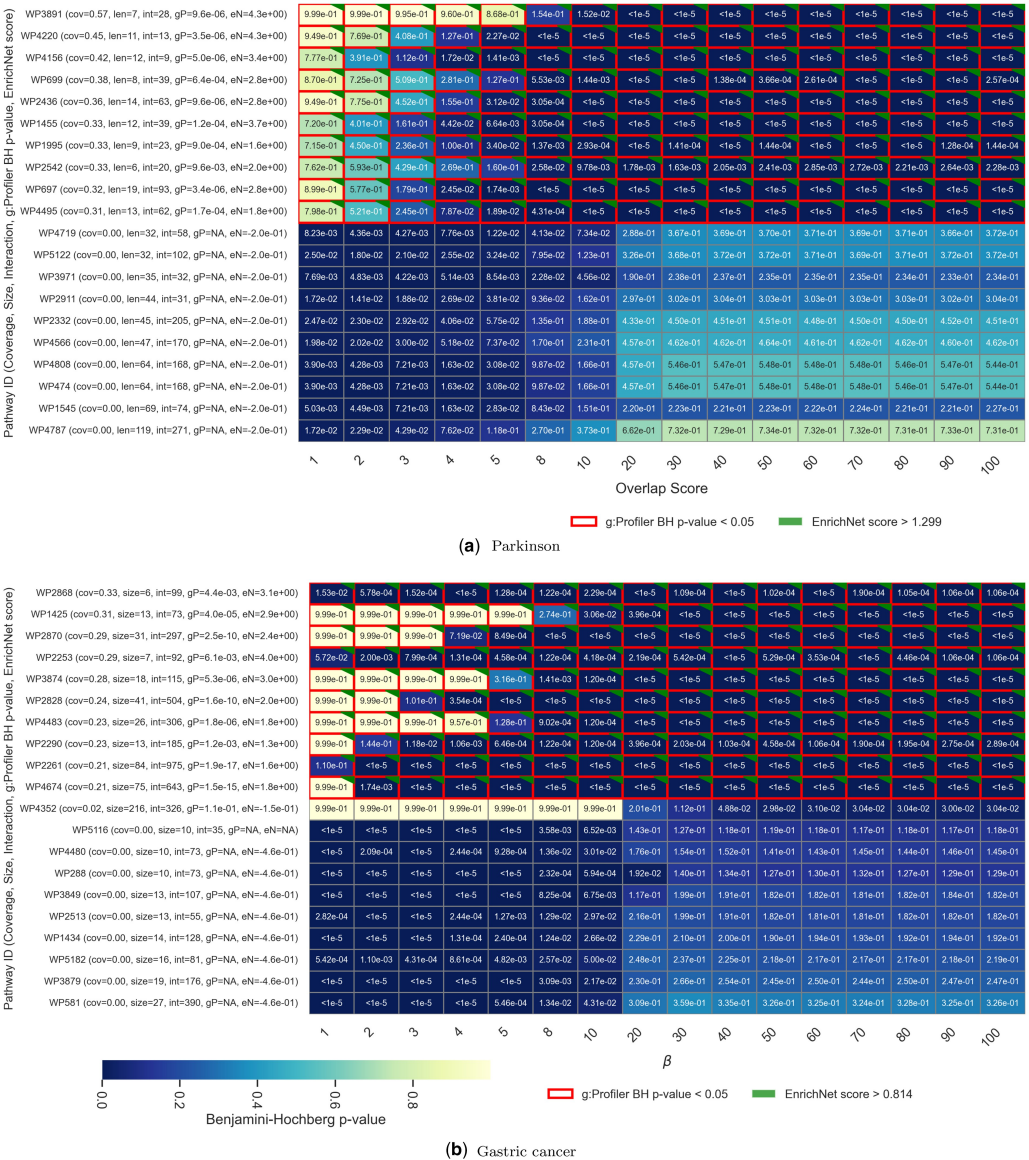
Selection of the 10 top and bottom lines of the heatmaps illustrating the evolution pathway detected within WikiPathways database and their corrected *P*-value (BH) for β∈[1,100]. The *x*-axis referred to β variation. The *y*-axis lists the pathways, ordered according to their coverage level. For each pathway, the following metrics are indicated: coverage = overlap/pathway size, pathway size (number of genes), number of interactions, g:Profiler BH-adjusted p-value (gP), and EnrichNet score (eN). Darker color shades indicate more significant p-values. Cells outlined in red indicate g:Profiler significant pathways. Green-cornered cells highlight pathways identified by EnrichNet. Each cell contain the GNNenrich adjusted *P*-values.

To further analyze the behavior of GNNenrich, we conducted a detailed investigation into how pathway significance evolves across varying values of β. To this end, we generated heatmaps illustrating the pathways enriched from the PD and GC gene lists, respectively, displaying pathways significantly found in at least one of the β value conditions (Fig 4a and b). This global dynamic overview enables the monitoring of fluctuations in pathway detection as a function of variations in β, effectively capturing the transition from interaction-driven to overlap-driven identification.

Here, two main trends emerge. On the one hand, higher values of β emphasize pathways primarily identified through gene/protein overlap between the query and pathways. These pathways typically exhibit higher coverage of their associated genes/proteins and appear in darker tones in the upper right region of the figures. On the other hand, the lower-left region captures pathways that are mainly detected at lower β values, where the method prioritizes protein–protein interactions to determine enrichment.

The pathways jointly identified by GNNenrich and g:Profiler tend to exhibit high coverage by the gene’s query. In contrast, pathways identified solely by GNNenrich often correspond to cases with low or even zero direct overlap, relying instead on connections in the networks described in the STRING database. This category of pathways often loses significance as β increases, reflecting their sensitivity to stricter overlap constraints. Similar trends were observed across KEGG and Reactome (Figs 29–32, available as supplementary data at *Bioinformatics* online).

These observations can be formalized by the determination of the correlation between BH *P*-values and both the number of intersections and the number of interactions for an increasing range of the β value (Fig. 4, available as supplementary data at *Bioinformatics* online). The results confirm that EnrichNet and g:Profiler appear to rely more heavily on gene overlap, whereas GNNenrich, through its parameterization system, integrate both interactions and overlap with their relative importance determined by the weighting applied to β. These observations highlight the complementary nature of GNNenrich under lower β conditions, where network information is leveraged to reveal potentially relevant biological pathways even in the absence of direct gene overlap. As illustrated, these two strategies—overlap-driven and interaction-driven enrichment—can coexist, offering a broader and complementary perspective for pathway analysis. Based on these initial analyses, we decided to further investigate the results in greater detail by focusing on two β conditions, 10 and 40, which represent two distinct scenarios in which the results produced by GNNenrich are driven by both interactions and overlap or mainly by overlap, respectively.

### 3.2 Agreement between g:Profiler, EnrichNet, and GNNenrich

**Figure 5.**
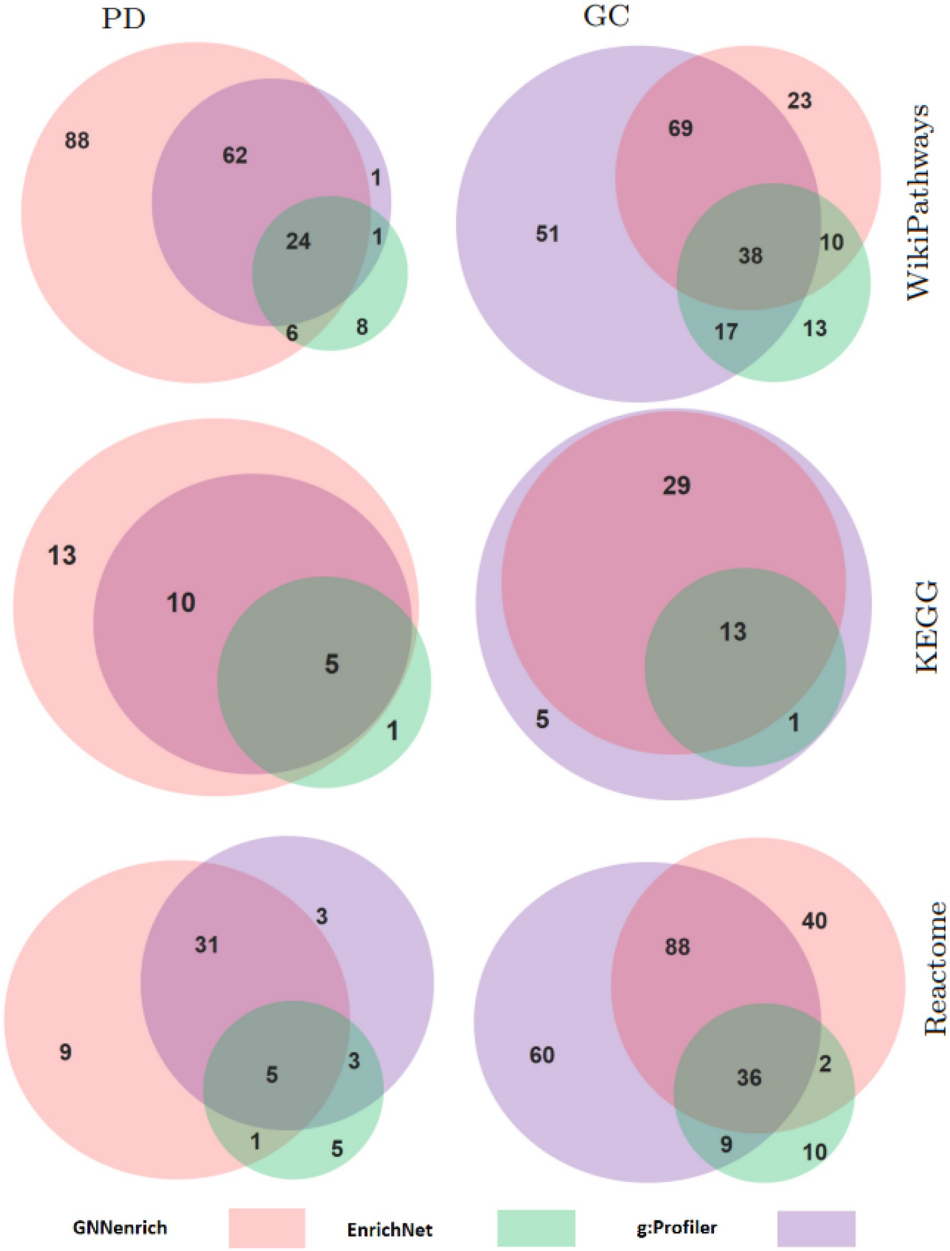
Common pathways between GNNenrich, EnrichNet, and g:Profiler. Overlap weighting coefficient has been set to β=10. Details on statistical significance can be found in Table 40 and Section 20, available as supplementary data at *Bioinformatics* online.

To complete the exploration of the results provided by GNNenrich, the concordance between g:Profiler, EnrichNet, and GNNenrich was investigated. To examine the input of the interaction contribution provided by GNNenrich, a β value of 10 was applied to the model-generated score (Fig. 5). This threshold may be seen as a weighting trade-off that allows the simultaneous consideration of both intersection and interaction, including, notably, detection entirely based on interaction (Table 35, available as supplementary data at *Bioinformatics* online).

The KEGG database shows a higher overlap ratio for both pathologies. In the PD query, a total of 15 common pathways were identified, completely including the pathways identified by g:Profiler and 5 among 6 of those from EnrichNet. Similarly, the GC pathways found by GNNenrich on KEGG are common with g:Profiler and represent 92.85% (13 among 14) of the pathways identified by EnrichNet.

The research of enrichment for Parkinson’s disease in the WikiPathways database reveals a total of 92 pathways common with either g:Profiler or EnrichNet. These pathways include 97.2% of the pathways identified by g:Profiler and 76.92% of those of EnrichNet.

Against the Reactome database, 37 common pathways are shared with at least one of the two methods, representing (36 of 42) 85.71% of the pathways identified by g:Profiler and 42.85% (6 among 14) of EnrichNet results. The GC is related to lower overlap ratios across methods. Query on WikiPathways using GNNenrich identified 107 common pathways with g:Profiler and 48 with EnrichNet. The Reactome database point out similar results with 126 common pathways found, with 124 among 193 shared with g:Profiler and 38 among 57 shared with EnrichNet.

Additionally, we investigated the specific result gathering pathways detected only by g:Profiler and EnrichNet. In PD use case and WikiPathways, one pathway can be observed and three can be referenced in Reactome. These four pathways exhibit varying degrees of gene overlap, ranging from two to three genes and interactions with the Parkinson’s list ranging from 0 to 20.

At this studied level of β, these discrepancies may be associated with the stronger dependency in the scoring of g:Profiler and EnrichNet on proteins overlapping between the query and pathways, combined with a limited number of interactions. Nonetheless, an exploration of the results provided by higher β favorizing the contribution of the overlap lead to a significant *P*-values related to these pathways becoming shared by the three methods (Fig. 5, available as supplementary data at *Bioinformatics* online).

Similar trends are observed in the GC use case. In both cases, the overlap assessment conducted using the SuperExactTest yields statistically significant *P*-values, supporting the conclusion that the observed overlaps are unlikely due to chance (Section 20, available as supplementary data at *Bioinformatics* online).

Finally, the GNNenrich specific pathways were investigated through our different use cases. These analyses associated to PD lead to 88 pathways on WikiPathways, 13 on KEGG and 9 on Reactome. For the GC, 23 pathways on WikiPathways and 40 against Reactome are detected. No specific pathways were identified by GNNenrich in KEGG. Within the specific results produced by GNNenrich, two categories are emerging.

The first category of results reveals low ratios between the number of overlapping genes and the size of a pathway. For the PD, we can observe 78 pathways on WikiPathways, 13 on KEGG and 7 on Reactome fitting this pattern. Our GC study shows similar properties with 14 on WikiPathways and 30 on Reactome.

For example, in WikiPathways, the pathway “Ultraconserved region 339 modulation of tumor suppressor microRNAs in cancer” and “WNT signaling” with respectively one gene and four genes in the overlap can be pointed out to illustrate this category. In Reactome, the pathways “Reactome FCERI mediated MAPK activation” with 87 genes among which the three following genes in the overlap KRAS, HRAS and NRAS and “Reactome G2/M DNA damage checkpoint” which includes 94 genes, with TP53 and ATM being part of the overlapping integrate as well the same class of results.

The second category is relative to pathways exhibiting a high number of interactions and complete lack of genes in overlap between the queries and pathways. This scenario includes 10 pathways on WikiPathways, and 2 on Reactome inside the PD use case and 9 pathways on WikiPathways and 10 on Reactome for the GC.

In this context, in a more detailed way, the following pathways described in the Reactome database can be pointed up: “Reactome erythropoietin activates phospholipase C gamma PLCG” including 7 genes associated to 97 PPIs and “Reactome IRAK2 mediated activation of TAK1 complex” with 10 genes and 125 PPIs. In WikiPathways, the “4hydroxytamoxifen dexamethasone and retinoic acids regulation of P27 expression” (18 genes and 176 interactions) or “EPO receptor signaling” (26 genes and 390 interactions) present a similar profile.

These divergent results in comparison with g:Profiler and EnrichNet can be supported by the design behind each method. In this specific situation, g:Profiler may not dispose from available material to determine significant *P*-value related to Fisher test requiring a minimal amount of overlapped genes between queries and referenced pathways. Moreover, it should be noted that universe of genes used by g:Profiler in its tests, restricted to the genes annotated in the pathways, may also have a little influence of the differences observed with GNNenrich’s results. On the other hand, EnrichNet may benefit from the topological information brought by the interaction network to explore larger range of pathways profile. Nonetheless, EnrichNet shows globally a lower number of pathways returned in our use cases. This behavior may come from the linear model currently applied by the method to define a scoring threshold. The difference in topological information processing between GNNenrich and EnrichNet may lead as well to the distinction of specific pathways.

The results presented here suggest that GNNenrich, placed in a trade-off context between the contribution of the interactions and overlaps, provide results including for the most part the results provided by ORA-based and topological methods. Additionally, GNNenrich provides a complementary perspective by incorporating interaction map densities and protein properties.

### 3.3 Functional enrichment analysis

To evaluate GNNenrich, the relevance of the results were detailed within the two studied uses cases. According to our previous observations, a weighting coefficient β was set to a value of 10 representing a trade-off between the contribution from overlaps and interactions.

First, a focus on pathways detected by GNNenrich showing the lowest *P*-value (*P* < 10^−5^) and shared with WikiPathways (41 pathways) (Table 3, available as supplementary data at *Bioinformatics* online), KEGG (10 pathways) (Table 9, available as supplementary data at *Bioinformatics* online) and Reactome (18 pathways) (Table 14, available as supplementary data at *Bioinformatics* online) was performed. Inside this selection, the most prominent pathways related to Parkinson’s disease were investigated (Table 3, available as supplementary data at *Bioinformatics* online). The “Parkinson’s Disease Pathway” (WP2371) from WikiPathways and the “KEGG Parkinson’s Disease” associated to the lowest *P*-values (*P* < 10^−5^) are part of the pathways shared by the three methods. Closely related pathways to Parkinson’s disease are also observed, notably illustrated by the susceptibility to SIDS (sudden infant death syndrome), which is mediated by the activities of dopaminergic neurons (Lavezzi 2021). The “Monoamine GPCRS” pathway (WP58) is as well significantly detected by the three methods. G protein-coupled receptors are currently highly studied therapeutic targets for treating Parkinson’s disease symptoms (Jones-Tabah 2023).

Additionally, the pathways linked to dopaminergic neurons “Dopaminergic neurogenesis” (WP2855), “Nicotine Dopaminergic Activity” (WP1602), “Dopamine metabolism” (WP2436), and “Reactome Dopamine Receptor” remain, respectively, significant. Our attention on the common pathways with g:Profiler denotes the presence of the “Circadian rhythm genes” (WP3594) pathway, which can be easily associated with the “sleep regulation” pathway (WP3591) related to the mentioned sleep disturbance symptoms observed in people affected by PD (Li *et al.* 2017).

Another category of pathways related to neuroinflammation was identified. The “Neuroinflammation and Glutamatergic Signaling” pathway (WP5083), with 8 common genes out of 140 and 437 total PPIs, indicating a strong connection to the pathology. Additionally, the “IL10 Anti-inflammatory Signaling Pathway” (WP4495) was significantly found with 4 common genes out of 12 and 62 PPIs. Related functional pathways within KEGG (Table 9, available as supplementary data at *Bioinformatics* online) and Reactome databases (Table 14, available as supplementary data at *Bioinformatics* online) can also be observed.

The results described from Reactome show notably functional reaction pathway related to functionalization of compounds and glutathione conjugation closely associated to PD (Bjørklund *et al.* 2021). On the other side, the results obtained against the KEGG database provide as well related metabolic pathway represented by Glutathione metabolism with largely depicted role in PD (Asanuma and Miyazaki 2021). On the whole, the significant pathways returned by GNNenrich and shared with one or both of the methods seem to be globally relevant and related to PD.

In GC, we noticed a larger number of pathways returned with a *P*-value (*P* < 10^−5^). Most of these pathways are related to cancer pathways (Table 20, available as supplementary data at *Bioinformatics* online). Among them are, “endometrial cancer”, “bladder cancer”, “glioblastoma signaling pathways”, and “breast cancer pathway”. Through KEGG database (Table 26, available as supplementary data at *Bioinformatics* online), the top significant results include “acute myeloid leukemia”, “bladder cancer”, “chronic myeloid leukemia”, “colorectal cancer”, and “endometrial cancer”. The results observed from Reactome (Table 30, available as supplementary data at *Bioinformatics* online) complete this landscape notably with the pathway “Reactome Gastrin CREB signaling pathway via PKC and MAPK” (Sapio *et al.* 2020). The highest enrichment results found through the three databases also encompass a large number of signaling pathways associated with cell growth and proliferation, such as EGFR, ERBB, MAPK, PI3K-Akt, and Ras considered as main actors in cancer context (De Luca *et al.* 2012).

In a second step of assessment, the results returned only by GNNenrich were investigated. For Parkinson’s disease, the “GPCRS other” (WP117) (*BH* = 0.023) and “GPCRS Class A Rhodopsinlike” (WP455) with *BH* = 0.004 linked to the therapeutic target GPCRs were detected.

GNNenrich specific results include also several pathways within the three databases associated with the regulation of neuronal processes and various neurological conditions such as: “brain-derived neurotrophic factor (BDNF) signaling pathway” (Murer *et al.* 2001), “serotonin and anxiety” (Joling *et al.* 2018) and “neural crest differentiation” (Yang *et al.* 2017) (more details in Tables 6, 11, and 17, available as supplementary data at *Bioinformatics* online). These results can be mostly categorized as pathways sharing a low rate of overlap nonetheless supported by a large number of PPIs enabling the enrichment detection. On the same principles for GC several pathways linked to inflammatory, cell proliferation and tumorigenesis are specifically identified by GNNenrich. As example in WikiPathways, we can cite “WNT Signaling” (Zhan *et al.* 2017) and “TNF Related Weak Inducer of Apoptosis (TWEAK) Signaling Pathway” (Cherry *et al.* 2015). More details are provided in Tables 23 and 33, available as supplementary data at *Bioinformatics* online.

Finally, GNNenrich was evaluated for its ability to generate meaningful overlap-free results based solely on protein–protein interactions (PPIs). To further support this finding, we extended the analysis using the MalaCards database (Rappaport *et al.* 2017), which integrates comprehensive information on disease–gene associations. In both the Parkinson’s disease (PD) and gastric cancer (GC) use cases, the overlap-free results show a notable correspondence with genes and proteins associated with these diseases, as documented in MalaCards (Section 23, available as supplementary data at *Bioinformatics* online). We can mention, in PD and WikiPathway database “The caloric restriction and aging pathway” notably identified (BH = 4.75E-02) by five significant genes recognized for its impact on aging and neurodegenerative diseases by modulating processes like inflammation and oxidative stress (de Carvalho 2022). In GC, the presence of the altered glycosylation of MUC1 in tumor microenvironment pathway (WP4480) (BH = 2.82E-04), linked to the tumor microenvironment is clearly related to the pathology (Hinshaw and Shevde 2019) with 7 genes and the EPO receptor signaling pathway (WP581) (BH = 2.82E-04), with 14 genes. Figure 2, available as supplementary data at *Bioinformatics* online illustrates as well the links between our GC query and the two pathways. In a whole, these results only based on interaction information suggest a relevant contribution of the PPIs for the enrichment detection integrated in GNN models embedding and correlation computation.

## 4 Discussion

Nowadays, most common approaches for functional analysis are still relying on statistical test based on shared genes distribution between queries and functionally annotated gene sets. Nonetheless, the increasing knowledge and available data regarding functional network, protein structure and interactions are accompanied by a fast pace evolving of deep learning methods offer. Through this dynamic, the purpose of the work presented here was dedicated to demonstrate the interest of GNN related methods for enrichment analysis. To this end, the innovation lying in this work were the development of GNN models generating scoring methods based on protein sequence properties and interactions supported by weighting mechanisms to explore ranges of scenarios between shared genes observation and interaction densities analysis. However, the principles behind the current architecture of the methods enable the possibility to include new panels of embedding strategies to investigate further the benefit of alternative architecture like adaptation of heterogeneous GNN (Zhang *et al.* 2019) or other complementary deep learning methods transformer-based (Sun *et al.* 2025) and related optimizations.

Through this study, we suggest that enrichment analysis could be improved by integrating both PPIs and intersection analysis in GNN-based model. While ORA may remain for the time being the most used approach in the wait of accurate and exhaustive information regarding protein–protein interaction, this work supports that GNN with the right adjustments are able to produce close results to current approaches and provide new strategies and complementary angles for enrichment analysis.

Related to the use of PPIs, limitations may be expected from the availability and quality of the sources for interaction description. Nonetheless, the constant improvement of the knowledge related to protein interaction and functional networks will support the improvement of GNN-based model for protein interaction and functional analysis. Moreover, the current foundation of this method demonstrates a fair degree of resilience to information loss during the learning phase (Section 18, available as supplementary data at *Bioinformatics* online). Complementing this, the configurable level of correlation applied during inference and interaction selection potentially enables the model to focus on the strongest signals, thereby limiting the noise introduced by weaker interactions in the enrichment determination process. Proteins acting as hub found in numerous functional pathways and associated to a high level of interaction may also raise concerns regarding their impact in GNNenrich results. Nevertheless, our complementary analysis of the hub proteins proportion identified in the GNNenrich results does not reveal major biases related to the shared and specific pathways detected nor does it indicate an over-representation of high-degree hubs previously observed in the complete set of pathway databases used in this study (Section 19, available as supplementary data at *Bioinformatics* online).

Advantages associated to deep learning methods are notably due to the feasibility of including additional sources/features of data within the model. In addition, in a complementary manner to the protein–protein interaction information, experimental data originating from gene expression analysis or mutational burden scoring (Li and Leal 2008) may improve the gene targeted by the models and the biological relevance associated to the results. Besides, these results highlight the interest in having access to a parameterization of the model, which will provide flexible results fitting the constraints of the use case and availability of related gene/protein interaction data.

As a conclusion of our investigation through distinct use cases showcasing a variety of situations depending on the level of interaction and annotation available between queries and databases, our recommendation for a relevant usage of the method would be to start the analysis with higher values of β that favor overlap and provide closer results to the current popular method (ORA-based) to then progressively decrease β incorporating results based on interaction densities as new insights for enrichment analysis.

## 5 Conclusion

Here, we presented GNNenrich, a GNN-based methods for enrichment analysis. Through a panel of use cases including distinct pathologies and databases, this method provided significant detection of pathways closely related to our case studies. Furthermore, GNNenrich offers novel insights for data interpretation by incorporating weighted scoring based on the contribution of overlap and/or PPIs.

Supported by a context where the ongoing PPIs characterization and related knowledge is rapidly evolving, we aim to improve this method feeding the models with complementary omics data including gene expression, genetic burden, and epigenetic regulation to drive and consolidate the functional pathways enrichment analysis.

## Supplementary Material

btaf478_Supplementary_Data

## Data Availability

The deep learning framework and dependencies are detailed in (Section 16, available as supplementary data at *Bioinformatics* online). To reproduce the results presented in the paper, all necessary data and source code are available in the following repository: https://gitlab.com/cnrgh/gnn-enrich/gnn-enrich-article-demo. This folder includes the trained model along with the scripts necessary for performing the enrichment analysis. To train the model, the code is available on https://github.com/lvguofeng/GNN_PPI.
