# Supplementary material for: GNNenrich: a novel method for pathway enrichment analysis based on graph neural network
Source: Bioinformatics. 2025 Sep 8;41(9):btaf478. doi: 10.1093/bioinformatics/btaf478 (PMC12448840; doi:10.1093/bioinformatics/btaf478)
Supplement: btaf478_Supplementary_Data [file btaf478_supplementary_data.zip › GNNenrich_supplementary_revised.pdf]

Supplementary Materials GNNenrich: a novel method for pathway enrichment analysis based on Graph Neural Network

1 Available annotations in pathways database for Parkinson's disease and gastric cancer queries 3
2 GNN principles and formalism recall 3
3 Protein encoding 3
4 Amino Acid Embeddings 4
5 Hyperparameters fine-tuning 4
6 Recall on scoring methods applied by GNNenrich, g:Profiler and Enrichnet 4
7 Enrichment results for PD on wikiPathways 5
7.1 Top WikiPathways pathways predicted with GNNenrich on the Parkinson list for $\beta = 1$. 5
7.2 Top WikiPathways pathways predicted with GNNenrich on the Parkinson list for $\beta = 10$. 6
7.3 Top WikiPathways pathways predicted with GNNenrich on the Parkinson list for $\beta = 40$. 7
7.4 Pathways predicted specifically with GNNenrich on the Parkinson list for $\beta = 1$. 8
7.5 Pathways predicted specifically with GNNenrich on the Parkinson list for $\beta = 10$. 9
7.6 Pathways predicted specifically with GNNenrich on the Parkinson list for $\beta = 40$. 10
8 Enrichment results for PD on KEGG database 10
8.1 Top KEGG pathways predicted with GNNenrich on the Parkinson list for $\beta = 1$. 10
8.2 Top KEGG pathways predicted with GNNenrich on the Parkinson list for $\beta = 10$. 12
8.3 Top KEGG pathways predicted with GNNenrich on the Parkinson list for $\beta = 40$. 13
8.4 KEGG pathways predicted specifically with GNNenrich on the Parkinson list for $\beta = 10$ 13
8.5 KEGG pathways predicted specifically with GNNenrich on the Parkinson list for $\beta = 40$ 15
9 Enrichment results for PD on Reactome 16
9.1 Top Reactome pathways predicted with GNNenrich on the Parkinson list for $\beta = 1$. 16
9.2 Top Reactome pathways predicted with GNNenrich on the Parkinson list for $\beta = 10$. 17
9.3 Top Reactome pathways predicted with GNNenrich on the Parkinson list for $\beta = 40$. 18
9.4 Reactome pathways predicted specifically with GNNenrich on the Parkinson list for $\beta = 1$ 18
9.5 Reactome pathways predicted specifically with GNNenrich on the Parkinson list for $\beta = 10$ 19
9.6 Reactome pathways predicted specifically with GNNenrich on the Parkinson list for $\beta = 40$ 19
10 Enrichment results for GC on WikiPathways 19
10.1 Top Wikipathways pathways predicted with GNNenrich on the Gastric Cancer for $\beta = 1$. 19
10.2 Top Wikipathways pathways predicted with GNNenrich on the Gastric Cancer for $\beta = 10$. 20
10.3 Top Wikipathways pathways predicted with GNNenrich on the Gastric Cancer for $\beta = 40$. 22
10.4 Pathways predicted with GNNenrich on the Gastric Cancer for $\beta = 1$. 23
10.5 Pathways predicted with GNNenrich on the Gastric Cancer for $\beta = 10$. 23
10.6 Pathways predicted with GNNenrich on the Gastric Cancer for $\beta = 40$. 23
11 Enrichment results for GC on KEGG 24
11.1 Top KEGG pathways predicted with GNNenrich on the Gastric cancer list for $\beta = 1$ 24
11.2 Top KEGG pathways predicted with GNNenrich on the Gastric cancer list for $\beta = 10$ 26
11.3 Top KEGG pathways predicted with GNNenrich on the Gastric cancer list for $\beta = 40$ 27
11.4 KEGG pathways predicted specifically with GNNenrich on the Gastric cancer list for $\beta = 40$ 28
12 Enrichment results for GC on Reactome 29
12.1 Top Reactome pathways predicted with GNNenrich on the Gastric cancer list for $\beta = 1$. 29
12.2 Top Reactome pathways predicted with GNNenrich on the Gastric cancer list for $\beta = 10$. 29
12.3 Top Reactome pathways predicted with GNNenrich on the Gastric cancer list for $\beta = 40$. 30
12.4 Reactome pathways predicted specifically with GNNenrich on the Gastric cancer list for $\beta = 1$ 31
12.5 Reactome pathways predicted specifically with GNNenrich on the Gastric cancer list for $\beta = 10$. Genes overlapping with MalaCards are highlighted in blue when the overlap with the query is empty. 33
12.6 Reactome pathways predicted specifically with GNNenrich on the Gastric cancer list for $\beta = 40$ 33
13 Synthesis of specific results identified by GNNenrich 35
14 Queries for PD and GC 35
15 EnrichNet thresholds 35
16 Computing environment 36
17 Supplementary figures 36
18 The robustness of the GNNenrich. 41

19 Biais relatif to Hub genes 44
20 Statistical significance of the Venn diagram 58
21 Random permutations for sampling the null distribution 60
22 Computational efficiency and scalability of GNNenrich 64
23 Overlap-free GNNenrich results 65
24 Selection of the 10 top and bottom lines of the heatmaps 69

1. Available annotations in pathways database for Parkinson's disease and gastric cancer queries

Parkinson's (PD) disease list [Yu et al. (2010)], comprises 126 genes and 635 PPIs, while the Gastric cancer (GC) [Hamosh et al. (2000); Bamford et al. (2004)] list contains 100 genes and 1829 PPIs. The table below provides a summary of key information regarding the PD and GC queries across the three annotation databases.

Query	Annotation database	# Genes in pathways	#Shared query genes	# PPI with Query
Gastric cancer	KEGG	5245	61	9493
	WikiPathways	7957	79	12042
	Reactome	11155	77	13507
Parkinson	KEGG	5245	75	6598
	WikiPathways	7957	101	8307
	Reactome	11155	98	9491

In the default usage of g:Profiler applied in this study, the annotated genes/proteins associated with pathways databases (KEGG, WikiPathways, Reactome) were utilized as the genomic landscape to calculate the statistical score based on the Fisher test, along with the corresponding p-value. These results were employed for selecting significant pathways.

2. GNN principles and formalism recall

The formulation of GNN could be summarized as follow.
The input graph $G = (P, E, X_P, X_E)$ where $P = \{p_1, p_2, \ldots, p_n\}$ represents the set of nodes, and $E = \{(i, j)|p_i \text{ is adjacent to } p_j\}$ is the set of edges. x_i denotes the feature vector of node p_i and $x_{(i,j)}$ denotes the feature vector of edge $E(i, j)$. The main idea is to describe the state of the node with an accurate embedding vector h_i, that depends on the state information of the neighboring nodes at the previous time. The input graph G is converted into a dynamic graph $G^t = (P, E, X_P, X_E, H^t)$ in the graph neural network model, where $t = 1, 2, \ldots, T$ represents time and $H^t = (h_1^{(t)}, h_2^{(t)}, \ldots, h_n^{(t)}), h_i^{(t)}$ represents the state vector of node p_i at time t, which depends on the graph $G^{(t-1)}$ at time $t - 1$. The equation of h_i^t is as follows:

$$h_i^t = f_w(x_i, x_{ne(i)}, h_{ne(i)}^{(t-1)}) \quad (1)$$

Where $ne(i)$ is the set of all nodes adjacent to node p_i and f_w denotes the transformation function with parameter w.

In this context, each interaction extracted from STRING is represented as a vector of seven binary values, indicating whether specific types of interaction data are present or absent. The dataset, comprising 3,470,907 interactions, is structured into 544,538 edges that collectively define the set E.

3. Protein encoding

The design of the GNNenrich framework enables the use of protein embedding as input generated from different models and methods. Here, the work published by Lv et al. (2021) was used to formalize the previous protein encoding before enrichment analysis based on embedding vectors correlations.

In order to learn an optimal representation of proteins, Lv et al. (2021) suggested in his work to integrate simultaneously in the model the amino acid sequence x_i as an independent feature of the protein P_i and the features of neighboring nodes $h_{ne(i)}$. More precisely, on these bases the GNN of the t-th iteration can be defined as follows:

$$h_i^t = Update(x_i^t, Aggregate(x_{ne(i)}^{t-1}), h_{ne(i)}^{(t-1)}) \quad (2)$$

x_i^t is the embedding of amino acid sequences described in the section 4.

The model updates the state of node h_i^t iteratively to optimize the parameter w and predict the set of appropriate labels $y(i, j)$ for a given interaction $E(i, j)$. This supervised learning task reduces the multi-label cross-entropy defined by the following expression where P_{train} is a protein training set.

$$L = \sum_{k=0}^{n}(\sum_{P_{train}} y^k(i,j)log(P(y^k(i,j))) - (1 - y^k(i,j))log(1 - P(y^k(i,j))) \quad (3)$$

To summarize, the protein encoding proposed by Lv et al. (2021) gather two distinct Neural Network modules (figure 1) that simultaneously learn to reduce cross-entropy error and optimize model parameters to accurately predict multi-class interactions between proteins.

The first module correlates the intrinsic features of proteins using convolution layers, BiGRU (Bidirectional Gated Recurrent Unit) layer and pooling. The second module includes three graph convolution layers (GCN). The GCN uses normalized aggregation [Kipf and Welling (2016)] and therefore defines the message propagation function as follows:

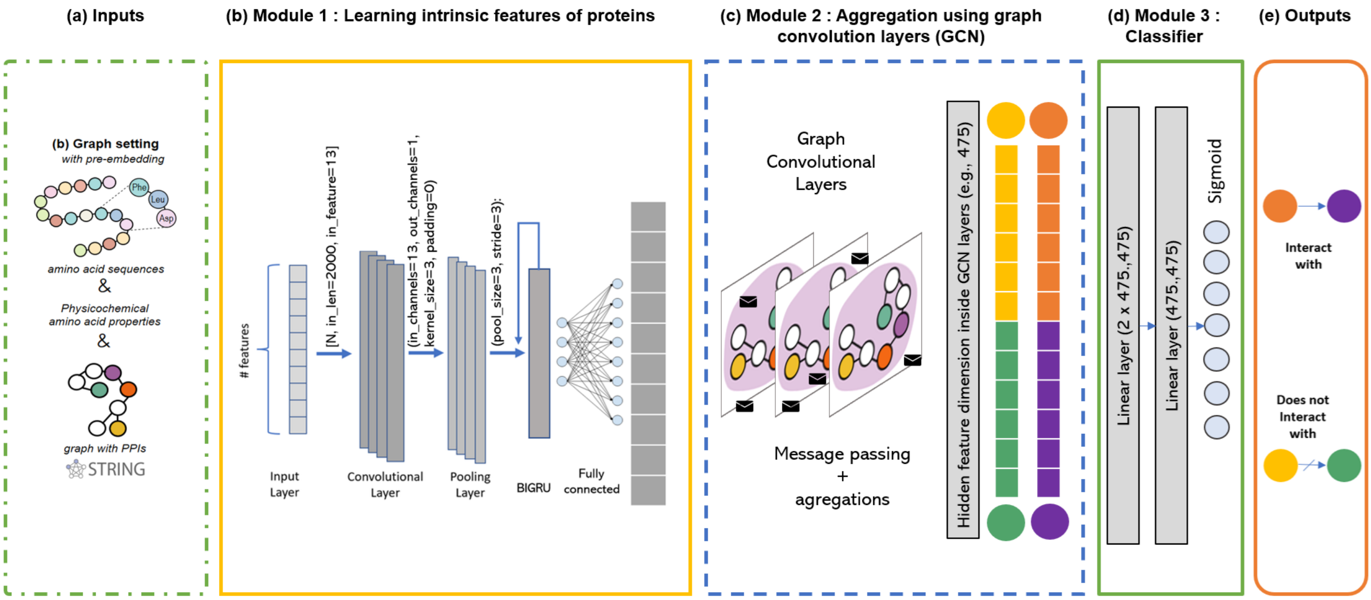

Fig. 1: The architecture of GNNenrich model. The first module analyzes the intrinsic features of proteins using convolution layers, a Bidirectional GRU (BiGRU) layer, and pooling. The second module applies three graph convolution layers (GCN) that utilize normalized aggregation to define the message propagation function. Proteins encoding is extracted from the second module. The third module takes as input the concatenated embeddings of two proteins. A Sigmoid function is applied to the output, which maps the prediction to a value between 0 and 1, indicating the probability of interaction for each of the 7 possible types.

$$h_i^t = \sigma(W^{(t)} \sum_{j \in \{ne(i) \cup i\}} (\frac{h_j^{(t-1)}))}{\sqrt{|N(i)||N(j)|}}) \tag{4}$$

Where N is the node degree to normalize the aggregation operation and σ is ReLU (Rectified Linear Units) activation function.

The training set was generated with a random selection of (80%) from the set of edges E. 435,630 are used to train the model and 108,908 for the validation. This random selection leads to a majority of interactions belonging to pair of proteins where each protein participating to the interaction are seen during the training. This training setting is referred as a "Both Seen" (BS) in [Lv et al. (2021)]. During the training process, the parameters of the model were updated through a learning rate of 10^{-3}.

4. Amino Acid Embeddings

Inspired by natural language processing (NLP) modeling tasks, Chen et al. (2019) introduced an efficient property-embedding method to capture the contextual and physicochemical relationships among amino acids. Each amino acid embedding vector is a concatenation of two sub-embeddings. The first component captures the co-occurrence similarity between amino acids obtained by training the Skip-Gram model [Mikolov et al. (2013)] on protein sequences where the vocabulary samples are overlapping 3-mer amino acids, and the word vector size is 5. The second component is a one-hot encoding based on amino acid classification by electrostaticity and hydrophobicity similarity. According to [Shen et al. (2007)], the 20 natural amino acids are grouped into 7 classes. The 21st amino acid U(Selenocysteine), the 22nd amino acid O(Pyrrolysine) and the unknown amino acids X are included in the eighth category as suggested in [Lv et al. (2021); Wu et al. (2023)]. The resulting size of the amino acid embeddings is 13: 5 coming from the Skip-Gram model and 8 from the one hot encoding.

5. Hyperparameters fine-tuning

To determine the optimal size of the protein descriptor vector denoted by h_P, we conducted an automated fine-tuning operation on GNNenrich using Bayesian optimization [Gardner et al. (2014)].

The study focuses on a range from 100 to 1000, where the model's F1 score fluctuates between 85.99 and 89.52 (table 1). The best performance is achieved for an embedding of size 475. This size will be used to describe all the proteins of the v11 STRING database.

6. Recall on scoring methods applied by GNNenrich, g:Profiler and Enrichnet

GNNenrich generates scoring from correlation computation obtained from pathways embedding and query comparison. In order to statistically assess this correlation/enrichment scores, we sampled 100,000 random lists from gene/proteins referenced in the STRING

Table 1. Bayesian optimization results for embedding size tuning: Performance variation according to the embedding size.

Embedding size	475	748	100	372	232	183
Accuracy	89.52	88.55	85.99	88.44	87.28	86.73

database fitting the number of the genes/proteins included in our two queries GC and PD. For each random list obtained, a score was computed for each pathway. The empirical null distribution of the scores obtained by random sampling was used to estimate the p-value of the observed score obtained for that pathway. According to our current number of permutation, the minimal significant observed p-value is 0.00001. The p-values were then corrected with the Benjamini-Hochberg (BH) procedure to control for the False Discovery Rate (FDR).

In the default usage of g:Profiler applied in this study, the annotated genes/proteins associated with pathways databases (KEGG, WikiPathways, Reactome) were utilized as the genomic landscape to calculate the statistical score based on the Fisher test, along with the corresponding p-value. These results were employed for selecting significant pathways. The table summarizing the size of databases can be found in section 1.

The scoring of EnrichNet are based on the measure of distance obtained from a random walk algorithm. From the distance distribution obtained for all pathways referenced in the databases (KEGG, WikiPathways, Reactome) and Fisher's exact test obtained with classical over-representation scores for overlapping datasets, a linear regression is applied to determine a threshold for significant pathways selection. The threshold applied for each use cases are referenced in the following table 37.

7. Enrichment results for PD on wikiPathways

7.1. Top WikiPathways pathways predicted with GNNenrich on the Parkinson list for $\beta = 1$.

Table 2. Top 20 WikiPathways pathways predicted with GNNenrich on the Parkinson list for $\beta = 1$ and sorted by BH and g:Profiler score. All these pathways are significant with GNNenrich.

Pathway Name	BH	Size	Overlap	PPI	g:Profiler	EnrichNet	Gene in the overlap
MAMMARY GLAND DEVELOPMENT PATHWAY PUBERTY STAGE 2 OF 4	**1.22E-03****	13	1	66	2.85E-01	-2.00E-01	ESR1
IL7 SIGNALING PATHWAY	**1.22E-03****	25	1	130	3.90E-01	3.00E-01	GSK3B
EGFR TYROSINE KINASE INHIBITOR RESISTANCE	**1.22E-03****	84	2	362	4.08E-01	-5.29E-02	GSK3B;IL6
SCFA AND SKELETAL MUSCLE SUBSTRATE METABOLISM	**1.22E-03****	6	0	24	NA	-2.00E-01	
BMP2WNT4FOXO1 PATHWAY IN PRIMARY ENDOMETRIAL STROMAL CELL DIFFERENTIATION	**1.22E-03****	13	0	35	NA	-2.00E-01	
OSTEOBLAST SIGNALING	**1.22E-03****	14	0	35	NA	-2.00E-01	
EV RELEASE FROM CARDIAC CELLS AND THEIR FUNCTIONAL EFFECTS	**3.14E-03****	11	1	42	2.61E-01	-2.00E-01	TNF
OSTEOCLAST SIGNALING	**3.25E-03****	16	0	40	NA	-2.00E-01	
BMP SIGNALING IN EYELID DEVELOPMENT	**3.25E-03****	20	0	80	NA	-2.00E-01	
OSTEOPONTIN SIGNALING	**3.66E-03****	13	0	74	NA	-2.00E-01	
NAD METABOLISM SIRTUINS AND AGING	**3.90E-03****	11	1	61	2.61E-01	-2.00E-01	TFAM
HORMONAL CONTROL OF PUBERTAL GROWTH SPURT	**3.90E-03****	5	0	23	NA	-2.00E-01	
ANGIOTENSIN II RECEPTOR TYPE 1 PATHWAY	**3.90E-03****	28	0	95	NA	-2.00E-01	
ENDOCHONDRAL OSSIFICATION WITH SKELETAL DYSPLASIAS	**3.90E-03****	63	0	168	NA	-2.00E-01	
ENDOCHONDRAL OSSIFICATION	**3.90E-03****	63	0	168	NA	-2.00E-01	
MIRNAS INVOLVED IN DNA DAMAGE RESPONSE	**5.03E-03****	68	0	74	NA	-2.00E-01	
CALORIC RESTRICTION AND AGING	**6.89E-03****	8	0	77	NA	-2.00E-01	
FOXP3 IN COVID19	**6.91E-03****	15	1	55	3.13E-01	-2.00E-01	IL6
LET7 INHIBITION OF ES CELL REPROGRAMMING	**6.93E-03****	15	0	22	NA	-2.00E-01	
SLEEP REGULATION	**7.99E-03****	38	6	141	1.24E-04	2.09E-01	DRD3;HTR2A; IL6;TH;CST3; DRD2

7.2. Top WikiPathways pathways predicted with GNNenrich on the Parkinson list for $\beta = 10$.

Table 3. Top 20 WikiPathways pathways predicted with GNNenrich on the Parkinson list for $\beta = 10$. These pathways have a p-value $p < 10E^{-5}$ and $BH = 1.61E^{-04}$ and are sorted according to g:Profiler p-values. All these pathways are significant with GNNenrich and g:Profiler.

Pathway Name	BH	Size	Overlap	PPI	g:Profiler	EnrichNet	Gene in the overlap
MALE INFERTILITY	**1.61E-04***	145	14	227	**3.61E-07**	6.085E-01	NQO1;ESR1;TNF;ABCB1; MTR; PON1;CYP1A1; NOS1;MTHFR; NOS3;PACRG; POLG;SOD2;ESR2
SUDDEN INFANT DEATH SYNDROME SIDS SUSCEPTIBILITY PATHWAYS	**1.61E-04***	159	15	469	**3.61E-07**	5.426E-01	IL10;IL1B; TNF;MAOA;CHRNA4; LMX1B; SLC6A4; IL1RN;TH; HTR2A;ASCL1; IL1A; ESR2;IL6;BDNF
DOPAMINERGIC NEUROGENESIS	**1.61E-04***	30	8	62	**3.61E-07**	**2.050E+00**	MSX1;LMX1B;PITX3; SLC6A3; NR4A2;TH; ASCL1;LMX1A
METAPATHWAY BIOTRANSFORMATION PHASE I AND II	**1.61E-04***	183	15	471	**6.86E-07**	5.195E-01	CYP2E1;GSTM3;HNMT;GSTZ1; CYP1A1;GSTO2; GSTP1;COMT; NAT2;GSTM1;GSTO1; EPHX1;CYP1B1;CYP1A2;CYP2D6
PARKINSONS DISEASE PATHWAY	**1.61E-04***	71	10	102	**1.16E-06**	**2.320E+00**	UCHL1;SNCAIP; SNCA;PARK7; ATXN2;LRRK2; SLC6A3;TH; HTRA2;PINK1
ESTROGEN METABOLISM WP697	**1.61E-04***	18	6	93	**3.41E-06**	**2.800E+00**	NQO1;CYP1A1;COMT; GSTM1; CYP1B1;CYP1A2
NEUROTRANSMITTER DISORDERS	**1.61E-04***	10	5	13	**3.53E-06**	**4.300E+00**	MAOA;SLC6A3; TH;COMT;DBH
MONOAMINE TRANSPORT	**1.61E-04***	32	7	91	**4.83E-06**	1.085E+00	IL1B;TNF;SLC6A4; SLC6A3;TH;NOS1;DBH
BIOSYNTHESIS AND REGENERATION OF TETRAHYDROBIOPTERIN AND CATABOLISM OF PHENYLALANINE	**1.61E-04***	11	5	9	**4.99E-06**	**3.400E+00**	MAOB;GCH1;MAOA;COMT;DBH
VITAMIN B12 METABOLISM	**1.61E-04***	52	7	193	**7.15E-06**	8.583E-01	IL1B;TNF;SERPINA3; MTR;APOE;SOD2; MTHFR;IL6
DOPAMINE METABOLISM	**1.61E-04***	13	5	63	**9.58E-06**	**2.800E+00**	MAOB;NQO1; MAOA;TH;COMT
MELATONIN METABOLISM AND EFFECTS	**1.61E-04***	37	7	137	**9.58E-06**	**1.845E+00**	MAOA;GSK3B; APOE;CYP1A1;CYP1B1; CYP1A2;CYP2D6
BENZENE METABOLISM	**1.61E-04**	6	4	28	**9.58E-06**	**4.300E+00**	CYP2E1;NQO1; GSTM1;EPHX1
RAS AND BRADYKININ PATHWAYS IN COVID19	**1.61E-04***	29	6	150	**3.34E-05**	**1.514E+00**	IL1B;TNF; NOS1;NOS3;IL1A; ACE
NUCLEAR RECEPTORS METAPATHWAY	**1.61E-04***	314	16	759	**4.15E-05**	2.068E-01	HMOX1;NQO1; ESR1;IL1B; TNF;GSTM3;ABCB1; SLC6A4; FTL;SLC6A3;CYP1A1;GSTP1; GSTM1;CYP1B1;CYP1A2;FTH1
MONOAMINE GPCRS	**1.61E-04***	33	6	131	**6.54E-05**	**1.436E+00**	DRD5;ADRA2A; DRD3;DRD4; HTR2A;DRD2
NICOTINE EFFECT ON DOPAMINERGIC NEURONS	**1.61E-04***	21	5	93	**9.93E-05**	**1.600E+00**	DRD3;DRD4;CHRNA4;TH;DRD2
TAMOXIFEN METABOLISM	**1.61E-04***	21	5	114	**9.93E-05**	9.245E-01	CYP2E1;CYP1A1; CYP1B1;CYP1A2;CYP2D6
ELECTRON TRANSPORT CHAIN OXPHOS SYSTEM IN MITOCHONDRIA	**1.61E-04***	103	9	339	**1.02E-04**	-6.813E-02	MT-CYB;MT-ND4;MT-ND5; MT-ND1;NDUFV2; MT-CO2; MT-CO1;MT-ND3; MT-ND2
SLEEP REGULATION	**1.61E-04***	38	6	141	**1.24E-04**	2.086E-01	CST3;DRD3;TH;HTR2A;DRD2;IL6

7.3. Top WikiPathways pathways predicted with GNNenrich on the Parkinson list for $\beta = 40$.

Table 4. Top 20 WikiPathways pathways predicted with GNNenrich on the Parkinson list for $\beta = 40$. These pathways have a p-value $p < 10E^{-5}$ and are sorted according to g:Profiler p-values. All these pathways are significant with GNNenrich and g:Profiler.

Pathway Name	BH	Size	Overlap	PPI	g:Profiler	EnrichNet	Gene in the overlap
SUDDEN INFANT DEATH SYNDROME SIDS SUSCEPTIBILITY PATHWAYS	**1.38E-04*****	159	15	469	**3.61E-07**	5.43E-01	ESR2;LMX1B;MAOA; IL10;HTR2A; TNF;ASCL1; IL1B;SLC6A4;IL6; TH;BDNF;CHRNA4; IL1RN;IL1A
MALE INFERTILITY	**1.38E-04*****	145	14	227	**3.61E-07**	6.09E-01	ESR2;SOD2;PON1;PACRG; TNF;POLG;MTR; NOS1;ESR1;NQO1; CYP1A1;NOS3; ABCB1;MTHFR
DOPAMINERGIC NEUROGENESIS	**1.38E-04*****	30	8	62	**3.61E-07**	**2.05E+00**	LMX1B;MSX1;PITX3; SLC6A3;ASCL1; LMX1A;NR4A2;TH
METAPATHWAY BIOTRANSFORMATION PHASE I AND II	**1.38E-04*****	183	15	471	**6.86E-07**	5.20E-01	NAT2;GSTM3;GSTZ1; CYP2D6;GSTM1; GSTP1;CYP1B1;GSTO2; GSTO1;CYP2E1;HNMT; CYP1A1;CYP1A2;EPHX1;COMT
PARKINSONS DISEASE PATHWAY	**1.38E-04*****	71	10	102	**1.16E-06**	**2.32E+00**	UCHL1;SNCA;SNCAIP; SLC6A3;PARK7; PINK1;HTRA2;ATXN2; TH;LRRK2
ESTROGEN METABOLISM WP697	**1.38E-04*****	18	6	93	**3.41E-06**	**2.80E+00**	GSTM1;CYP1B1; NQO1; CYP1A2;CYP1A1;COMT
NEUROTRANSMITTER DISORDERS	**1.38E-04*****	10	5	13	**3.53E-06**	**4.30E+00**	DBH;SLC6A3;TH;COMT;MAOA
MONOAMINE TRANSPORT	**1.38E-04*****	32	7	91	**4.83E-06**	1.09E+00	TNF;DBH;SLC6A3; NOS1;IL1B;SLC6A4;TH
BIOSYNTHESIS AND REGENERATION OF TETRAHYDROBIOPTERIN AND CATABOLISM OF PHENYLALANINE	**1.38E-04*****	11	5	9	**4.99E-06**	**3.40E+00**	MAOB;DBH;GCH1;COMT;MAOA
VITAMIN B12 METABOLISM	**1.38E-04*****	51	7	193	**7.15E-06**	8.58E-01	SOD2;SERPINA3;TNF; MTR;IL1B; IL6;APOE;MTHFR
DOPAMINE METABOLISM	**1.38E-04*****	13	5	63	**9.58E-06**	**2.80E+00**	MAOB;NQO1;TH;COMT;MAOA
MELATONIN METABOLISM AND EFFECTS	**1.38E-04*****	37	7	137	**9.58E-06**	**1.84E+00**	CYP2D6;CYP1B1;CYP1A2;APOE; CYP1A1;GSK3B;MAOA
BENZENE METABOLISM	**1.38E-04*****	6	4	28	**9.58E-06**	**4.30E+00**	EPHX1;GSTM1;CYP2E1;NQO1
RAS AND BRADYKININ PATHWAYS IN COVID19	**1.38E-04*****	29	6	150	**3.34E-05**	**1.51E+00**	TNF;ACE;NOS1;IL1B;NOS3;IL1A
NUCLEAR RECEPTORS METAPATHWAY	**1.38E-04*****	314	16	759	**4.15E-05**	2.07E-01	GSTM3;FTH1;GSTM1; TNF;HMOX1; SLC6A3;GSTP1;CYP1B1; ESR1;FTL;SLC6A4; NQO1;IL1B;CYP1A1 ;CYP1A2;ABCB1
MONOAMINE GPCRS	**1.38E-04*****	33	6	131	**6.54E-05**	**1.44E+00**	DRD2;HTR2A;DRD5; DRD3;DRD4;ADRA2A
NICOTINE EFFECT ON DOPAMINERGIC NEURONS	**1.38E-04*****	21	5	93	**9.93E-05**	**1.60E+00**	DRD2;DRD3;CHRNA4;DRD4;TH
ELECTRON TRANSPORT CHAIN OXPHOS SYSTEM IN MITOCHONDRIA	**1.38E-04*****	103	9	339	**1.02E-04**	-6.81E-02	MT-CO2;MT-ND4;MT-ND3; MT-ND2;NDUFV2; MT-CYB;MT-ND5; MT-ND1;MT-CO1
SLEEP REGULATION	**1.38E-04*****	38	6	141	**1.24E-04**	2.09E-01	DRD2;HTR2A;DRD3;IL6;TH;CST3
SEROTONIN TRANSPORTER ACTIVITY	**1.38E-04*****	11	4	39	**1.24E-04**	**3.66E+00**	IL1B;MAOA;SLC6A4;NOS1

7.4. Pathways predicted specifically with GNNenrich on the Parkinson list for $\beta = 1$.

Table 5. Selection of WikiPathways pathways predicted specifically with GNNenrich with $\beta = 1$ for the PD use case.

Pathway Name	BH	Size	Overlap	PPI	g:Profiler	EnrichNet	Gene in the overlap
IL18 SIGNALING PATHWAY	**9.37E-03** **	273	8	686	7.90E-02	-4.83E-04	HMOX1;GRN;GSK3B;TNF;IL6; IL1B;GRIN2B;IL10
MAPK SIGNALING PATHWAY	**3.35E-02***	247	8	694	5.05E-02	1.21E-01	LRRK2;IL1A;TNF; CACNA1A;BDNF; MAPT;IL1B;FGF20
MYOMETRIAL RELAXATION AND CONTRACTION PATHWAYS	**1.72E-02***	156	5	558	1.40E-01	4.95E-02	NOS3;GSTO1;IL6;IL1B;NOS1
PI3KAKT SIGNALING PATHWAY	**1.72E-02***	339	5	1015	5.19E-01	-8.31E-02	NOS3;GSK3B;BDNF;IL6;FGF20
NETWORK MAP OF SARSCOV2 SIGNALING PATHWAY	**4.85E-02***	218	5	622	2.74E-01	-7.72E-02	IL1A;TNF;IL6;IL1B;IL10
FOCAL ADHESION PI3KAKTMTORSIGNALING PATHWAY	**3.04E-02***	302	4	875	6.02E-01	-7.37E-02	GSK3B;NOS3;FGF20;NOS1
TNFRELATED WEAK INDUCER OF APOPTOSIS TWEAK SIGNALING PATHWAY	**1.24E-02***	42	3	236	5.99E-02	9.95E-02	GSK3B;TNF;IL6
VITAMIN D RECEPTOR PATHWAY	**2.06E-02***	185	3	282	5.13E-01	-1.21E-01	CYP2D6;ABCB1;CYP1A1
NEURAL CREST DIFFERENTIATION	**2.06E-02***	101	3	168	2.67E-01	6.42E-02	GSK3B;ASCL1;MSX1
EGFR TYROSINE KINASE INHIBITOR RESISTANCE	**1.22E-03****	84	2	362	4.08E-01	-5.29E-02	GSK3B;IL6
ADIPOGENESIS	**8.23E-03****	131	2	343	5.69E-01	-2.00E-01	TNF;IL6
IL6 SIGNALING PATHWAY	**1.18E-02***	43	2	216	2.36E-01	1.33E-01	GSK3B;IL6
TRANSCRIPTION FACTOR REGULATION IN ADIPOGENESIS	**1.24E-02***	22	2	142	9.83E-02	-2.00E-01	TNF;IL6
OXYSTEROLS DERIVED FROM CHOLESTEROL	**1.72E-02***	48	2	41	2.56E-01	3.64E-02	ESR2;ESR1
PRADERWILLI AND ANGELMAN SYNDROME	1.72E-02	68	2	129	3.47E-01	-2.00E-01	BDNF;MSX1
EMBRYONIC STEM CELL PLURIPOTENCY PATHWAYS	**3.35E-02***	116	2	328	5.21E-01	-4.83E-04	GSK3B;FGF20
MAMMARY GLAND DEVELOPMENT PATHWAY PUBERTY STAGE 2 OF 4	**1.22E-03****	13	1	66	2.85E-01	-2.00E-01	ESR1
IL7 SIGNALING PATHWAY	**1.22E-03****	25	1	130	3.90E-01	3.00E-01	GSK3B
EV RELEASE FROM CARDIAC CELLS AND THEIR FUNCTIONAL EFFECTS	**3.14E-03****	11	1	42	2.61E-01	-2.00E-01	TNF
NAD METABOLISM SIRTUINS AND AGING	**3.90E-03****	11	1	61	2.61E-01	-2.00E-01	TFAM
FOXP3 IN COVID19	**6.91E-03****	15	1	55	3.13E-01	-2.00E-01	IL6
MIRNA REGULATION OF PROSTATE CANCER SIGNALING PATHWAYS	**7.99E-03****	49	1	151	5.45E-01	1.91E-01	GSK3B
MAMMARY GLAND DEVELOPMENT PATHWAY EMBRYONIC DEVELOPMENT STAGE 1 OF 4	1.40E-02	18	1	68	3.39E-01	-2.00E-01	TNF
GALANIN RECEPTOR PATHWAY	**1.72E-02***	21	1	134	3.62E-01	-2.00E-01	IL6
RELATIONSHIP BETWEEN INFLAMMATION COX2 AND EGFR	**1.72E-02***	25	1	146	3.90E-01	-2.00E-01	ESR1
LNCRNAMEDIATED MECHANISMS OF THERAPEUTIC RESISTANCE	**1.72E-02***	11	1	41	2.61E-01	-2.00E-01	ABCB1
NCRNAS INVOLVED IN WNT SIGNALING IN HEPATOCELLULAR CARCINOMA	**2.06E-02***	89	1	132	7.25E-01	-7.00E-02	GSK3B
ERBB SIGNALING PATHWAY	**2.50E-02***	92	1	365	7.34E-01	-5.05E-02	GSK3B
TGFBETA RECEPTOR SIGNALING IN SKELETAL DYSPLASIAS	**2.62E-02***	59	1	187	5.98E-01	-2.00E-01	TNF
ANGIOGENESIS	**2.79E-02***	24	1	167	3.80E-01	3.29E-01	NOS3
NEURAL CREST CELL MIGRATION DURING DEVELOPMENT	**3.25E-02***	40	1	156	5.04E-01	-2.00E-01	BDNF
NCRNAS INVOLVED IN STAT3 SIGNALING IN HEPATOCELLULAR CARCINOMA	**3.46E-02***	17	1	56	3.31E-01	-2.00E-01	IL6
ESTROGEN SIGNALING PATHWAY	**4.30E-02***	23	1	184	3.72E-01	-2.00E-01	ESR1
TGFBETA RECEPTOR SIGNALING	**4.85E-02***	55	1	182	5.76E-01	-2.00E-01	TNF

7.5. Pathways predicted specifically with GNNenrich on the Parkinson list for $\beta = 10$.

GNNenrich results on WikiPathways (Table 6) highlight several pathways that are linked to the regulation of neuronal processes and a range of neurological conditions, including those related to "Brain-Derived Neurotrophic Factor (BDNF) Signaling Pathway" [Murer et al. (2001)], "Serotonin and Anxiety" [Joling et al. (2018)], "Neural Crest Differentiation" [Yang et al. (2017a)], "Netrin-UNC5B Signaling Pathway" [Ahn et al. (2021)], "Synaptic Signaling Pathways Associated with Autism Spectrum Disorder" [Picconi et al. (2012); Schirinzi et al. (2016)] and "Phosphodiesterases in Neuronal Function" [Morales-Garcia et al. (2011); Niccolini et al. (2017)].

For overlap-free results supported only by PPIs "The Caloric Restriction and Aging pathway" is notably identified [Srivastava and C Haigis (2011)]. It is recognized for its impact on aging and neurodegenerative diseases by modulating processes like inflammation and oxidative stress [de Carvalho (2022); Maswood et al. (2004)] .

Table 6. Selection of WikiPathways pathways predicted specifically with GNNenrich with $\beta = 10$ for the PD use case. Genes overlapping with MalaCards are highlighted in blue when the overlap with the query is empty.

Pathway Name	BH	Size	Overlap	PPI	g:Profiler	EnrichNet	Gene in the overlap
MAPK SIGNALING PATHWAY	**2.93E-04***	247	8	694	5.05E-02	1.21E-01	IL1B;TNF;FGF20;LRRK2; CACNA1A;IL1A;MAPT;BDNF
GPCRS CLASS A RHODOPSINLIKE	**4.69E-03**	260	8	708	6.34E-02	9.14E-02	CCKBR;DRD5;ADRA2A;DRD3; DRD4;CCKAR;HTR2A;DRD2
IL18 SIGNALING PATHWAY	**1.44E-03**	273	8	686	7.90E-02	-4.83E-04	HMOX1;IL10;IL1B;TNF; GRN;GSK3B;GRIN2B;IL6
BRAINDERIVED NEUROTROPHIC FACTOR BDNF SIGNALING PATHWAY	**3.18E-02***	145	5	499	1.08E-01	1.03E-01	LINGO1;GSK3B;GRIN2B; MAPT;BDNF
MYOMETRIAL RELAXATION AND CONTRACTION PATHWAYS	**3.02E-03**	156	5	558	1.40E-01	4.95E-02	IL1B;NOS1;GSTO1;NOS3;IL6
NETWORK MAP OF SARSCOV2 SIGNALING PATHWAY	**1.64E-02***	218	5	622	2.74E-01	-7.72E-02	IL10;IL1B;TNF;IL1A;IL6
AMINO ACID METABOLISM	**1.16E-02***	91	4	108	9.48E-02	1.10E-01	HNMT;MAOA;DBH;TH
GPCRS OTHER	**2.37E-02***	93	4	212	9.83E-02	1.15E-01	HTR2A;DRD4;DRD3;CCKBR
BURN WOUND HEALING	**7.32E-03**	113	4	357	1.53E-01	4.61E-02	IL1A;IL6;IL1B;TNF
2Q37 COPY NUMBER VARIATION SYNDROME	**9.78E-03**	145	4	258	2.44E-01	6.41E-03	ASCL1;USP40;IL1B;TNF
BREAST CANCER PATHWAY	**1.76E-02***	154	4	440	2.61E-01	2.08E-02	FGF20;ESR1;ESR2;GSK3B
OSTEOBLAST SIGNALING	**1.22E-02***	14	0	35	NA	-2.00E-01	['PDGFB';'PDGFRB']
OSTEOCLAST SIGNALING	**1.51E-02** *	16	0	40	NA	-2.00E-01	['AIMP2', 'IFNB1', 'MAPK8', 'PDGFB', 'SPP1']
SCFA AND SKELETAL MUSCLE SUBSTRATE METABOLISM	**1.90E-02***	6	0	24	NA	-2.00E-01	['PPARD','FFAR2', 'FFAR3', 'GCG', 'PYY','SLC2A4']
BMP2WNT4FOXO1 PATHWAY IN PRIMARY ENDOMETRIAL STROMAL CELL DIFFERENTIATION	**2.29E-02***	13	0	35	NA	-2.00E-01	[BCL2L11, BMP2, CTNNB1, DKK1, FOXO1, SST]
LET7 INHIBITION OF ES CELL REPROGRAMMING	**4.49E-02***	15	0	22	NA	-2.00E-01	['EGR1','KLF4', 'MIRLET7C','MIRLET7D' ,'MIRLET7G','MIRLET7I', 'MYC','POU5F1', 'SOX2']
OSX AND MIRNAS IN TOOTH DEVELOPMENT	**4.56E-02***	34	0	32	NA	-2.00E-01	['CTNNB1', 'DKK1', 'KLF4', 'MIR145', 'MIR204', 'MIR29B1', 'MIR338', 'MIRLET7C', 'MIRLET7D', 'MIRLET7G', 'MIRLET7I', 'NOTCH1', 'RUNX2']
ANGIOTENSIN II RECEPTOR TYPE 1 PATHWAY	**4.65E-02***	28	0	95	NA	-2.00E-01	['ACE2', 'AGT', 'AGTR1', 'HIF1A', 'MAPK1', 'NOX4', 'SMAD3', 'SP1', 'TGFB1']
CALORIC RESTRICTION AND AGING	**4.75E-02***	8	0	77	NA	-2.00E-01	['AKT1', 'IGF1', 'MTOR', 'NAMPT', 'PPARGC1A', 'SIRT1', 'TP53']
BMP SIGNALING IN EYELID DEVELOPMENT	**4.82E-02***	20	0	80	NA	-2.00E-01	['BMP4', 'EGFR', 'FGF10', 'JUN', 'MAP3K1', 'MAPK3', 'MAPK9', 'NOTCH1', 'SHH', 'TGFA']
MICRORNA NETWORK ASSOCIATED WITH CHRONIC LYMPHOCYTIC LEUKEMIA	**4.86E-02***	10	0	26	NA	-2.00E-01	['BCL2', 'MCL1', 'MIR34A', 'MIR34B', 'MIR34C', 'TP53']

7.6. Pathways predicted specifically with GNNenrich on the Parkinson list for $\beta = 40$.

Table 7. Selection of WikiPathways pathways predicted specifically with GNNenrich with $\beta = 40$ for the PD use case.

Pathway Name	BH	Size	Overlap	PPI	g:Profiler	EnrichNet	Gene in the overlap
MAPK SIGNALING PATHWAY	**2.52E-04*****	247	8	694	5.05E-02	1.21E-01	CACNA1A;MAPT;FGF20;TNF; IL1B;BDNF;LRRK2;IL1A
IL18 SIGNALING PATHWAY	**4.72E-04*****	273	8	686	7.90E-02	-4.83E-04	IL10;TNF;HMOX1;IL1B;GRIN2B; IL6;GSK3B;GRN
GPCRS CLASS A RHODOPSINLIKE	**2.31E-03****	260	8	708	6.34E-02	9.14E-02	DRD4;DRD2;HTR2A;CCKBR; DRD5;DRD3;CCKAR;ADRA2A
MYOMETRIAL RELAXATION AND CONTRACTION PATHWAYS	**6.70E-03****	156	5	558	1.40E-01	4.95E-02	NOS1;IL1B;GSTO1;IL6;NOS3
BRAINDERIVED NEUROTROPHIC FACTOR BDNF SIGNALING PATHWAY	**2.03E-02***	144	5	499	1.08E-01	1.03E-01	MAPT;LINGO1;GRIN2B;GSK3B; BDNF
NETWORK MAP OF SARSCOV2 SIGNALING PATHWAY	**2.54E-02***	218	5	622	2.74E-01	-7.72E-02	IL10;TNF;IL1B;IL6;IL1A
BURN WOUND HEALING	**6.96E-03****	113	4	357	1.53E-01	4.61E-02	IL1B;TNF;IL6;IL1A
AMINO ACID METABOLISM	**1.02E-02***	91	4	108	9.48E-02	1.10E-01	MAOA;TH;DBH;HNMT
2Q37 COPY NUMBER VARIATION SYNDROME	**1.34E-02***	145	4	258	2.44E-01	6.41E-03	ASCL1;TNF;IL1B;USP40
GPCRS OTHER	**1.36E-02***	93	4	212	9.83E-02	1.15E-01	DRD4;HTR2A;CCKBR;DRD3
BREAST CANCER PATHWAY	**2.55E-02***	154	4	440	2.61E-01	2.08E-02	ESR2;ESR1;FGF20;GSK3B
TNFRELATED WEAK INDUCER OF APOPTOSIS TWEAK SIGNALING PATHWAY	**2.05E-03****	42	3	236	5.99E-02	9.95E-02	GSK3B;TNF;IL6
SYNAPTIC VESICLE PATHWAY	**2.49E-02***	51	3	47	9.12E-02	3.00E-01	SLC6A4;CACNA1A;PARK7
APOPTOSISRELATED NETWORK DUE TO ALTERED NOTCH3 IN OVARIAN CANCER	**2.53E-02***	53	3	182	9.70E-02	2.61E-01	TNF;NQO1;APOE
NEURAL CREST DIFFERENTIATION	**2.82E-02***	101	3	168	2.67E-01	6.42E-02	ASCL1;MSX1;GSK3B
ULCERATIVE COLITIS SIGNALING	**6.43E-03****	18	2	78	7.63E-02	-2.00E-01	TNF;IL10
PI3KAKTMTOR VITAMIN D3 SIGNALING	**6.78E-03****	22	2	80	9.83E-02	4.92E-01	GSK3B;IL10
TYPE II DIABETES MELLITUS	**1.09E-02***	22	2	74	9.83E-02	7.00E-01	TNF;CACNA1A
CODEINE AND MORPHINE METABOLISM	**1.97E-02***	14	2	67	5.05E-02	4.92E-01	CYP2D6;ABCB1
IL5 SIGNALING PATHWAY	**2.03E-02***	40	2	202	2.16E-01	5.50E-01	MAPT;GSK3B
IL6 SIGNALING PATHWAY	**2.06E-02***	43	2	216	2.36E-01	1.33E-01	GSK3B;IL6
VITAMIN D IN INFLAMMATORY DISEASES	**2.14E-02***	22	2	106	9.83E-02	-2.00E-01	TNF;IL6
T CELL MODULATION IN PANCREATIC CANCER	**2.71E-02***	46	2	110	2.48E-01	-2.00E-01	IL6;IL10
MAMMARY GLAND DEVELOPMENT PATHWAY EMBRYONIC DEVELOPMENT STAGE 1 OF 4	**2.70E-02***	18	1	68	3.39E-01	-2.00E-01	TNF
IRON METABOLISM IN PLACENTA	**2.82E-02***	12	1	19	2.74E-01	1.09E+00	FTH1
EXTRACELLULAR VESICLES IN THE CROSSTALK OF CARDIAC CELLS	**4.08E-02***	21	1	104	3.62E-01	-2.00E-01	IL6
GALANIN RECEPTOR PATHWAY	**4.35E-02***	21	1	134	3.62E-01	-2.00E-01	IL6

8. Enrichment results for PD on KEGG database

8.1. Top KEGG pathways predicted with GNNenrich on the Parkinson list for $\beta = 1$.

Table 8. Top 20 KEGG pathways predicted with GNNenrich on the Parkinson list for $\beta = 1$ sorted according to BH first and then according to g:Profiler p-values. In this case, there's no common pathway with the other two methods. Only one pathway is significant with GNNenrich.

Pathway Name	BH	Size	Overlap	PPI	g:Profiler	EnrichNet	Gene in the overlap
KEGG_PANCREATIC_CANCER	**3.70E-03****	70	0	288	NA	-1.24E-01	
KEGG_HEMATOPOIETIC_CELL_LINEAGE	**7.03E-02**	87	4	153	1.43E-01	1.76E-01	IL1A;IL6;TNF;IL1B
KEGG_PROSTATE_CANCER	**1.08E-01**	89	2	374	6.38E-01	1.66E-02	GSK3B;GSTP1
KEGG_ERBB_SIGNALING_PATHWAY	**1.08E-01**	87	1	327	8.07E-01	3.11E-02	GSK3B
KEGG_REGULATION_OF_ACTIN_CYTOSKELETON	**1.08E-01**	213	1	403	9.56E-01	-6.11E-02	FGF20
KEGG_MAPK_SIGNALING_PATHWAY	**1.25E-01**	267	7	726	2.57E-01	1.18E-01	CACNA1A;FGF20;TNF;BDNF; IL1B;MAPT;IL1A
KEGG_MELANOMA	**1.30E-01**	71	1	258	8.01E-01	3.96E-02	FGF20
KEGG_CHEMOKINE_SIGNALING_PATHWAY	**1.61E-01**	189	1	765	9.56E-01	-5.15E-02	GSK3B
KEGG_GLIOMA	**1.61E-01**	65	0	286	NA	-1.24E-01	
KEGG_CHRONIC_MYELOID_LEUKEMIA	**1.74E-01**	73	0	259	NA	-1.24E-01	
KEGG_TYPE_II_DIABETES_MELLITUS	**2.00E-01**	47	2	158	3.47E-01	1.49E-01	CACNA1A;TNF
KEGG_BASAL_CELL_CARCINOMA	**2.00E-01**	55	1	101	7.89E-01	7.16E-02	GSK3B
KEGG_NON_SMALL_CELL_LUNG_CANCER	**2.00E-01**	54	0	208	NA	-1.24E-01	
KEGG_CYTOKINE_CYTOKINE_RECEPTOR_INTERACTION	**2.26E-01**	265	5	683	5.91E-01	-2.68E-02	IL6;TNF;IL10;IL1B;IL1A
KEGG_COMPLEMENT_AND_COAGULATION_CASCADES	**2.26E-01**	69	1	143	8.01E-01	3.38E-02	A2M
KEGG_VASOPRESSIN_REGULATED_WATER_REABSORPTION	**2.26E-01**	44	0	96	NA	-1.24E-01	
KEGG_CELL_ADHESION_MOLECULES_CAMS	**2.26E-01**	133	0	94	NA	-1.24E-01	
KEGG_MATURITY_ONSET_DIABETES_OF_THE_YOUNG	**2.26E-01**	25	0	29	NA	-1.24E-01	
KEGG_PPAR_SIGNALING_PATHWAY	**2.47E-01**	69	0	104	NA	-1.24E-01	
KEGG_NEUROACTIVE_LIGAND_RECEPTOR_INTERACTION	**2.76E-01**	272	10	802	3.09E-02	2.01E-01	DRD4;DRD2;HTR2A;CCKBR; DRD3;GRIN2B;DRD5;CHRNA4; ADRA2A;CCKAR

8.2. Top KEGG pathways predicted with GNNenrich on the Parkinson list for $\beta = 10$.

Table 9. Top 20 KEGG pathways predicted with GNNenrich on the Parkinson list for $\beta = 10$ sorted with BH and g:Profiler scores.

Pathway Name	BH	Size	Overlap	PPI	g:Profiler	EnrichNet	Gene in the overlap
KEGG_PARKINSONS_DISEASE	**1.67E-04*** **	130	18	423	**8.96E-12**	7.54E-01	LRRK2;MT-ND4;NDUFV2; TH;MT-ND1;UCHL1; MT-ND5;SNCA;MT-CO2; SNCAIP;MT-CYB;SLC6A3; MT-CO1;PINK1;MT-ND2; HTRA2;PARK7;MT-ND3
KEGG_METABOLISM_OF_XENOBIOTICS_BY _CYTOCHROME_P450	**1.67E-04*** **	70	11	480	**6.19E-09**	**1.05E+00**	GSTM1;CYP2E1;GSTT1; GSTO2;GSTM3;CYP1A2; GSTZ1;GSTO1;GSTP1; CYP1B1;EPHX1;CYP1A1
KEGG_DRUG_METABOLISM_CYTOCHROME_P450	**1.67E-04*** **	72	11	463	**6.19E-09**	**1.26E+00**	GSTM1;CYP2E1;GSTT1; MAOB;GSTM3;CYP1A2; GSTZ1;CYP2D6;GSTO1; GSTP1;GSTO2;MAOA
KEGG_ALZHEIMERS_DISEASE	**1.67E-04*** **	166	12	547	**6.35E-05**	3.67E-01	TNF;SNCA;MT-CO2; MAPT;MT-CYB;IL1B;APOE; NOS1;MT-CO1;GSK3B; NDUFV2;GRIN2B
KEGG_GLUTATHIONE_METABOLISM	**1.67E-04*** **	50	6	269	**9.11E-05**	**1.33E+00**	GSTM1;GSTT1;GSTM3; GSTZ1;GSTO1;GSTP1;GSTO2
KEGG_TYROSINE_METABOLISM	**1.67E-04*** **	42	6	117	**3.29E-04**	**1.05E+00**	TH;MAOB;MAOA;COMT;GSTZ1;DBH
KEGG_OXIDATIVE_PHOSPHORYLATION	**1.67E-04*** **	132	9	357	**1.10E-03**	-2.18E-02	NDUFV2;MT-ND4;MT-ND1; MT-ND5;MT-CO2; MT-CYB;MT-CO1;MT-ND2; MT-ND3
KEGG_TRYPTOPHAN_METABOLISM	**1.67E-04*** **	40	5	65	**2.36E-03**	7.33E-01	MAOB;CYP1A2;CYP1B1; MAOA;CYP1A1
KEGG_HUNTINGTONS_DISEASE	**1.67E-04*** **	182	9	537	**9.37E-03**	-4.30E-02	MT-CO1;TFAM;BDNF; MT-CO2;MT-CYB;SOD2; GRIN2B;NDUFV2;TBP
KEGG_NEUROACTIVE_LIGAND _RECEPTOR_INTERACTION	**1.67E-04*** **	272	10	802	**3.09E-02**	2.01E-01	DRD2;CHRNA4;DRD4; ADRA2A;DRD3;DRD5; HTR2A;CCKAR;CCKBR;GRIN2B
KEGG_CALCIUM_SIGNALING_PATHWAY	**1.68E-04*** **	178	8	523	**2.45E-02**	3.01E-01	BST1;NOS3;DRD5; CACNA1A;NOS1;CCKAR; HTR2A;CCKBR
KEGG_GRAFT_VERSUS_HOST_DISEASE	**4.63E-04*** **	41	4	67	**1.93E-02**	6.59E-01	TNF;IL1A;IL1B;IL6
KEGG_HEMATOPOIETIC_CELL_LINEAGE	**9.96E-04*** **	87	4	153	1.43E-01	1.76E-01	TNF;IL1A;IL1B;IL6
KEGG_PRION_DISEASES	**1.59E-03** **	35	4	155	**1.17E-02**	4.97E-01	IL1A;IL1B;IL6;PRNP
KEGG_ARGININE_AND_PROLINE_METABOLISM	**2.47E-03** **	54	4	62	**4.11E-02**	3.76E-01	MAOB;MAOA;NOS1;NOS3
KEGG_LEISHMANIA_INFECTION	**3.01E-03** **	72	4	264	9.66E-02	2.43E-01	TNF;IL10;IL1B;IL1A
KEGG_MAPK_SIGNALING_PATHWAY	**3.81E-03** **	267	7	726	2.57E-01	1.18E-01	FGF20;TNF;MAPT; IL1B;IL1A;BDNF;CACNA1A
KEGG_STEROID_HORMONE_BIOSYNTHESIS	**1.60E-02** *	55	3	160	1.60E-01	1.07E-01	COMT;CYP1B1;CYP1A1
KEGG_TYPE_I_DIABETES_MELLITUS	**1.93E-02** *	43	3	75	1.07E-01	5.96E-01	TNF;IL1A;IL1B
KEGG_TYPE_II_DIABETES_MELLITUS	**1.93E-02** *	47	2	158	3.47E-01	1.49E-01	TNF;CACNA1A

8.3. Top KEGG pathways predicted with GNNenrich on the Parkinson list for $\beta = 40$.

Table 10. Top 20 KEGG pathways predicted with GNNenrich on the Parkinson list for $\beta = 40$. Pathways are sorted according to BH first and then according to g:Profiler p-values.

Pathway Name	BH	Size	Overlap	PPI	g:Profiler	EnrichNet	Gene in the overlap
KEGG_PARKINSONS_DISEASE	**1.85E-04*****	130	18	423	**8.96E-12**	7.54E-01	TH;SNCAIP;MT-ND1; LRRK2;SNCA; PINK1;UCHL1; MT-ND5;MT-CO1; MT-ND2;MT-ND4; HTRA2;MT-ND3; PARK7;SLC6A3; MT-CYB;NDUFV2; MT-CO2
KEGG_METABOLISM_OF _XENOBIOTICS_BY_CYTOCHROME_P450	**1.85E-04*****	70	11	480	**6.19E-09**	**1.05E+00**	GSTO1;GSTM3; CYP1A2;GSTT1; GSTO2;EPHX1; GSTM1;CYP2E1; GSTZ1;GSTP1; CYP1B1;CYP1A1
KEGG_DRUG_METABOLISM_CYTOCHROME_P450	**1.85E-04*****	72	11	463	**6.19E-09**	**1.26E+00**	GSTO1;GSTM3; GSTT1;GSTO2;MAOB; GSTM1;CYP2D6;CYP2E1;GSTZ1; MAOA;GSTP1;CYP1A2
KEGG_ALZHEIMERS_DISEASE	**1.85E-04*****	166	12	547	**6.35E-05**	3.67E-01	APOE;SNCA;MT-CO1;MAPT; MT-CYB;TNF; NOS1;GSK3B; GRIN2B;IL1B; NDUFV2;MT-CO2
KEGG_GLUTATHIONE_METABOLISM	**1.85E-04*****	50	6	269	**9.11E-05**	**1.33E+00**	GSTO1;GSTM3; GSTT1;GSTO2; GSTM1;GSTZ1; GSTP1
KEGG_TYROSINE_METABOLISM	**1.85E-04*****	42	6	117	**3.29E-04**	**1.05E+00**	TH;DBH;COMT; MAOB;GSTZ1; MAOA
KEGG_OXIDATIVE_PHOSPHORYLATION	**1.85E-04*****	132	9	357	**1.10E-03**	-2.18E-02	MT-ND1;MT-ND5;MT-ND2; MT-CO1;MT-ND4;MT-ND3; MT-CYB;NDUFV2;MT-CO2
KEGG_TRYPTOPHAN_METABOLISM	**1.85E-04*****	40	5	65	**2.36E-03**	7.33E-01	CYP1A2;MAOB;MAOA; CYP1B1;CYP1A1
KEGG_HUNTINGTONS_DISEASE	**1.85E-04*****	182	9	537	**9.37E-03**	-4.30E-02	MT-CO1;BDNF; TFAM;TBP;MT-CYB; GRIN2B;NDUFV2; SOD2;MT-CO2
KEGG_NEUROACTIVE_LIGAND_RECEPTOR_INTERACTION	**1.85E-04*****	272	10	802	**3.09E-02**	2.01E-01	HTR2A;DRD4;ADRA2A;DRD2; CHRNA4;DRD3; DRD5;GRIN2B; CCKAR;CCKBR
KEGG_CALCIUM_SIGNALING_PATHWAY	**3.36E-04*****	178	8	523	**2.45E-02**	3.01E-01	HTR2A;NOS3;BST1;NOS1; DRD5;CCKAR;CCKBR; CACNA1A
KEGG_GRAFT_VERSUS_HOST_DISEASE	**4.63E-04*****	41	4	67	**1.93E-02**	6.59E-01	IL1A;IL1B;IL6;TNF
KEGG_PRION_DISEASES	**7.12E-04*****	35	4	155	1.17E-02	4.97E-01	IL1A;IL1B;IL6;PRNP
KEGG_ARGININE_AND_PROLINE_METABOLISM	**2.64E-03****	54	4	62	**4.11E-02**	3.76E-01	NOS1;MAOA;NOS3;MAOB
KEGG_HEMATOPOIETIC_CELL_LINEAGE	**3.70E-03****	87	4	153	1.43E-01	1.76E-01	IL1A;IL1B;IL6;TNF
KEGG_LEISHMANIA_INFECTION	**3.82E-03****	72	4	264	9.66E-02	2.43E-01	IL1B;IL1A;TNF;IL10
KEGG_MAPK_SIGNALING_PATHWAY	**6.09E-03****	267	7	726	2.57E-01	1.18E-01	BDNF;MAPT;TNF; IL1B;IL1A; FGF20;CACNA1A
KEGG_HISTIDINE_METABOLISM	**7.50E-03****	29	3	51	**4.24E-02**	**1.08E+00**	MAOA;MAOB;HNMT
KEGG_TYPE_I_DIABETES_MELLITUS	**1.54E-02***	43	3	75	1.07E-01	5.96E-01	IL1B;IL1A;TNF
KEGG_STEROID_HORMONE_BIOSYNTHESIS	**1.68E-02***	55	3	160	1.60E-01	1.07E-01	COMT;CYP1B1;CYP1A1

8.4. KEGG pathways predicted specifically with GNNenrich on the Parkinson list for $\beta = 10$

According to GNNenrich, the 13 pathways in table 11 are also linked to Parkinson's disease. Among which, we can cite the following examples: "KEGG TOLL LIKE RECEPTOR SIGNALING PATHWAY" [Trotta et al. (2014); Noelker et al. (2013)], "KEGG HYPERTROPHIC CARDIOMYOPATHY HCM" [Murphy et al. (2005); Enciso et al. (2023)], "KEGG SYSTEMIC LUPUS ERYTHEMATOSUS" [Kim et al. (2023); Wantaneeyawong et al. (2022)], "KEGG TYPE II DIABETES MELLITUS" Riederer et al.

(2011); Yang et al. (2017b) and "KEGG INTESTINAL IMMUNE NETWORK FOR IGA PRODUCTION" [Brown et al. (2023); Pellegrini et al. (2018)].

Table 11. Selection of KEGG pathways predicted specifically with GNNenrich for $\beta = 10$ for the PD use case.

Pathway Name	BH	Size	Overlap	PPI	g:Profiler	EnrichNet	Gene in the overlap
KEGG_MAPK_SIGNALING_PATHWAY	**3.81E-03****	267	7	726	2.57E-01	1.18E-01	FGF20;TNF;MAPT;IL1B; IL1A;BDNF;CACNA1A
KEGG_LEISHMANIA_INFECTION	**3.01E-03****	72	4	264	9.66E-02	2.43E-01	TNF;IL10;IL1B;IL1A
KEGG_HEMATOPOIETIC_CELL_LINEAGE	**9.96E-04*****	87	4	153	1.43E-01	1.76E-01	TNF;IL1A;IL1B;IL6
KEGG_TYPE_I_DIABETES_MELLITUS	**1.93E-02***	43	3	75	1.07E-01	5.96E-01	TNF;IL1A;IL1B
KEGG_AMYOTROPHIC_LATERAL_SCLEROSIS_ALS	**3.52E-02***	53	3	166	1.52E-01	-1.24E-01	GRIN2B;TNF;NOS1
KEGG_STEROID_HORMONE_BIOSYNTHESIS	**1.60E-02***	55	3	160	1.60E-01	1.07E-01	COMT;CYP1B1;CYP1A1
KEGG_NOD_LIKE_RECEPTOR_SIGNALING_PATHWAY	**3.11E-02***	62	3	251	2.05E-01	1.26E-01	TNF;IL1B;IL6
KEGG_HYPERTROPHIC_CARDIOMYOPATHY_HCM	**4.69E-02***	83	3	124	3.03E-01	3.66E-02	TNF;ACE;IL6
KEGG_TOLL_LIKE_RECEPTOR_SIGNALING_PATHWAY	**3.81E-02***	102	3	398	4.02E-01	2.59E-02	TNF;IL1B;IL6
KEGG_SYSTEMIC_LUPUS_ERYTHEMATOSUS	**2.57E-02***	139	3	73	5.89E-01	-1.24E-01	TNF;IL10;GRIN2B
KEGG_ALLOGRAFT_REJECTION	**3.37E-02***	37	2	60	2.82E-01	-1.24E-01	TNF;IL10
KEGG_TYPE_II_DIABETES_MELLITUS	**1.93E-02***	47	2	158	3.47E-01	1.49E-01	TNF;CACNA1A
KEGG_INTESTINAL_IMMUNE_NETWORK_FOR_IGA _PRODUCTION	**3.37E-02***	48	2	115	3.47E-01	-1.24E-01	IL10;IL6

8.5. KEGG pathways predicted specifically with GNNenrich on the Parkinson list for $\beta = 40$

Table 12. Selection of KEGG pathways predicted specifically with GNNenrich with $\beta = 40$ for the PD use case.

Pathway Name	BH	Size	Overlap	PPI	g:Profiler	EnrichNet	Gene in the overlap
KEGG_HEMATOPOIETIC_CELL_LINEAGE	**3.70E-03****	87	4	153	1.43E-01	1.76E-01	IL1A;IL1B;IL6;TNF
KEGG_LEISHMANIA_INFECTION	**3.82E-03****	72	4	264	9.66E-02	2.43E-01	IL1B;IL1A;TNF;IL10
KEGG_MAPK_SIGNALING_PATHWAY	**6.09E-03****	267	7	726	2.57E-01	1.18E-01	BDNF;MAPT;TNF;IL1B;IL1A; FGF20;CACNA1A
KEGG_TYPE_I_DIABETES_MELLITUS	**1.54E-02***	43	3	75	1.07E-01	5.96E-01	IL1B;IL1A;TNF
KEGG_STEROID_HORMONE_BIOSYNTHESIS	**1.68E-02***	55	3	160	1.60E-01	1.07E-01	COMT;CYP1B1;CYP1A1
KEGG_SYSTEMIC_LUPUS_ERYTHEMATOSUS	**2.21E-02***	139	3	73	5.89E-01	-1.24E-01	GRIN2B;IL10;TNF
KEGG_AMYOTROPHIC_LATERAL_SCLEROSIS_ALS	**2.87E-02***	53	3	166	1.52E-01	-1.24E-01	GRIN2B;TNF;NOS1
KEGG_NOD_LIKE_RECEPTOR_SIGNALING_PATHWAY	**3.04E-02***	62	3	251	2.05E-01	1.26E-01	IL1B;IL6;TNF
KEGG_PHENYLALANINE_METABOLISM	**3.12E-02***	18	2	51	1.14E-01	6.94E-01	MAOA;MAOB
KEGG_INTESTINAL_IMMUNE _NETWORK_FOR_IGA_PRODUCTION	**3.38E-02***	48	2	115	3.47E-01	-1.24E-01	IL6;IL10
KEGG_TYPE_II_DIABETES_MELLITUS	**3.38E-02***	47	2	158	3.47E-01	1.49E-01	TNF;CACNA1A
KEGG_ALLOGRAFT_REJECTION	**3.48E-02***	37	2	60	2.82E-01	-1.24E-01	IL10;TNF
KEGG_TOLL_LIKE_RECEPTOR_SIGNALING_PATHWAY	**4.45E-02***	102	3	398	4.02E-01	2.59E-02	IL1B;IL6;TNF
KEGG_HYPERTROPHIC_CARDIOMYOPATHY_HCM	**4.45E-02***	83	3	124	3.03E-01	3.66E-02	ACE;IL6;TNF

9. Enrichment results for PD on Reactome

9.1. Top Reactome pathways predicted with GNNenrich on the Parkinson list for $\beta = 1$.

Table 13. Top 20 Reactome pathways predicted with GNNenrich on the Parkinson list for $\beta = 1$. Pathways are sorted according to BH first and then according to g:Profiler p-values.

Pathway Name	BH	Size	Overlap	PPI	g:Profiler	EnrichNet	Gene in the overlap
REACTOME_RUNX1_REGULATES_TRANSCRIPTION _OF_GENES_INVOLVED_IN_WNT_SIGNALING	**3.80E-03****	6	1	15	2.48E-01	-7.90E-02	ESR1
REACTOME_REMOVAL_OF_AMINOTERMINAL _PROPEPTIDES_FROM_GAMMA_CARBOXYLATED_PROTEINS	**3.80E-03****	10	0	18	NA	-7.90E-02	
REACTOME_GAMMA_CARBOXYLATION_TRANSPORT_AND _AMINO_TERMINAL_CLEAVAGE_OF_PROTEINS	**3.80E-03****	11	0	18	NA	-7.90E-02	
REACTOME_RUNX3_REGULATES_WNT_SIGNALING	**3.80E-03****	8	0	26	NA	-7.90E-02	
REACTOME_REGULATION_OF_INSULIN_LIKE _GROWTH_FACTOR_IGF_TRANSPORT_AND _UPTAKE_BY_INSULIN_LIKE_GROWTH _FACTOR_BINDING_PROTEINS_IGFBPS	**1.69E-02***	124	3	494	3.05E-01	2.57E-02	CST3;IL6;APOE
REACTOME_REGULATION_OF_FOXO _TRANSCRIPTIONAL_ACTIVITY_BY_ACETYLATION	**1.69E-02***	10	0	50	NA	-7.90E-02	
REACTOME_VEGF_LIGAND_RECEPTOR_INTERACTIONS	**1.69E-02***	8	0	33	NA	-7.90E-02	
REACTOME_INTERLEUKIN_2_SIGNALING	**1.69E-02***	12	0	30	NA	-7.90E-02	
REACTOME_AMPK_INHIBITS_CHREBP _TRANSCRIPTIONAL_ACTIVATION_ACTIVITY	**2.82E-02***	8	0	13	NA	-7.90E-02	
REACTOME_INTERLEUKIN_1_PROCESSING	**2.92E-02***	9	2	47	**2.85E-02**	**2.92E+00**	IL1A;IL1B
REACTOME_INCRETIN_SYNTHESIS _SECRETION_AND_INACTIVATION	**2.92E-02***	24	0	88	NA	-7.90E-02	
REACTOME_CELL_SURFACE_INTERACTIONS _AT_THE_VASCULAR_WALL	**2.96E-02***	194	0	227	NA	-7.90E-02	
REACTOME_INTERLEUKIN_4_AND _INTERLEUKIN_13_SIGNALING	**3.25E-02***	111	7	508	**8.84E-04**	4.01E-01	IL6;TNF;IL1A;MAOA; HMOX1;IL1B;IL10
REACTOME_FGFR2C_LIGAND_BINDING_AND_ACTIVATION	**3.95E-02***	13	1	28	3.19E-01	9.21E-01	FGF20
REACTOME_ACTIVATION_OF_THE_AP _1_FAMILY_OF_TRANSCRIPTION_FACTORS	**3.95E-02***	10	0	95	NA	-7.90E-02	
REACTOME_SYNTHESIS_SECRETION_AND _INACTIVATION_OF_GLUCAGON_LIKE_PEPTIDE_1_GLP_1	**4.76E-02***	21	0	86	NA	-7.90E-02	
REACTOME_PHOSPHOLIPASE_C_MEDIATED_CASCADE_FGFR1	**4.97E-02***	16	1	36	3.33E-01	6.71E-01	FGF20
REACTOME_SIGNALING_BY_ACTIVATED _POINT_MUTANTS_OF_FGFR1	**5.07E-02**	11	1	26	3.00E-01	1.05E+00	FGF20
REACTOME_ERYTHROPOIETIN_ACTIVATES_STAT5	**5.92E-02**	7	0	20	NA	-7.90E-02	

9.2. Top Reactome pathways predicted with GNNenrich on the Parkinson list for $\beta = 10$.

The 9 significant pathways predicted specifically by GNNenrich for PD on Reactome datbase are listed in table 17 for example "REACTOME SIGNALING BY INTERLEUKINS" [Bonte et al. (2021)], "REACTOME GAMMA CARBOXYLATION TRANSPORT AND AMINO TERMINAL CLEAVAGE OF PROTEINS" [Smeyne and Smeyne (2013); Wang et al. (2020); Niu et al. (2024)] and "REACTOME REMOVAL OF AMINOTERMINAL PROPEPTIDES FROM GAMMA CARBOXYLATED PROTEINS" [Kim and Lee (2020)].

Table 14. Top 20 Reactome pathways predicted with GNNenrich on the Parkinson list for $\beta = 10$. Pathways are sorted according to BH first and then according to g:Profiler p-values. All these pathways are significant with GNNenrich and g:Profiler.

Pathway Name	BH	Size	Overlap	PPI	g:Profiler	EnrichNet	Gene in the overlap
REACTOME_BIOLOGICAL_OXIDATIONS	**8.46E-04***	220	18	624	**1.61E-11**	6.33E-01	EPHX1;MAOB;CYP1B1;CYP2E1; CYP1A1;NAT2; MAOA;GSTP1;MTR;COMT; GSTM1;GSTO2;GSTT1; NQO2;CYP1A2;GSTO1;GSTM3; CYP2D6;GSTZ1
REACTOME_PHASE_II_CONJUGATION_OF_COMPOUNDS	**8.46E-04***	109	10	361	**5.11E-07**	1.11E+00	NAT2;MTR;CYP1A2;COMT;GSTO1; GSTO2;GSTM3; GSTP1;GSTM1;GSTZ1;GSTT1
REACTOME_DOPAMINE_RECEPTORS	**8.46E-04***	5	4	23	**2.78E-06**	**8.92E+00**	DRD3;DRD5;DRD4;DRD2
REACTOME_GLUTATHIONE_CONJUGATION	**8.46E-04***	36	6	227	**2.78E-06**	2.06E+00	GSTO1;GSTO2;GSTM3; GSTP1;GSTM1;GSTZ1;GSTT1
REACTOME_PHASE_I_FUNCTIONALIZATION _OF_COMPOUNDS	**8.46E-04***	104	9	271	**1.97E-05**	3.07E-01	CYP1A1;EPHX1;NQO2; CYP1A2;MAOA;MAOB;CYP2D6; CYP1B1;CYP2E1
REACTOME_RESPIRATORY_ELECTRON_TRANSPORT	**8.46E-04***	103	9	413	**1.97E-05**	4.43E-02	MT-ND2;MT-ND5; MT-ND4;MT-ND3;MT-ND1;MT-CYB; NDUFV2;MT-CO2;MT-CO1
REACTOME_NEUROTRANSMITTER_CLEARANCE	**8.46E-04***	11	4	14	**7.82E-05**	**3.52E+00**	COMT;SLC6A4;MAOA;SLC6A3
REACTOME_RESPIRATORY_ELECTRON_TRANSPORT_ATP _SYNTHESIS_BY_CHEMIOSMOTIC_COUPLING_AND_HEAT _PRODUCTION_BY_UNCOUPLING_PROTEINS	**8.46E-04***	127	9	419	**7.82E-05**	3.35E-02	MT-ND2;MT-ND5;MT-ND4; MT-ND3;MT-ND1;MT-CYB; NDUFV2;MT-CO2;MT-CO1
REACTOME_AMINE_LIGAND_BINDING_RECEPTORS	**8.46E-04***	42	6	149	**7.82E-05**	1.16E+00	ADRA2A;DRD4;DRD2;HTR2A;DRD5 ;DRD3
REACTOME_INTERLEUKIN_10_SIGNALING	**8.46E-04***	46	6	250	**1.11E-04**	7.39E-01	IL1A;IL1RN;IL6;TNF;IL10;IL1B
REACTOME_THE_CITRIC_ACID _TCA_CYCLE_AND_RESPIRATORY _ELECTRON_TRANSPORT	**8.46E-04***	178	10	449	**1.26E-04**	8.17E-02	MT-ND2;MT-ND5;MT-ND4; MT-ND3;MT-ND1;MT-CYB; NDUFV2;MT-CO2;MT-CO1;GSTZ1
REACTOME_METHYLATION	**8.46E-04***	14	4	25	**1.49E-04**	**3.19E+00**	COMT;GSTO1;MTR;CYP1A2
REACTOME_DRUG_ADME	**8.46E-04***	109	7	260	**1.49E-04**	4.28E-01	NAT2;GSTM1;GSTT1;PON1;CYP2D6; GSTP1;ABCB1;CYP2E1
REACTOME_BIOSYNTHESIS_OF _SPECIALIZED_PRORESOLVING _MEDIATORS_SPMS	**8.46E-04***	19	4	86	**4.35E-04**	1.31E+00	CYP1A1;CYP2D6;CYP2E1;CYP1A2
REACTOME_METABOLISM_OF_VITAMINS _AND_COFACTORS	**8.46E-04***	192	9	211	**8.38E-04**	4.75E-01	MTR;MTHFR;GSTO1;GCH1; NOS3;APOE;GSTO2; BST1;PDXK
REACTOME_INTERLEUKIN_4_AND _INTERLEUKIN_13_SIGNALING	**8.46E-04***	111	7	508	**8.84E-04**	4.01E-01	IL1A;HMOX1;IL6;TNF;MAOA; IL10;IL1B
REACTOME_INTERLEUKIN_1_PROCESSING	**8.46E-04***	9	2	47	**2.85E-02**	**2.92E+00**	IL1A;IL1B
REACTOME_AMYLOID_FIBER_FORMATION	**8.46E-04***	110	5	187	**2.86E-02**	8.94E-01	SNCA;SNCAIP;CALB1;CST3;APOE
REACTOME_COMPLEX_I_BIOGENESIS	**8.90E-04***	57	6	321	**2.48E-04**	1.35E-01	MT-ND2;MT-ND5;MT-ND4;MT-ND3; MT-ND1;NDUFV2
REACTOME_CYTOCHROME_P450 _ARRANGED_BY_SUBSTRATE_TYPE	**1.69E-03****	64	5	169	**3.81E-03**	3.21E-01	CYP1A1;CYP1A2;CYP2D6; CYP1B1;CYP2E1

9.3. Top Reactome pathways predicted with GNNenrich on the Parkinson list for $\beta = 40$.

Table 15. Top 20 Reactome pathways predicted with GNNenrich on the Parkinson list for $\beta = 40$. Pathways are sorted according to BH first and then according to g:Profiler p-values. All these pathways are significant with GNNenrich and g:Profiler.

Pathway Name	BH	Size	Overlap	PPI	g:Profiler	EnrichNet	Gene in the overlap
REACTOME_BIOLOGICAL_OXIDATIONS	**7.05E-04*****	220	18	624	**1.61E-11**	6.33E-01	MTR;GSTO1;CYP2E1;GSTM3;COMT; NAT2;CYP1B1;GSTT1;GSTM1;CYP1A1; GSTO2;EPHX1;MAOB;NQO2; MAOA;CYP1A2; GSTZ1;CYP2D6;GSTP1
REACTOME_PHASE_II_CONJUGATION_OF_COMPOUNDS	**7.05E-04*****	109	10	361	**5.11E-07**	1.11E+00	NAT2;MTR;CYP1A2;GSTM3;GSTT1; GSTM1;GSTZ1;GSTO1; GSTO2;COMT;GSTP1
REACTOME_DOPAMINE_RECEPTORS	**7.05E-04*****	5	4	23	**2.78E-06**	**8.92E+00**	DRD5;DRD4;DRD3;DRD2
REACTOME_GLUTATHIONE_CONJUGATION	**7.05E-04*****	36	6	227	**2.78E-06**	2.06E+00	GSTM3;GSTT1;GSTM1;GSTZ1; GSTO1;GSTO2;GSTP1
REACTOME_PHASE_I_FUNCTIONALIZATION_OF_COMPOUNDS	**7.05E-04*****	104	9	271	**1.97E-05**	3.07E-01	MAOB;NQO2;MAOA;CYP1A2; CYP1B1;CYP1A1;CYP2E1; CYP2D6;EPHX1
REACTOME_RESPIRATORY _ELECTRON_TRANSPORT	**7.05E-04*****	103	9	413	**1.97E-05**	4.43E-02	MT-ND4;MT-CO1;MT-ND3;MT-ND1; MT-ND2;MT-ND5;MT-CO2; NDUFV2;MT-CYB
REACTOME_NEUROTRANSMITTER_CLEARANCE	**7.05E-04*****	11	4	14	**7.82E-05**	**3.52E+00**	SLC6A3;SLC6A4;COMT;MAOA
REACTOME_RESPIRATORY_ELECTRON _TRANSPORT_ATP_SYNTHESIS_BY_CHEMIOSMOTIC _COUPLING_AND_HEAT_PRODUCTION _BY_UNCOUPLING_PROTEINS	**7.05E-04*****	127	9	419	**7.82E-05**	3.35E-02	MT-ND4;MT-CO1;MT-ND3;MT-ND1; MT-ND2;MT-ND5;MT-CO2;NDUFV2; MT-CYB
REACTOME_AMINE_LIGAND_BINDING_RECEPTORS	**7.05E-04*****	42	6	149	**7.82E-05**	1.16E+00	DRD4;DRD3;ADRA2A;DRD2; DRD5;HTR2A
REACTOME_INTERLEUKIN_10_SIGNALING	**7.05E-04*****	46	6	250	**1.11E-04**	7.39E-01	IL10;TNF;IL1B;IL1A;IL1RN;IL6
REACTOME_THE_CITRIC_ACID_TCA _CYCLE_AND_RESPIRATORY_ELECTRON_TRANSPORT	**7.05E-04*****	178	10	449	**1.26E-04**	8.17E-02	MT-ND4;MT-CO1;MT-ND3;GSTZ1; MT-ND1;MT-ND2;MT-ND5; MT-CO2;NDUFV2;MT-CYB
REACTOME_METHYLATION	**7.05E-04*****	14	4	25	**1.49E-04**	**3.19E+00**	MTR;CYP1A2;GSTO1;COMT
REACTOME_DOPAMINE_CLEARANCE_FROM _THE_SYNAPTIC_CLEFT	**7.05E-04*****	5	3	8	**1.73E-04**	**6.67E+00**	SLC6A3;COMT;MAOA
REACTOME_BIOSYNTHESIS_OF_MARESIN_LIKE_SPMS	**7.05E-04*****	6	3	40	**3.03E-04**	**3.52E+00**	CYP1A2;CYP2D6;CYP2E1
REACTOME_BIOSYNTHESIS_OF_SPECIALIZED _PRORESOLVING_MEDIATORS_SPMS	**7.05E-04*****	19	4	86	**4.35E-04**	1.31E+00	CYP2E1;CYP2D6;CYP1A2;CYP1A1
REACTOME_BIOSYNTHESIS_OF_MARESINS	**7.05E-04*****	8	3	41	**7.12E-04**	**2.92E+00**	CYP1A2;CYP2D6;CYP2E1
REACTOME_XENOBIOTICS	**7.05E-04*****	23	4	119	**8.29E-04**	8.21E-01	CYP2E1;CYP2D6;CYP1A2;CYP1A1
REACTOME_METABOLISM_OF_VITAMINS_AND_COFACTORS	**7.05E-04*****	192	9	211	**8.38E-04**	4.75E-01	BST1;MTHFR;MTR;GCH1;GSTO1; APOE;GSTO2;PDXK;NOS3
REACTOME_INTERLEUKIN_4_AND _INTERLEUKIN_13_SIGNALING	**7.05E-04*****	111	7	508	**8.84E-04**	4.01E-01	IL10;TNF;MAOA;IL1B; HMOX1;IL1A;IL6
REACTOME_AMYLOID_FIBER_FORMATION	**7.05E-04*****	110	5	187	**2.86E-02**	8.94E-01	SNCAIP;APOE;CST3;SNCA;CALB1

9.4. Reactome pathways predicted specifically with GNNenrich on the Parkinson list for $\beta = 1$

Table 16. Selection of Reactome pathways predicted specifically with GNNenrich with $\beta = 1$ for PD use case.

Pathway Name	BH	Size	Overlap	PPI	g:Profiler	EnrichNet	Gene in the overlap
REACTOME_RUNX1_REGULATES_TRANSCRIPTION _OF_GENES_INVOLVED_IN_WNT_SIGNALING	**3.80E-03****	6	1	15	2.48E-01	-7.90E-02	ESR1
REACTOME_REGULATION_OF_INSULIN _LIKE_GROWTH_FACTOR_IGF_TRANSPORT_AND_UPTAKE _BY_INSULIN_LIKE_GROWTH _FACTOR_BINDING_PROTEINS_IGFBPS	**1.69E-02***	124	3	494	3.05E-01	2.57E-02	CST3;IL6;APOE
REACTOME_FGFR2C_LIGAND_BINDING_AND_ACTIVATION	**3.95E-02***	13	1	28	3.19E-01	9.21E-01	FGF20
REACTOME_PHOSPHOLIPASE_C_MEDIATED_CASCADE_FGFR1	**4.97E-02***	16	1	36	3.33E-01	6.71E-01	FGF20

9.5. Reactome pathways predicted specifically with GNNenrich on the Parkinson list for $\beta = 10$

Table 17. Selection of Reactome pathways predicted specifically with GNNenrich with $\beta = 10$ for PD use case.

Pathway Name	BH	Size	Overlap	PPI	g:Profiler	EnrichNet	Gene in the overlap
REACTOME_SIGNALING_BY_INTERLEUKINS	**2.35E-02***	474	10	1365	7.81E-02	9.58E-02	IL1A;HMOX1;IL1RN;GSTO1; IL6;MAOA;TNF;SOD2;IL10;IL1B
REACTOME_LEISHMANIA_INFECTION	**4.50E-02***	219	5	633	2.29E-01	1.69E-01	IL1A;HMOX1;IL6;IL10;IL1B
REACTOME_INTERLEUKIN_12_SIGNALING	**4.78E-02***	47	3	99	7.48E-02	2.31E-01	GSTO1;IL10;SOD2
REACTOME_INTERLEUKIN_12_FAMILY_SIGNALING	**3.46E-02***	57	3	126	1.07E-01	1.64E-01	GSTO1;IL10;SOD2
REACTOME_BINDING_AND_UPTAKE _OF_LIGANDS_BY_SCAVENGER_RECEPTORS	**2.38E-02***	98	3	89	2.58E-01	7.39E-01	FTH1;APOE;FTL
REACTOME_DEFECTIVE_INTRINSIC_PATHWAY _FOR_APOPTOSIS	**2.35E-02***	25	2	101	1.41E-01	-7.90E-02	CAST;SOD2
REACTOME_RUNX1_REGULATES_ TRANSCRIPTION_OF_GENES_INVOLVED_IN_WNT_SIGNALING	**1.09E-02***	6	1	15	2.48E-01	-7.90E-02	ESR1
REACTOME_REMOVAL_OF_AMINOTERMINAL _PROPEPTIDES_FROM _GAMMA_CARBOXYLATED_PROTEINS	**2.85E-02***	10	0	18	NA	-7.90E-02	
REACTOME_GAMMA_CARBOXYLATION _TRANSPORT_AND_AMI NO_TERMINAL_CLEAVAGE_OF_PROTEINS	**3.26E-02***	11	0	18	NA	-7.90E-02	

9.6. Reactome pathways predicted specifically with GNNenrich on the Parkinson list for $\beta = 40$

Table 18. Selection of Reactome pathways predicted specifically with GNNenrich with $\beta = 40$ for the PD use case.

Pathway Name	BH	Size	Overlap	PPI	g:Profiler	EnrichNet	Gene in the overlap
REACTOME_SIGNALING_BY_INTERLEUKINS	**2.25E-02***	474	10	1365	7.81E-02	9.58E-02	IL10;TNF;MAOA;IL1B; SOD2;HMOX1;IL1A;GSTO1;IL1RN; IL6
REACTOME_BINDING_AND_UPTAKE_OF _LIGANDS_BY_SCAVENGER_RECEPTORS	**3.03E-02***	98	3	89	2.58E-01	7.39E-01	FTL;FTH1;APOE
REACTOME_RUNX1_REGULATES_TRANSCRIPTION _OF_GENES_INVOLVED_IN_WNT_SIGNALING	**3.30E-02***	6	1	15	2.48E-01	-7.90E-02	ESR1
REACTOME_DEFECTIVE_INTRINSIC _PATHWAY_FOR_APOPTOSIS	**3.91E-02***	25	2	101	1.41E-01	-7.90E-02	SOD2;CAST
REACTOME_LEISHMANIA_INFECTION	**4.56E-02***	219	5	633	2.29E-01	1.69E-01	IL10;IL1B;HMOX1;IL1A;IL6
REACTOME_INTERLEUKIN_12_FAMILY_SIGNALING	**4.68E-02***	57	3	126	1.07E-01	1.64E-01	SOD2;GSTO1;IL10
REACTOME_INTERLEUKIN_12_SIGNALING	**4.80E-02***	47	3	99	7.48E-02	2.31E-01	SOD2;GSTO1;IL10
REACTOME_NUCLEAR_RECEPTOR_TRANSCRIPTION _PATHWAY	**4.80E-02***	53	3	130	9.65E-02	4.50E-01	ESR2;NR4A2;ESR1

10. Enrichment results for GC on WikiPathways

10.1. Top Wikipathways pathways predicted with GNNenrich on the Gastric Cancer for $\beta = 1$.

Table 19. Top 20 WikiPathways pathways predicted with GNNenrich on the Gastric Cancer for $\beta = 1$. Pathways are sorted according to BH first and then according to g:Profiler p-values.

Pathway Name	BH	Size	Overlap	PPI	g:Profiler	EnrichNet	Gene in the overlap
EGFR TYROSINE KINASE INHIBITOR RESISTANCE	**2.82E-04*****	84	15	1129	**2.46E-13**	**1.31E+00**	KRAS;JAK1;PIK3CA; PRKCA;PTEN; PDGFRB;ERBB2;BRAF; NF1;HRAS; AXL;EGFR;PDGFRA; FGFR2;NRAS
ERBB SIGNALING PATHWAY	**2.82E-04*****	92	9	1186	**3.72E-06**	5.87E-01	KRAS;PIK3CA; PRKCA;ERBB2; TP53;BRAF;HRAS; EGFR;NRAS
ONCOSTATIN M SIGNALING PATHWAY	**2.82E-04*****	65	7	777	**3.12E-05**	3.94E-01	KRAS;JAK1;PRKCA; MAPK14;JAK3;TP53; HRAS
ANGIOGENESIS	**2.82E-04*****	24	4	312	**5.42E-04**	**1.13E+00**	PIK3CA;FGFR2;MAPK14; PDGFRA
ESTROGEN SIGNALING PATHWAY	**2.82E-04*****	23	3	247	**6.07E-03**	**1.61E+00**	BRAF;PIK3CA;MAPK14
OVERVIEW OF INTERFERONSMEDIATED SIGNALING PATHWAY	**2.82E-04*****	37	3	253	**1.73E-02**	6.62E-01	JAK1;PIK3CA;PRKCA
MAPK PATHWAY IN CONGENITAL THYROID CANCER	**2.82E-04*****	16	2	217	**2.91E-02**	**1.54E+00**	KRAS;BRAF
IL7 SIGNALING PATHWAY	**2.82E-04*****	25	2	338	5.35E-02	3.73E-02	JAK1;JAK3
ULTRACONSERVED REGION 339 MODULATION OF TUMOR SUPPRESSOR MICRORNAS IN CANCER	**2.82E-04*****	5	1	32	9.30E-02	NA	TP53
NEOVASCULARISATION PROCESSES	**2.82E-04*****	37	2	320	9.71E-02	2.57E-01	EPHB4;KIT
SOMATROPH AXIS GH AND ITS RELATIONSHIP TO DIETARY RESTRICTION AND AGING	**2.82E-04*****	6	1	94	1.05E-01	**1.79E+00**	PTEN
TNFRELATED WEAK INDUCER OF APOPTOSIS TWEAK SIGNALING PATHWAY	**2.82E-04*****	42	2	366	1.14E-01	1.37E-01	CTNNB1;MAPK14
INTERLEUKIN11 SIGNALING PATHWAY	**2.82E-04*****	44	2	510	1.20E-01	-1.29E-01	JAK1;HRAS
TYPE III INTERFERON SIGNALING	**2.82E-04*****	10	1	61	1.48E-01	**8.23E-01**	JAK1
LNCRNAMEDIATED MECHANISMS OF THERAPEUTIC RESISTANCE	**2.82E-04*****	11	1	52	1.56E-01	**2.54E+00**	TP53
MIRNAS INVOLVED IN DNA DAMAGE RESPONSE	**2.82E-04*****	68	2	152	2.05E-01	**1.17E+00**	ATM;TP53
MAPK AND NFKB SIGNALING PATHWAYS INHIBITED BY YERSINIA YOPJ	**2.82E-04*****	12	0	107	NA	-4.63E-01	
4HYDROXYTAMOXIFEN DEXAMETHASONE AND RETINOIC ACIDS REGULATION OF P27 EXPRESSION	**2.82E-04*****	18	0	176	NA	-4.63E-01	
OSTEOPONTIN SIGNALING	**2.82E-04*****	13	0	128	NA	-4.63E-01	
SARSCOV2 B117 VARIANT ANTAGONISES INNATE IMMUNE ACTIVATION	**2.82E-04*****	9	0	35	NA	NA	

10.2. Top Wikipathways pathways predicted with GNNenrich on the Gastric Cancer for $\beta = 10$.

For specifically gastric tagged pathways, only GNNenrich and g:Profiler highlighted ”Gastrin signaling pathway” (WP4659) with 11 intersections among 115 genes, and a very large number of PPIs (1265) (table 20). On the other hand, two pathways described in wikipathways ”Gastric cancer network” (WP2361/WP2363) did not appear in this analysis. These pathways are also discarded by g:Profiler and EnrichNet leaving the completeness or accuracy in the pathway description as discussion.

Table 20. Top 20 WikiPathways pathways predicted with GNNenrich on the Gastric cancer list for $\beta = 10$. These pathways have a p-value $p < 10E^{-5}$. Pathways are sorted according to BH first and then according to g:Profiler p-values. All these pathways are significant with GNNenrich and g:Profiler.

Pathway Name	BH	Size	Overlap	PPI	g:Profiler	EnrichNet	Gene in the overlap
GLIOBLASTOMA SIGNALING PATHWAYS	**1.20E-04** ***	83	18	975	**1.95E-17**	**1.60E+00**	KRAS;EGFR;HRAS;CDKN2A; MSH6;RB1;ATM;NRAS; PDGFRA;PRKCA;BRAF;PIK3CA; PDGFRB;PTEN;FGFR2;TP53; NF1;ERBB2
HEAD AND NECK SQUAMOUS CELL CARCINOMA	**1.20E-04** ***	74	16	643	**1.47E-15**	**1.79E+00**	SMAD4;KRAS;EGFR;HRAS; CDKN2A;RB1;NRAS;PRKAA2; CTNNB1;PIK3CA;FGFR2;TGFBR2; STK11;PTEN;TP53;ERBB2
EGFR TYROSINE KINASE INHIBITOR RESISTANCE	**1.20E-04** ***	84	15	1129	**2.46E-13**	**1.31E+00**	KRAS;EGFR;HRAS;NRAS; AXL;PDGFRA;PRKCA;BRAF; JAK1;PIK3CA;PDGFRB;PTEN; FGFR2;NF1;ERBB2
ENDOMETRIAL CANCER	**1.20E-04** ***	63	13	729	**2.20E-12**	**1.74E+00**	KRAS;EGFR;APC;HRAS; CDH1;NRAS;CTNNB1;BRAF; PIK3CA;PTEN;FGFR2;TP53;ERBB2
MALIGNANT PLEURAL MESOTHELIOMA	**1.20E-04** ***	439	25	2554	**2.13E-11**	1.44E-01	MAP4K4;APC;MAP3K9;MINK1; JAK1;PIK3CA;KIT;IL1B;EGFR; ULK2;INSR;RPS6KA3;MAPK14; EPHA2;HRAS;ATM;PRKAA2; FGFR2;CDKN2A;CDH1;CTNNB1; PDGFRB;PTEN;TP53;PDGFRA
MELANOMA	**1.20E-04** ***	68	12	750	**1.15E-10**	**1.30E+00**	KRAS;HRAS;CDKN2A;CDH1; RB1;NRAS;BRAF;PIK3CA; PTEN;KIT;TP53;NF1
DNA DAMAGE RESPONSE ONLY ATM DEPENDENT	**1.20E-04** ***	110	14	984	**1.29E-10**	**9.22E-01**	SMAD4;KRAS;APC;HRAS; CDKN2A;INSR;ATM;NRAS; CTNNB1;PIK3CA;PTEN;TP53; MLKL;ERBB2
NONSMALL CELL LUNG CANCER	**1.20E-04** ***	72	12	743	**1.57E-10**	**8.96E-01**	KRAS;EGFR;HRAS;CDKN2A; RB1;NRAS;JAK3;PRKCA; BRAF;PIK3CA;TP53;ERBB2
BLADDER CANCER	**1.20E-04** ***	40	10	504	**1.57E-10**	**2.02E+00**	KRAS;EGFR;HRAS;CDKN2A; CDH1;RB1;NRAS;BRAF; TP53;ERBB2
EMBRYONIC STEM CELL PLURIPOTENCY PATHWAYS	**1.20E-04** ***	116	14	991	**1.90E-10**	6.37E-01	SMAD4;EGFR;APC;HRAS; CTNNB1;PDGFRA;BRAF;JAK1; PDGFRB;BMPR1A;PTEN;FGFR2; BMPR1B;BMPR2
EXTRACELLULAR VESICLEMEDIATED SIGNALING IN RECIPIENT CELLS	**1.20E-04** ***	30	9	297	**2.47E-10**	**2.42E+00**	SMAD4;KRAS;EGFR;APC; HRAS;NRAS;CTNNB1;TGFBR2; ERBB2
BREAST CANCER PATHWAY	**1.20E-04** ***	154	14	1256	**7.66E-09**	4.96E-01	KRAS;EGFR;APC;HRAS;RB1; ATM;NRAS;CTNNB1;BRAF; PIK3CA;PTEN;KIT;TP53;ERBB2
PANCREATIC ADENOCARCINOMA PATHWAY	**1.20E-04** ***	89	11	941	**2.59E-08**	**8.61E-01**	SMAD4;KRAS;EGFR;CDKN2A; RB1;BRAF;JAK1;PIK3CA; TGFBR2;TP53;ERBB2
RAC1PAK1P38MMP2 PATHWAY	**1.20E-04** ***	68	10	794	**2.59E-08**	**1.42E+00**	KRAS;EGFR;HRAS;MSH2; TP53;NRAS;CTNNB1;PIK3CA; MAPK14;ERBB2
CHROMOSOMAL AND MICROSATELLITE INSTABILITY IN COLORECTAL CANCER	**1.20E-04** ***	73	10	605	**4.65E-08**	**1.09E+00**	SMAD4;KRAS;APC;MSH2; MSH6;MLH1;CTNNB1;BRAF; TGFBR2;TP53
HIPPOMERLIN SIGNALING DYSREGULATION	**1.20E-04** ***	121	12	612	**4.65E-08**	7.06E-01	KRAS;EGFR;EPHA2;HRAS; CDH1;INSR;NRAS;CTNNB1; PDGFRA;PDGFRB;KIT;FGFR2
HIPPO SIGNALING REGULATION PATHWAYS	**1.20E-04** ***	98	11	665	**5.68E-08**	7.11E-01	EPHA2;EGFR;CDH1;INSR; PRKAA2;CTNNB1;PRKCA; PDGFRA;PDGFRB;KIT;FGFR2
PI3KAKT SIGNALING PATHWAY	**1.20E-04** ***	339	18	2460	**7.43E-08**	8.51E-02	KRAS;EGFR;EPHA2;KIT; HRAS;INSR;NRAS;PRKAA2; JAK3;PDGFRA;PRKCA;JAK1; PIK3CA;PDGFRB;PTEN;STK11; TP53;FGFR2
MAPK SIGNALING PATHWAY	**1.20E-04** ***	247	15	1696	**2.74E-07**	1.27E-01	KRAS;EGFR;MAP4K4;HRAS; RPS6KA3;NRAS;PRKCA;BRAF; MAPK14;PDGFRB;TGFBR2; IL1B;TP53;NF1;FGFR2
GASTRIN SIGNALING PATHWAY	**1.20E-04** ***	115	11	1265	**2.74E-07**	4.00E-01	KRAS;EGFR;HRAS;CDH1; CDKN2A;PRKD2;CTNNB1; PRKCA;PIK3CA;KIT;MAPK14

10.3. Top Wikipathways pathways predicted with GNNenrich on the Gastric Cancer for $\beta = 40$.

Table 21. Top 20 WikiPathways pathways predicted with GNNenrich on the Gastric cancer list for $\beta = 40$. Pathways are sorted according to BH first and then according to g:Profiler p-values. All these pathways are significant with GNNenrich and g:Profiler.

Pathway Name	BH	Size	Overlap	PPI	g:Profiler	EnrichNet	Gene in the overlap
GLIOBLASTOMA SIGNALING PATHWAYS	**1.03E-04** ***	83	18	975	**1.95E-17**	**1.60E+00**	ATM;PTEN;EGFR;BRAF;FGFR2;NRAS;PRKCA;PDGFRB;PDGFRA;KRAS;TP53;RB1;ERBB2;HRAS;PIK3CA;NF1;CDKN2A;MSH6
HEAD AND NECK SQUAMOUS CELL CARCINOMA	**1.03E-04** ***	74	16	643	**1.47E-15**	**1.79E+00**	EGFR;CTNNB1;STK11;PTEN;PRKAA2;NRAS;FGFR2;KRAS;CDKN2A;ERBB2;RB1;TP53;TGFBR2;PIK3CA;HRAS;SMAD4
EGFR TYROSINE KINASE INHIBITOR RESISTANCE	**1.03E-04**	84	15	1129	**2.46E-13**	**1.31E+00**	PTEN;EGFR;BRAF;FGFR2;NRAS;PRKCA;PDGFRB;PDGFRA;KRAS;NF1;ERBB2;PIK3CA;AXL;HRAS;JAK1
ENDOMETRIAL CANCER	**1.03E-04** ***	63	13	729	**2.20E-12**	**1.74E+00**	EGFR;BRAF;CTNNB1;APC;FGFR2;NRAS;PTEN;CDH1;KRAS;TP53;ERBB2;PIK3CA;HRAS
MALIGNANT PLEURAL MESOTHELIOMA	**1.03E-04** ***	439	25	2554	**2.13E-11**	1.44E-01	EGFR;APC;PRKAA2;MAP3K9;IL1B;MAPK14;ATM;RPS6KA3;CTNNB1;PDGFRB;CDKN2A;INSR;MINK1;FGFR2;HRAS;ULK2;KIT;PTEN;CDH1;PDGFRA;MAP4K4;TP53;PIK3CA;EPHA2;JAK1
MELANOMA	**1.03E-04** ***	68	12	750	**1.15E-10**	**1.30E+00**	PTEN;BRAF;NRAS;CDH1;NF1;KRAS;CDKN2A;RB1;TP53;PIK3CA;HRAS;KIT
DNA DAMAGE RESPONSE ONLY ATM DEPENDENT	**1.03E-04** ***	110	14	984	**1.29E-10**	**9.22E-01**	ATM;PTEN;CTNNB1;APC;NRAS;KRAS;TP53;ERBB2;CDKN2A;PIK3CA;MLKL;INSR;HRAS;SMAD4
NONSMALL CELL LUNG CANCER	**1.03E-04** ***	72	12	743	**1.57E-10**	**8.96E-01**	EGFR;BRAF;JAK3;NRAS;PRKCA;KRAS;CDKN2A;ERBB2;RB1;TP53;PIK3CA;HRAS
BLADDER CANCER	**1.03E-04** ***	40	10	504	**1.57E-10**	**2.02E+00**	EGFR;BRAF;NRAS;CDH1;KRAS;CDKN2A;ERBB2;RB1;TP53;HRAS
EMBRYONIC STEM CELL PLURIPOTENCY PATHWAYS	**1.03E-04** ***	116	14	991	**1.90E-10**	6.37E-01	PTEN;CTNNB1;EGFR;APC;FGFR2;BRAF;PDGFRB;BMPR1B;PDGFRA;SMAD4;BMPR2;HRAS;BMPR1A;JAK1
EXTRACELLULAR VESICLEMEDIATED SIGNALING IN RECIPIENT CELLS	**1.03E-04** ***	30	9	297	**2.47E-10**	**2.42E+00**	EGFR;CTNNB1;APC;NRAS;KRAS;ERBB2;TGFBR2;HRAS;SMAD4
BREAST CANCER PATHWAY	**1.03E-04** ***	154	14	1256	**7.66E-09**	4.96E-01	ATM;PTEN;CTNNB1;EGFR;APC;BRAF;NRAS;KRAS;TP53;RB1;ERBB2;PIK3CA;HRAS;KIT
PANCREATIC ADENOCARCINOMA PATHWAY	**1.03E-04** ***	89	11	941	**2.59E-08**	**8.61E-01**	EGFR;BRAF;KRAS;TP53;RB1;ERBB2;CDKN2A;TGFBR2;PIK3CA;SMAD4;JAK1
RAC1PAK1P38MMP2 PATHWAY	**1.03E-04** ***	68	10	794	**2.59E-08**	**1.42E+00**	EGFR;CTNNB1;NRAS;TP53;KRAS;ERBB2;MSH2;PIK3CA;MAPK14;HRAS
CHROMOSOMAL AND MICROSATELLITE INSTABILITY IN COLORECTAL CANCER	**1.03E-04** ***	73	10	605	**4.65E-08**	**1.09E+00**	BRAF;CTNNB1;APC;TP53;KRAS;TGFBR2;MSH2;MSH6;MLH1;SMAD4
HIPPOMERLIN SIGNALING DYSREGULATION	**1.03E-04** ***	121	12	612	**4.65E-08**	7.06E-01	EGFR;CTNNB1;FGFR2;NRAS;PDGFRB;CDH1;PDGFRA;KRAS;INSR;EPHA2;HRAS;KIT
HIPPO SIGNALING REGULATION PATHWAYS	**1.03E-04** ***	98	11	665	**5.68E-08**	7.11E-01	EGFR;CTNNB1;PRKAA2;FGFR2;PRKCA;PDGFRB;CDH1;PDGFRA;INSR;EPHA2;KIT
PI3KAKT SIGNALING PATHWAY	**1.03E-04** ***	339	18	2460	**7.43E-08**	8.51E-02	PTEN;EGFR;STK11;JAK3;FGFR2;NRAS;PRKAA2;PRKCA;PDGFRB;PDGFRA;KRAS;TP53;PIK3CA;JAK1;INSR;EPHA2;HRAS;KIT
GASTRIN SIGNALING PATHWAY	**1.03E-04** ***	115	11	1265	**2.74E-07**	4.00E-01	EGFR;CTNNB1;PRKCA;CDH1;KRAS;CDKN2A;PIK3CA;PRKD2;MAPK14;HRAS;KIT
MAPK SIGNALING PATHWAY	**1.03E-04** ***	247	15	1696	**2.74E-07**	1.27E-01	RPS6KA3;EGFR;BRAF;FGFR2;NRAS;PRKCA;PDGFRB;NF1;MAP4K4;KRAS;TP53;TGFBR2;IL1B;MAPK14;HRAS

10.4. Pathways predicted with GNNenrich on the Gastric Cancer for $\beta = 1$.

Table 22. Selection of WikiPathways pathways predicted specifically with GNNenrich with $\beta = 1$ for the GC use case. Genes overlapping with MalaCards are highlighted in blue when the overlap with the query is empty.

Pathway Name	BH	Size	Overlap	PPI	g:Profiler	EnrichNet	Gene in the overlap
MAPK AND NFKB SIGNALING PATHWAYS INHIBITED BY YERSINIA YOPJ	**2.82E-04*****	12	0	107	NA	-4.63E-01	['CHUK', 'MAPK1', 'NFKB1', 'NFKBIA', 'RAF1']
TNFRELATED WEAK INDUCER OF APOPTOSIS TWEAK SIGNALING PATHWAY	**2.82E-04*****	42	2	366	1.14E-01	1.37E-01	CTNNB1;MAPK14
4HYDROXYTAMOXIFEN DEXAMETHASONE AND RETINOIC ACIDS REGULATION OF P27 EXPRESSION	**2.82E-04*****	18	0	176	NA	-4.63E-01	['AKT1', 'EIF4E', 'EIF4EBP1', 'MAP2K1', 'MAP3K11', 'MAPK1', 'MAPK3', 'MTOR', 'RAF1']
OSTEOPONTIN SIGNALING	**2.82E-04*****	13	0	128	NA	-4.63E-01	['CHUK', 'ITGAV', 'ITGB3', 'MAP2K1', 'MAPK1', 'MAPK3', 'MMP9', 'NFKB1', 'PLAU', 'RELA', 'SPP1']
INTERLEUKIN11 SIGNALING PATHWAY	**2.82E-04*****	44	2	510	1.20E-01	-1.29E-01	JAK1;HRAS
IL7 SIGNALING PATHWAY	**2.82E-04*****	25	2	338	5.35E-02	3.73E-02	JAK1;JAK3
SARSCOV2 B117 VARIANT ANTAGONISES INNATE IMMUNE ACTIVATION	**2.82E-04*****	9	0	35	NA	NA	
ALTERED GLYCOSYLATION OF MUC1 IN TUMOR MICROENVIRONMENT	**2.82E-04*****	9	0	73	NA	-4.63E-01	['CHUK', 'IL6', 'MUC1', 'NFKB1', 'NFKBIA', 'RELA', 'TNF']
EPO RECEPTOR SIGNALING	**2.82E-04*****	26	0	390	NA	-4.63E-01	['AKT1', 'GRB2', 'JAK2', 'MAP2K1', 'MAPK1', 'MAPK3', 'PIK3CG', 'PTPRC', 'RAF1', 'SOCS1', 'SRC', 'STAT1', 'STAT3', 'STAT5A']
NEOVASCULARISATION PROCESSES	**2.82E-04*****	37	2	320	9.71E-02	2.57E-01	EPHB4;KIT
NODLIKE RECEPTOR NLR SIGNALING PATHWAY	**2.82E-04*****	9	0	73	NA	-4.63E-01	['CD40', 'CHUK', 'EPHB2', 'MAPK8', 'RELA']
ULTRACONSERVED REGION 339 MODULATION OF TUMOR SUPPRESSOR MICRORNAS IN CANCER	**2.82E-04*****	5	1	32	9.30E-02	NA	TP53

10.5. Pathways predicted with GNNenrich on the Gastric Cancer for $\beta = 10$.

For gastric cancer, 23 pathways linked to inflammatory, cell proliferation and tumorigenesis are specifically identified by GNNenrich (Table 23). As example, we can cite "WNT Signaling" [Zhan et al. (2017)], "LDL Influence on CD14 and TLR4" [de Oliveira and Silva (2012); Fan et al. (2014)], "Ultraconserved Region 339 Modulation of Tumor Suppressor MicroRNAs in Cancer", "Nodlike Receptor NLR Signaling Pathway" [Zhou et al. (2023)] and "MAPK and NF-kB Signaling Pathways Inhibited by Yersinia YopJ" [Nandi and Aroeti (2023)]. "EPO receptor signaling" (WP581, 27 genes) related to cancer angiogenesis will be significantly detected (BH=0.04) with GNNenrich within the GC use case [Sereno et al. (2009); Leibold et al. (2024)]. Although there is no overlap with this list, 390 edges connect the PPI graph of WP581 and the genes of the GC (2a). Similarly the pathway "Altered glycosylation of MUC1 in tumor microenvironment" (WP4480, 9 genes), showing 73 PPIs are only significantly detected (BH = 0.03) by GNNenrich. Primarily, driven by the IL6 gene (Interleukin-6), which interacts with 18 genes from the list of GC [Ashizawa et al. (2005)].

10.6. Pathways predicted with GNNenrich on the Gastric Cancer for $\beta = 40$.

Table 23. Selection of WikiPathways pathways predicted specifically with GNNenrich with $\beta = 10$ for the GC use case. Genes overlapping with MalaCards are highlighted in blue when the overlap with the query is empty.

Pathway Name	BH	Size	Overlap	PPI	g:Profiler	EnrichNet	Gene in the overlap
LDL INFLUENCE ON CD14 AND TLR4	**4.11E-02***	24	2	182	5.03E-02	5.37E-01	IL1B;MAPK14
WNT SIGNALING	**3.89E-02***	114	4	488	5.54E-02	-1.37E-01	CTNNB1;PRKCA;APC;ROCK2
ULTRACONSERVED REGION 339 MODULATION OF TUMOR SUPPRESSOR MICRORNAS IN CANCER	**3.33E-04*****	5	1	32	9.30E-02	NA	TP53
NODLIKE RECEPTOR NLR SIGNALING PATHWAY	**5.94E-04*****	9	0	73	NA	-4.63E-01	['CD40', 'CHUK', 'EPHB2', 'MAPK8', 'RELA']
MAPK AND NFKB SIGNALING PATHWAYS INHIBITED BY YERSINIA YOPJ	**6.75E-03****	12	0	107	NA	-4.63E-01	['CHUK', 'MAPK1', 'NFKB1', 'NFKBIA', 'RAF1']
4HYDROXYTAMOXIFEN DEXAMETHASONE AND RETINOIC ACIDS REGULATION OF P27 EXPRESSION	**2.17E-02***	18	0	176	NA	-4.63E-01	['AKT1', 'EIF4E', 'EIF4EBP1', 'MAP2K1', 'MAP3K11', 'MAPK1', 'MAPK3', 'MTOR', 'RAF1']
OSTEOPONTIN SIGNALING	**2.66E-02***	13	0	128	NA	-4.63E-01	['CHUK', 'ITGAV', 'ITGB3', 'MAP2K1', 'MAPK1', 'MAPK3', 'MMP9', 'NFKB1', 'PLAU', 'RELA', 'SPP1']
NANOPARTICLE TRIGGERED REGULATED NECROSIS	**2.97E-02***	12	0	55	NA	-4.63E-01	['CASP8', 'FADD', 'MAPK8', 'PARP1', 'TNF']
ALTERED GLYCOSYLATION OF MUC1 IN TUMOR MICROENVIRONMENT	**3.01E-02***	9	0	73	NA	-4.63E-01	['CHUK', 'IL6', 'MUC1', 'NFKB1', 'NFKBIA', 'RELA', 'TNF']
EPO RECEPTOR SIGNALING	**4.31E-02***	26	0	390	NA	-4.63E-01	['AKT1', 'GRB2', 'JAK2', 'MAP2K1', 'MAPK1', 'MAPK3', 'PIK3CG', 'PTPRC', 'RAF1', 'SOCS1', 'SRC', 'STAT1', 'STAT3', 'STAT5A']
PROSURVIVAL SIGNALING OF NEUROPROTECTIN D1	**5.00E-02***	15	0	81	NA	-4.63E-01	['BAX', 'BIRC3', 'CASP3', 'CASP8', 'CASP9', 'CYCS', 'FADD', 'TNF', 'TRAF2']
SARSCOV2 B117 VARIANT ANTAGONISES INNATE IMMUNE ACTIVATION	**6.52E-03****	9	0	35	NA	NA	

Table 24. Selection of WikiPathways pathways predicted specifically with GNNenrich with $\beta = 40$ for the GC use case.

Pathway Name	BH	Size	Overlap	PPI	g:Profiler	EnrichNet	Gene in the overlap
IL7 SIGNALING PATHWAY	**2.94E-03****	25	2	338	5.35E-02	3.73E-02	JAK3;JAK1
HOSTPATHOGEN INTERACTION OF HUMAN CORONAVIRUSES INTERFERON INDUCTION	**6.60E-03****	33	2	206	8.13E-02	4.85E-01	MAPK14;JAK1
NEOVASCULARISATION PROCESSES	**7.84E-03****	37	2	320	9.71E-02	2.57E-01	EPHB4;KIT
IL5 SIGNALING PATHWAY	**8.70E-03****	40	2	527	1.06E-01	2.87E-01	KRAS;JAK1
TNFRELATED WEAK INDUCER OF APOPTOSIS TWEAK SIGNALING PATHWAY	**1.02E-02***	42	2	366	1.14E-01	1.37E-01	MAPK14;CTNNB1
IL6 SIGNALING PATHWAY	**1.03E-02***	43	2	504	1.16E-01	2.04E-01	IRF1;JAK1
INTERLEUKIN11 SIGNALING PATHWAY	**1.15E-02***	44	2	510	1.20E-01	-1.29E-01	HRAS;JAK1
ANGIOTENSIN II RECEPTOR TYPE 1 PATHWAY	**1.25E-02***	28	2	171	6.26E-02	5.96E-01	SMAD4;TGFBR2
RESOLVIN E1 AND RESOLVIN D1 SIGNALING PATHWAYS PROMOTING INFLAMMATION RESOLUTION	**1.40E-02***	12	1	125	1.68E-01	6.62E-01	PIK3CA
BURN WOUND HEALING	**1.46E-02***	113	4	465	5.42E-02	-9.29E-02	TP53;PDGFRB;IL1B;FGFR2
WNT SIGNALING	**1.76E-02***	114	4	488	5.54E-02	-1.37E-01	ROCK2;PRKCA;CTNNB1;APC
LDL INFLUENCE ON CD14 AND TLR4	**2.22E-02***	24	2	182	5.03E-02	5.37E-01	MAPK14;IL1B
INTERFERON TYPE I SIGNALING PATHWAYS	**2.22E-02***	54	2	550	1.54E-01	3.73E-02	MAPK14;JAK1
PRADERWILLI AND ANGELMAN SYNDROME	**2.26E-02***	68	3	211	6.15E-02	-2.37E-02	TP53;CDKN2A;RB1
CIRCADIAN RHYTHM GENES	**2.70E-02***	201	5	558	9.33E-02	-1.63E-01	PTEN;PRKAA2;TP53;ROCK2;NTRK3
MIRNA ROLE IN IMMUNE RESPONSE IN SEPSIS	**2.85E-02***	65	2	215	1.94E-01	3.94E-01	MAPK14;IRF1
TH17 CELL DIFFERENTIATION PATHWAY	**2.90E-02***	69	3	490	6.26E-02	5.65E-02	MAPK14;IL1B;JAK1
ECTODERM DIFFERENTIATION	**3.16E-02***	142	4	308	9.75E-02	-1.17E-01	SMAD4;CTNNB1;BMPR1A;FGFR2
WNTBETACATENIN SIGNALING PATHWAY IN LEUKEMIA	**3.25E-02***	26	2	145	5.66E-02	3.94E-01	CTNNB1;APC
MALE INFERTILITY	**3.27E-02***	145	4	266	1.02E-01	-1.59E-01	BRDT;INSR;MLH1;ATM

11. Enrichment results for GC on KEGG

11.1. Top KEGG pathways predicted with GNNenrich on the Gastric cancer list for $\beta = 1$

Table 25. Top 20 KEGG pathways predicted with GNNenrich on the Gastric cancer list for $\beta = 1$. Pathways are sorted according to BH first and then according to g:Profiler p-values.

Pathway Name	BH	Size	Overlap	PPI	g:Profiler	EnrichNet	Gene in the overlap
KEGG_GLIOMA	**5.55E-04*****	65	13	885	**5.31E-12**	**1.43E+00**	PDGFRB;TP53;KRAS;PIK3CA; PTEN;CDKN2A;HRAS;NRAS; BRAF;PRKCA;PDGFRA;RB1;EGFR
KEGG_PANCREATIC_CANCER	**5.55E-04*****	70	11	809	**2.42E-09**	**1.36E+00**	TP53;ERBB2;KRAS;PIK3CA; CDKN2A;TGFBR2;SMAD4;BRAF; RB1;EGFR;JAK1
KEGG_ERBB_SIGNALING_PATHWAY	**5.55E-04*****	87	8	1128	**3.09E-05**	5.63E-01	KRAS;ERBB2;PIK3CA;HRAS; NRAS;BRAF;PRKCA;EGFR
KEGG_BASAL_CELL_CARCINOMA	**9.25E-04*****	55	3	198	**4.43E-02**	2.19E-01	TP53;APC;CTNNB1
KEGG_COLORECTAL_CANCER	**1.24E-01**	62	11	666	**6.86E-10**	**1.61E+00**	TP53;KRAS;APC;MLH1; PIK3CA;TGFBR2;SMAD4;BRAF; MSH2;MSH6;CTNNB1
KEGG_CHRONIC_MYELOID_LEUKEMIA	**1.76E-01**	73	10	921	**6.16E-08**	**8.95E-01**	TP53;KRAS;PIK3CA;CDKN2A; TGFBR2;SMAD4;HRAS;NRAS; BRAF;RB1
KEGG_MELANOMA	**9.01E-01**	71	13	890	**1.12E-11**	**1.43E+00**	PDGFRB;TP53;KRAS;PIK3CA; PTEN;CDKN2A;HRAS;NRAS; BRAF;PDGFRA;RB1;EGFR;CDH1
KEGG_PATHWAYS_IN_CANCER	**9.99E-01**	325	25	2393	**2.46E-13**	4.16E-01	PDGFRB;MLH1;MSH2;KIT; PDGFRA;RB1;EGFR;TP53;PTEN; NRAS;BRAF;CTNNB1;KRAS; APC;ERBB2;PIK3CA;TGFBR2; CDKN2A;SMAD4;HRAS;PRKCA; MSH6;CDH1;FGFR2;JAK1
KEGG_ENDOMETRIAL_CANCER	**9.99E-01**	52	13	653	**3.44E-13**	**2.55E+00**	TP53;ERBB2;APC;KRAS;MLH1; PTEN;PIK3CA;HRAS;NRAS; BRAF;EGFR;CTNNB1;CDH1
KEGG_PROSTATE_CANCER	**9.99E-01**	89	14	970	**1.12E-11**	**1.32E+00**	PDGFRB;KRAS;TP53;ERBB2; PIK3CA;PTEN;HRAS;NRAS;BRAF; PDGFRA;RB1;EGFR;CTNNB1; FGFR2
KEGG_NON_SMALL_CELL_LUNG_CANCER	**9.99E-01**	54	11	649	**1.85E-10**	**1.48E+00**	TP53;ERBB2;KRAS;PIK3CA; CDKN2A;HRAS;NRAS;BRAF; PRKCA;RB1;EGFR
KEGG_BLADDER_CANCER	**9.99E-01**	42	10	478	**2.75E-10**	**2.03E+00**	TP53;ERBB2;KRAS;CDKN2A; HRAS;NRAS;BRAF;RB1; EGFR;CDH1
KEGG_THYROID_CANCER	**9.99E-01**	29	7	297	**1.95E-07**	**1.88E+00**	TP53;KRAS;HRAS;NRAS;BRAF; CTNNB1;CDH1
KEGG_MAPK_SIGNALING_PATHWAY	**9.99E-01**	267	16	1720	**2.30E-07**	2.13E-01	PDGFRB;KRAS;TP53;IL1B; MAP4K4;RPS6KA3;TGFBR2;NF1; HRAS;NRAS;BRAF;PRKCA; PDGFRA;EGFR;MAPK14; FGFR2
KEGG_AXON_GUIDANCE	**9.99E-01**	129	10	773	**1.13E-05**	3.91E-01	KRAS;ROCK2;EPHA8;EPHB4; HRAS;NRAS;EPHA2;EPHB1; EPHA6;LIMK1
KEGG_REGULATION_OF_ACTIN_CYTOSKELETON	**9.99E-01**	213	12	1417	**2.45E-05**	2.61E-01	PDGFRB;KRAS;APC;ROCK2; PIK3CA;HRAS;NRAS;BRAF; PDGFRA;EGFR;FGFR2;LIMK1
KEGG_NEUROTROPHIN_SIGNALING_PATHWAY	**9.99E-01**	126	9	1392	**5.90E-05**	3.73E-01	TP53;KRAS;RPS6KA3;PIK3CA; HRAS;NRAS;BRAF;MAPK14; NTRK3
KEGG_FOCAL_ADHESION	**9.99E-01**	199	11	1488	**6.49E-05**	2.46E-01	PDGFRB;ERBB2;ROCK2;PIK3CA; PTEN;HRAS;BRAF;PRKCA; PDGFRA;EGFR;CTNNB1
KEGG_ADHERENS_JUNCTION	**9.99E-01**	73	7	653	**7.91E-05**	**9.18E-01**	ERBB2;TGFBR2;SMAD4;INSR; EGFR;CTNNB1;CDH1
KEGG_MTOR_SIGNALING_PATHWAY	**9.99E-01**	52	6	472	**1.07E-04**	7.57E-01	PIK3CA;BRAF;STK11;ULK2; RPS6KA3;PRKAA2

11.2. Top KEGG pathways predicted with GNNenrich on the Gastric cancer list for $\beta = 10$

Table 26. Top 20 KEGG pathways predicted with GNNenrich on the Gastric cancer list for $\beta = 10$. These pathways have a p-value $p < 10E^{-5}$ and $BH = 7.57E^{-05}$. Pathways are sorted according to BH first and according to g:Profiler p-values. All these pathways are significant with GNNenrich and g:Profiler.

Pathway Name	BH	Size	Overlap	PPI	g:Profiler	EnrichNet	Gene in the overlap
KEGG_PATHWAYS_IN_CANCER	**7.57E-05** ***	325	25	2393	**2.46E-13**	4.16E-01	MSH2;BRAF;NRAS;PTEN; TP53;JAK1;KIT;MSH6; KRAS;ERBB2;EGFR;PDGFRA; FGFR2;CDKN2A;APC;TGFBR2; CDH1;PDGFRB;PRKCA; CTNNB1;PIK3CA;SMAD4; RB1;MLH1;HRAS
KEGG_ENDOMETRIAL_CANCER	**7.57E-05** ***	52	13	653	**3.44E-13**	**2.55E+00**	TP53;CTNNB1;PIK3CA;BRAF; MLH1;APC;KRAS;CDH1; ERBB2;EGFR;HRAS;NRAS; PTEN
KEGG_GLIOMA	**7.57E-05** ***	65	13	885	**5.31E-12**	**1.43E+00**	TP53;PDGFRA;PRKCA;PIK3CA; BRAF;CDKN2A;RB1;KRAS; EGFR;HRAS;NRAS;PTEN; PDGFRB
KEGG_MELANOMA	**7.57E-05***	71	13	890	**1.12E-11**	**1.43E+00**	TP53;PDGFRA;PIK3CA;BRAF; CDKN2A;RB1;KRAS;CDH1; EGFR;HRAS;NRAS;PTEN;PDGFRB
KEGG_PROSTATE_CANCER	**7.57E-05***	89	14	970	**1.12E-11**	**1.32E+00**	TP53;PDGFRA;CTNNB1;PIK3CA; BRAF;FGFR2;RB1;KRAS; ERBB2;EGFR;HRAS;NRAS; PTEN;PDGFRB
KEGG_NON_SMALL_CELL_LUNG_CANCER	**7.57E-05** ***	54	11	649	**1.85E-10**	**1.48E+00**	TP53;PRKCA;PIK3CA;BRAF; CDKN2A;RB1;KRAS;ERBB2; EGFR;HRAS;NRAS
KEGG_BLADDER_CANCER	**7.57E-05***	42	10	478	**2.75E-10**	**2.03E+00**	TP53;BRAF;CDKN2A;RB1; KRAS;CDH1;ERBB2;EGFR; HRAS;NRAS
KEGG_COLORECTAL_CANCER	**7.57E-05***	62	11	666	**6.86E-10**	**1.61E+00**	TP53;MSH2;CTNNB1;PIK3CA; BRAF;MSH6;SMAD4;MLH1; APC;TGFBR2;KRAS
KEGG_PANCREATIC_CANCER	**7.57E-05** ***	70	11	809	**2.42E-09**	**1.36E+00**	TP53;JAK1;PIK3CA;BRAF; CDKN2A;SMAD4;RB1;TGFBR2; KRAS;ERBB2;EGFR
KEGG_CHRONIC_MYELOID_LEUKEMIA	**7.57E-05***	73	10	921	**6.16E-08**	**8.95E-01**	TP53;PIK3CA;BRAF;CDKN2A; SMAD4;RB1;TGFBR2;KRAS; HRAS;NRAS
KEGG_THYROID_CANCER	**7.57E-05***	29	7	297	**1.95E-07**	**1.88E+00**	TP53;CTNNB1;BRAF;CDH1; KRAS;HRAS;NRAS
KEGG_MAPK_SIGNALING_PATHWAY	**7.57E-05** ***	267	16	1720	**2.30E-07**	2.13E-01	TP53;PDGFRA;PRKCA;RPS6KA3; BRAF;FGFR2;TGFBR2;KRAS; NF1;MAP4K4;MAPK14;EGFR; HRAS;NRAS;IL1B;PDGFRB
KEGG_AXON_GUIDANCE	**7.57E-05** ***	129	10	773	**1.13E-05**	3.91E-01	EPHA2;LIMK1;EPHB4;KRAS; EPHB1;HRAS;EPHA8;NRAS; ROCK2;EPHA6
KEGG_ACUTE_MYELOID_LEUKEMIA	**7.57E-05** ***	57	7	620	**2.01E-05**	9.18E-01	KIT;PIK3CA;BRAF;KRAS; PIM2;HRAS;NRAS
KEGG_REGULATION_OF_ACTIN_CYTOSKELETON	**7.57E-05** ***	213	12	1417	**2.45E-05**	2.61E-01	PDGFRA;PIK3CA;LIMK1;BRAF; FGFR2;APC;KRAS;EGFR; HRAS;NRAS;ROCK2;PDGFRB
KEGG_ERBB_SIGNALING_PATHWAY	**7.57E-05** ***	87	8	1128	**3.09E-05**	5.63E-01	PRKCA;PIK3CA;BRAF;KRAS; ERBB2;EGFR;HRAS;NRAS
KEGG_NEUROTROPHIN_SIGNALING_PATHWAY	**7.57E-05** ***	126	9	1392	**5.90E-05**	3.73E-01	TP53;PIK3CA;RPS6KA3;BRAF; NTRK3;KRAS;MAPK14; HRAS;NRAS
KEGG_FOCAL_ADHESION	**7.57E-05** ***	199	11	1488	**6.49E-05**	2.46E-01	PRKCA;PDGFRA;CTNNB1;PIK3CA; BRAF;ERBB2;EGFR;HRAS; ROCK2;PTEN;PDGFRB
KEGG_GAP_JUNCTION	**7.57E-05***	90	7	610	**2.68E-04**	3.04E-01	PRKCA;PDGFRA;KRAS;EGFR; HRAS;NRAS;PDGFRB
KEGG_CHEMOKINE_SIGNALING_PATHWAY	**7.57E-05** ***	189	9	1359	**8.91E-04**	-5.15E-03	PIK3CA;GRK6;BRAF;JAK3; KRAS;HRAS;ITK;NRAS;ROCK2

11.3. Top KEGG pathways predicted with GNNenrich on the Gastric cancer list for $\beta = 40$

Table 27. Top 20 KEGG pathways predicted with GNNenrich on the Gastric cancer list for $\beta = 40$. Pathways are sorted according to BH first and then according to g:Profiler p-values. All these pathways are significant with GNNenrich and g:Profiler.

Pathway Name	BH	Size	Overlap	PPI	g:Profiler	EnrichNet	Gene in the overlap
KEGG_PATHWAYS_IN_CANCER	**5.74E-05** ***	325	25	2393	**2.46E-13**	4.16E-01	JAK1;ERBB2;TGFBR2;MLH1;TP53; SMAD4;PIK3CA;PRKCA;BRAF;APC; RB1;PDGFRA;NRAS;HRAS; CTNNB1;MSH2;PTEN;EGFR;FGFR2; KRAS;PDGFRB;CDKN2A;MSH6;CDH1;KIT
KEGG_ENDOMETRIAL_CANCER	**5.74E-05** ***	52	13	653	**3.44E-13**	**2.55E+00**	PIK3CA;BRAF;HRAS;ERBB2;APC; CTNNB1;CDH1;MLH1;EGFR;PTEN; TP53;NRAS;KRAS
KEGG_GLIOMA	**5.74E-05** ***	65	13	885	**5.31E-12**	**1.43E+00**	PIK3CA;BRAF;PRKCA;CDKN2A;HRAS; RB1;EGFR;PDGFRA;PTEN;TP53; NRAS;KRAS;PDGFRB
KEGG_PROSTATE_CANCER	**5.74E-05** ***	89	14	970	**1.12E-11**	1.32E+00	PIK3CA;BRAF;HRAS;ERBB2;CTNNB1; PTEN;EGFR;PDGFRA;RB1;KRAS; NRAS;TP53;FGFR2;PDGFRB
KEGG_MELANOMA	**5.74E-05** ***	71	13	890	**1.12E-11**	**1.43E+00**	PIK3CA;BRAF;CDKN2A;HRAS;CDH1; RB1;EGFR;PDGFRA;PTEN;NRAS; TP53;KRAS;PDGFRB
KEGG_NON_SMALL_CELL_LUNG_CANCER	**5.74E-05** ***	54	11	649	**1.85E-10**	**1.48E+00**	PIK3CA;BRAF;PRKCA;CDKN2A;HRAS; ERBB2;RB1;EGFR;TP53;NRAS;KRAS
KEGG_BLADDER_CANCER	**5.74E-05** ***	42	10	478	**2.75E-10**	**2.03E+00**	CDKN2A;BRAF;HRAS;ERBB2;CDH1; RB1;EGFR;NRAS;TP53;KRAS
KEGG_COLORECTAL_CANCER	**5.74E-05** ***	62	11	666	**6.86E-10**	**1.61E+00**	PIK3CA;BRAF;APC;CTNNB1;MSH2; MSH6;MLH1;TGFBR2;TP53;KRAS; SMAD4
KEGG_PANCREATIC_CANCER	**5.74E-05** ***	70	11	809	**2.42E-09**	**1.36E+00**	JAK1;PIK3CA;BRAF;CDKN2A;ERBB2; TGFBR2;RB1;EGFR;TP53;KRAS; SMAD4
KEGG_CHRONIC_MYELOID_LEUKEMIA	**5.74E-05** ***	73	10	921	**6.16E-08**	**8.95E-01**	PIK3CA;BRAF;CDKN2A;HRAS;TGFBR2; RB1;TP53;NRAS;KRAS;SMAD4
KEGG_THYROID_CANCER	**5.74E-05** ***	29	7	297	**1.95E-07**	**1.88E+00**	BRAF;HRAS;CTNNB1;CDH1; NRAS;TP53;KRAS
KEGG_MAPK_SIGNALING_PATHWAY	**5.74E-05** ***	267	16	1720	**2.30E-07**	2.13E-01	PRKCA;BRAF;IL1B;HRAS;MAP4K4; MAPK14;TGFBR2;EGFR;PDGFRA; RPS6KA3;NRAS;KRAS;TP53; FGFR2;NF1;PDGFRB
KEGG_AXON_GUIDANCE	**5.74E-05** ***	129	10	773	**1.13E-05**	3.91E-01	HRAS;EPHA6;LIMK1;KRAS;NRAS; EPHA8;EPHA2;ROCK2;EPHB1;EPHB4
KEGG_ACUTE_MYELOID_LEUKEMIA	**5.74E-05** ***	57	7	620	**2.01E-05**	**9.18E-01**	PIK3CA;BRAF;HRAS;PIM2; KRAS;NRAS;KIT
KEGG_REGULATION_OF_ACTIN_CYTOSKELETON	**5.74E-05** ***	213	12	1417	**2.45E-05**	2.61E-01	PIK3CA;BRAF;HRAS;APC;LIMK1; EGFR;PDGFRA;FGFR2;NRAS;KRAS; ROCK2;PDGFRB
KEGG_ERBB_SIGNALING_PATHWAY	**5.74E-05** ***	87	8	1128	**3.09E-05**	5.63E-01	PIK3CA;BRAF;PRKCA;HRAS;ERBB2; EGFR;KRAS;NRAS
KEGG_NEUROTROPHIN_SIGNALING_PATHWAY	**5.74E-05** ***	126	9	1392	**5.90E-05**	3.73E-01	PIK3CA;BRAF;HRAS;MAPK14;NTRK3; RPS6KA3;KRAS;NRAS;TP53
KEGG_FOCAL_ADHESION	**5.74E-05** ***	199	11	1488	**6.49E-05**	2.46E-01	PIK3CA;PRKCA;BRAF;HRAS;ERBB2; CTNNB1;PTEN;EGFR;PDGFRA;PDGFRB; ROCK2
KEGG_ADHERENS_JUNCTION	**5.74E-05** ***	73	7	653	**7.91E-05**	**9.18E-01**	ERBB2;CTNNB1;CDH1;TGFBR2;INSR; EGFR;SMAD4
KEGG_MTOR_SIGNALING_PATHWAY	**5.74E-05** ***	52	6	472	**1.07E-04**	**7.57E-01**	PIK3CA;BRAF;PRKAA2;STK11; ULK2;RPS6KA3

11.4. KEGG pathways predicted specifically with GNNenrich on the Gastric cancer list for $\beta = 40$

Table 28. Selection of KEGG pathways predicted specifically with GNNenrich with $\beta = 40$ for the GC use case.

Pathway Name	BH	Size	Overlap	PPI	g:Profiler	EnrichNet	Gene in the overlap
KEGG_CALCIUM_SIGNALING_PATHWAY	**1.84E-02** *	178	5	485	8.94E-02	-8.46E-02	PRKCA;ERBB2;EGFR;PDGFRA;PDGFRB
KEGG_LEISHMANIA_INFECTION	**2.79E-02***	72	3	410	8.41E-02	1.83E-01	MAPK14;JAK1;IL1B
KEGG_DORSO_VENTRAL_AXIS_FORMATION	**3.69E-02***	24	2	186	5.28E-02	6.91E-01	EGFR;KRAS
KEGG_PHOSPHATIDYLINOSITOL_SIGNALING_SYSTEM	**4.69E-02***	76	3	411	9.23E-02	-2.19E-02	PIK3CA;PRKCA;PTEN

12. Enrichment results for GC on Reactome

12.1. Top Reactome pathways predicted with GNNenrich on the Gastric cancer list for $\beta = 1$.

Table 29. Top 20 Reactome pathways predicted with GNNenrich on the Gastric cancer list for $\beta = 1$. Pathways are sorted according to BH first and then according to g:Profiler p-values.

Pathway Name	BH	Size	Overlap	PPI	g:Profiler	EnrichNet	Gene in the overlap
REACTOME_SIGNALLING_TO_RAS	**3.45E-04***	20	4	327	**1.10E-04**	1.74E+00	KRAS;MAPK14;NRAS;HRAS
REACTOME_EPHB_MEDIATED_FORWARD_SIGNALING	**3.45E-04***	42	5	421	**1.10E-04**	8.54E-01	ROCK2;EPHB1;EPHB4;HRAS;LIMK1
REACTOME_ACTIVATED_NTRK2_SIGNALS_THROUGH_RAS	**3.45E-04***	9	3	188	**2.27E-04**	**5.82E+00**	KRAS;NRAS;HRAS
REACTOME_SIGNALING_BY_MODERATE _KINASE_ACTIVITY_BRAF_MUTANTS	**3.45E-04***	45	4	449	**1.43E-03**	6.86E-01	KRAS;NRAS;HRAS;BRAF
REACTOME_SHC_MEDIATED_CASCADE_FGFR1	**3.45E-04***	21	3	312	**2.04E-03**	1.20E+00	KRAS;NRAS;HRAS
REACTOME_LOSS_OF_FUNCTION_OF_SMAD2_3_IN_CANCER	**3.45E-04***	7	2	55	**4.44E-03**	**2.39E+00**	SMAD4;TGFBR2
REACTOME_SIGNALING_BY_BRAF_AND_RAF1_FUSIONS	**3.45E-04***	65	4	536	**4.71E-03**	4.02E-01	KRAS;NRAS;HRAS;BRAF
REACTOME_INTERLEUKIN_9_SIGNALING	**3.45E-04***	9	2	100	**7.09E-03**	9.40E-01	JAK1;JAK3
REACTOME_INTERLEUKIN_21_SIGNALING	**3.45E-04***	10	2	106	**8.52E-03**	9.40E-01	JAK1;JAK3
REACTOME_INTERLEUKIN_2_SIGNALING	**3.45E-04***	12	2	150	**1.18E-02**	9.40E-01	JAK1;JAK3
REACTOME_FCERI_MEDIATED_MAPK_ACTIVATION	**3.45E-04***	87	3	440	6.24E-02	7.63E-01	KRAS;HRAS;NRAS
REACTOME_SIGNALLING_TO_P38_VIA_RIT_AND_RIN	**3.45E-04***	5	1	50	8.63E-02	**2.07E+00**	BRAF
REACTOME_NEGATIVE_FEEDBACK_REGULATION _OF_MAPK_PATHWAY	**3.45E-04***	6	1	97	9.89E-02	1.32E+00	BRAF
REACTOME_DEFECTIVE_RIPK1_MEDIATED _REGULATED_NECROSIS	**3.45E-04***	7	1	34	1.09E-01	1.10E+00	MLKL
REACTOME_IFNG_SIGNALING_ACTIVATES_MAPKS	**3.45E-04***	8	1	102	1.19E-01	1.10E+00	JAK1
REACTOME_MAPK1_ERK2_ACTIVATION	**3.45E-04***	9	1	126	1.26E-01	1.62E+00	JAK1
REACTOME_MAPK3_ERK1_ACTIVATION	**3.45E-04***	10	1	139	1.34E-01	1.62E+00	JAK1
REACTOME_NF_KB_ACTIVATION_THROUGH _FADD_RIP_1_PATHWAY _MEDIATED_BY_CASPASE_8_AND_10	**3.45E-04***	13	1	49	1.60E-01	8.15E-01	CASP10
REACTOME_ABERRANT_REGULATION_OF _MITOTIC_G1_S_TRANSITION_IN _CANCER_DUE_TO_RB1_DEFECTS	**3.45E-04***	17	1	96	1.86E-01	5.08E-01	RB1
REACTOME_RAF_INDEPENDENT_MAPK1_3_ACTIVATION	**3.45E-04***	23	1	195	2.23E-01	3.78E-01	JAK1

12.2. Top Reactome pathways predicted with GNNenrich on the Gastric cancer list for $\beta = 10$.

Table 30. Top 20 Reactome pathways predicted with GNNenrich on the Gastric cancer list for $\beta = 10$. These pathways have a p-value $p < 10E^{-5}$ and $BH = 2.38E^{-04}$. Pathways are sorted according to BH first and then according to g:Profiler p-values. All these pathways are significant with GNNenrich and g:Profiler.

Pathway Name	BH	Size	Overlap	PPI	g:Profiler	EnrichNet	Gene in the overlap
REACTOME_SIGNALING BY_RECEPTOR_TYROSINE_KINASES	**2.38E-04***	524	20	3329	**1.32E-07**	1.92E-01	FGFR2;KIT; PIK3CA;KRAS; CTNNB1;NTRK3; RPS6KA3;EGFR; NRAS;PDGFRB; INSR;HRAS; PRKCA;AXL; PDGFRA;ROCK2; ERBB2;JAK3; BRAF;MAPK14
REACTOME_DISEASES_OF_SIGNAL_TRANSDUCTION _BY_GROWTH_FACTOR_RECEPTORS_AND _SECOND_MESSENGERS	**2.38E-04***	432	18	2919	**1.32E-07**	2.78E-01	PTEN;KIT;FGFR2;ERBB2; HRAS;BRAF;PIK3CA;NF1; TGFBR2;APC;PDGFRA;FBXW7; KRAS;SMAD4;EGFR;NRAS; CTNNB1;PDGFRB
REACTOME_SIGNALING_BY_ERBB2_ECD_MUTANTS	**2.38E-04***	16	6	309	**1.32E-07**	**4.32E+00**	ERBB2;HRAS;PIK3CA;KRAS; EGFR;NRAS
REACTOME_GASTRIN_CREB_SIGNALLING _PATHWAY_VIA_PKC_AND_MAPK	**2.38E-04***	18	6	248	**1.99E-07**	**2.52E+00**	HRAS;PRKCA;RPS6KA3;KRAS; EGFR;NRAS
REACTOME_EGFR_TRANSACTIVATION_BY_GASTRIN	**2.38E-04***	9	5	155	**1.99E-07**	**5.22E+00**	HRAS;PRKCA;KRAS;EGFR;NRAS
REACTOME_SHC1_EVENTS_IN_ERBB2_SIGNALING	**2.38E-04***	22	6	316	**5.94E-07**	**2.58E+00**	ERBB2;HRAS;PRKCA;KRAS; EGFR;NRAS
REACTOME_SIGNALING_BY_PDGFRA_TRANSMEMBRANE _JUXTAMEMBRANE_AND_KINASE_DOMAIN_MUTANTS	**2.38E-04***	12	5	239	**8.80E-07**	**4.32E+00**	HRAS;PIK3CA;PDGFRA;KRAS;NRAS
REACTOME_EPH_EPHRIN_SIGNALING	**2.38E-04***	92	9	769	**8.87E-07**	6.59E-01	LIMK1;EPHA6;HRAS;EPHB1; EPHB4;EPHA2;EPHA10;EPHA8; ROCK2
REACTOME_SIGNALING_BY_ERBB2_IN_CANCER	**2.38E-04***	26	6	399	**1.20E-06**	**2.46E+00**	ERBB2;HRAS;PIK3CA;KRAS; EGFR;NRAS
REACTOME_SIGNALING_BY_VEGF	**2.38E-04***	106	9	966	**1.87E-06**	7.03E-01	HRAS;PIK3CA;PRKCA;AXL; KRAS;MAPK14;NRAS;CTNNB1; ROCK2
REACTOME_MAPK_FAMILY_SIGNALING_CASCADES	**2.38E-04***	327	14	2537	**1.87E-06**	2.51E-01	FGFR2;KIT;ERBB2;JAK3; HRAS;BRAF;PIK3CA;NF1; JAK1;PDGFRA;KRAS;EGFR; NRAS;PDGFRB
REACTOME_CONSTITUTIVE_SIGNALING_BY_EGFRVIII	**2.38E-04***	15	5	310	**1.87E-06**	**3.42E+00**	HRAS;PIK3CA;KRAS;EGFR;NRAS
REACTOME_SIGNALING_BY_ERBB2	**2.38E-04***	50	7	697	**1.87E-06**	1.48E+00	ERBB2;HRAS;PIK3CA;PRKCA; KRAS;EGFR;NRAS
REACTOME_DOWNSTREAM_SIGNAL_TRANSDUCTION	**2.38E-04***	29	6	465	**1.87E-06**	1.86E+00	HRAS;PIK3CA;PDGFRA;KRAS; NRAS;PDGFRB
REACTOME_GRB2_EVENTS_IN_ERBB2_SIGNALING	**2.38E-04***	16	5	240	**2.22E-06**	**3.42E+00**	ERBB2;HRAS;KRAS;EGFR;NRAS
REACTOME_RAS_SIGNALING_DOWNSTREAM _OF_NF1_LOSS_OF_FUNCTION_VARIANTS	**2.38E-04***	7	4	81	**2.56E-06**	**4.32E+00**	HRAS;NRAS; NF1;KRAS
REACTOME_NEGATIVE_REGULATION_OF _THE_PI3K_AKT_NETWORK	**2.38E-04***	113	9	1216	**2.56E-06**	6.93E-01	PTEN;KIT;FGFR2;INSR;ERBB2; PIK3CA;PDGFRA;EGFR;PDGFRB
REACTOME_SIGNALING_BY_NTRK3_TRKC	**2.38E-04***	17	5	304	**2.60E-06**	**3.09E+00**	HRAS;PIK3CA; KRAS;NRAS; NTRK3
REACTOME_CONSTITUTIVE_SIGNALING_BY _LIGAND_RESPONSIVE_EGFR_CANCER_VARIANTS	**2.38E-04***	19	5	392	**4.36E-06**	**2.82E+00**	HRAS;PIK3CA; KRAS;EGFR; NRAS
REACTOME_ACTIVATED_NTRK3_SIGNALS_THROUGH_RAS	**2.38E-04***	8	4	163	**4.36E-06**	**8.82E+00**	HRAS;NRAS ;KRAS;NTRK3

12.3. Top Reactome pathways predicted with GNNenrich on the Gastric cancer list for $\beta = 40$.

Table 31. Top 20 Reactome pathways predicted with GNNenrich on the Gastric cancer list for $\beta = 40$. Pathways are sorted according to BH first and then according to g:Profiler p-values. All these pathways are significant with GNNenrich and g:Profiler.

Pathway Name	BH	Size	Overlap	PPI	g:Profiler	EnrichNet	Gene in the overlap
REACTOME_SIGNALING_BY_RECEPTOR _TYROSINE_KINASES	**2.01E-04*****	524	20	3329	**1.32E-07**	1.92E-01	PRKCA;RPS6KA3;CTNNB1;KIT; AXL;PIK3CA;KRAS;MAPK14; ERBB2;FGFR2;JAK3;BRAF; ROCK2;NRAS;PDGFRB;HRAS; INSR;PDGFRA;EGFR;NTRK3
REACTOME_SIGNALING_BY_ERBB2_ECD_MUTANTS	**2.01E-04*****	16	6	309	**1.32E-07**	**4.32E+00**	EGFR;KRAS;ERBB2; NRAS;PIK3CA;HRAS
REACTOME_DISEASES_OF_SIGNAL _TRANSDUCTION_BY_GROWTH_FACTOR_RECEPTORS _AND_SECOND_MESSENGERS	**2.01E-04*****	432	18	2919	**1.32E-07**	2.78E-01	KRAS;EGFR;BRAF;CTNNB1; ERBB2;TGFBR2;KIT;NF1; PTEN;NRAS;PIK3CA;PDGFRB; APC;HRAS;FGFR2;SMAD4; FBXW7;PDGFRA
REACTOME_GASTRIN_CREB_SIGNALLING _PATHWAY_VIA_PKC_AND_MAPK	**2.01E-04*****	18	6	248	**1.99E-07**	**2.52E+00**	KRAS;PRKCA;EGFR;RPS6KA3; NRAS;HRAS
REACTOME_EGFR_TRANSACTIVATION_BY_GASTRIN	**2.01E-04*****	9	5	155	**1.99E-07**	**5.22E+00**	EGFR;PRKCA;KRAS;NRAS;HRAS
REACTOME_SHC1_EVENTS_IN_ERBB2_SIGNALING	**2.01E-04*****	22	6	316	**5.94E-07**	**2.58E+00**	KRAS;PRKCA;EGFR; ERBB2;NRAS;HRAS
REACTOME_SIGNALING_BY_PDGFRA _TRANSMEMBRANE_JUXTAMEMBRANE _AND_KINASE_DOMAIN_MUTANTS	**2.01E-04*****	12	5	239	**8.80E-07**	**4.32E+00**	KRAS;NRAS;PIK3CA;HRAS;PDGFRA
REACTOME_EPH_EPHRIN_SIGNALING	**2.01E-04*****	92	9	769	**8.87E-07**	6.59E-01	EPHA2;ROCK2;EPHA8;EPHA10; EPHA6;HRAS;LIMK1;EPHB4; EPHB1
REACTOME_SIGNALING_BY_ERBB2_IN_CANCER	**2.01E-04*****	26	6	399	**1.20E-06**	**2.46E+00**	KRAS;EGFR;ERBB2; NRAS;PIK3CA;HRAS
REACTOME_SIGNALING_BY_VEGF	**2.01E-04*****	106	9	966	**1.87E-06**	7.03E-01	KRAS;MAPK14;PRKCA;ROCK2; CTNNB1;AXL;NRAS;PIK3CA; HRAS
REACTOME_CONSTITUTIVE_SIGNALING_BY_EGFRVIII	**2.01E-04*****	15	5	310	**1.87E-06**	**3.42E+00**	EGFR;KRAS;NRAS;PIK3CA;HRAS
REACTOME_MAPK_FAMILY_SIGNALING_CASCADES	**2.01E-04*****	327	14	2537	**1.87E-06**	2.51E-01	KRAS;EGFR;BRAF;ERBB2; NF1;KIT;NRAS;PDGFRB; PIK3CA;HRAS;FGFR2;JAK1; JAK3;PDGFRA
REACTOME_DOWNSTREAM_SIGNAL_TRANSDUCTION	**2.01E-04*****	29	6	465	**1.87E-06**	1.86E+00	KRAS;NRAS;PDGFRB; PIK3CA;HRAS;PDGFRA
REACTOME_SIGNALING_BY_ERBB2	**2.01E-04*****	50	7	697	**1.87E-06**	1.48E+00	KRAS;PRKCA;EGFR;ERBB2; NRAS;PIK3CA;HRAS
REACTOME_GRB2_EVENTS_IN_ERBB2_SIGNALING	**2.01E-04*****	16	5	240	**2.22E-06**	**3.42E+00**	EGFR;KRAS;ERBB2;NRAS;HRAS
REACTOME_RAS_SIGNALING_DOWNSTREAM _OF_NF1_LOSS_OF_FUNCTION_VARIANTS	**2.01E-04*****	7	4	81	**2.56E-06**	**4.32E+00**	KRAS;NF1;NRAS;HRAS
REACTOME_NEGATIVE_REGULATION_OF _THE_PI3K_AKT_NETWORK	**2.01E-04*****	113	9	1216	**2.56E-06**	6.93E-01	EGFR;ERBB2;KIT;PTEN; PDGFRB;PIK3CA;INSR;FGFR2; PDGFRA
REACTOME_SIGNALING_BY_NTRK3_TRKC	**2.01E-04*****	17	5	304	**2.60E-06**	**3.09E+00**	KRAS;NRAS;PIK3CA;HRAS;NTRK3
REACTOME_CONSTITUTIVE_SIGNALING _BY_LIGAND_RESPONSIVE _EGFR_CANCER_VARIANTS	**2.01E-04*****	19	5	392	**4.36E-06**	**2.82E+00**	KRAS;EGFR;NRAS;PIK3CA;HRAS
REACTOME_ACTIVATED_NTRK3 _SIGNALS_THROUGH_RAS	**2.01E-04*****	8	4	163	**4.36E-06**	**8.82E+00**	KRAS;HRAS;NRAS;NTRK3

12.4. Reactome pathways predicted specifically with GNNenrich on the Gastric cancer list for $\beta = 1$

Table 32. Selection of Reactome pathways predicted specifically with GNNenrich with $\beta = 1$ for the GC use case. Genes overlapping with MalaCards are highlighted in blue when the overlap with the query is empty.

Pathway Name	BH	Size	Overlap	PPI	g:Profiler	EnrichNet	Gene in the overlap
REACTOME_FCERI_MEDIATED_MAPK_ACTIVATION	**3.45E-04*****	87	3	440	0.062425366	0.762706881	KRAS;HRAS;NRAS
REACTOME_G2_M_CHECKPOINTS	**0.005442299****	167	3	682	0.185923248	0.019883914	ATM;TP53;CDC7
REACTOME_ENERGY_DEPENDENT_REGULATION _OF_MTOR_BY_LKB1_AMPK	**0.0008455*****	29	2	138	0.050781884	0.597947156	STK11;PRKAA2
REACTOME_SIGNALING_BY_CSF3_G_CSF	**0.00646****	30	2	377	0.053085191	0.81533846	JAK1;KRAS
REACTOME_CYCLIN_D_ASSOCIATED_EVENTS_IN_G1	**0.009374022****	47	2	389	0.100319307	0.09658846	CDKN2A;RB1
REACTOME_G2_M_DNA_DAMAGE_CHECKPOINT	**0.002635325****	94	2	308	0.214718781	0.301824946	ATM;TP53
REACTOME_SENESCENCE_ASSOCIATED _SECRETORY_PHENOTYPE_SASP	**0.045436435***	111	2	365	0.258994316	-0.18466154	CDKN2A;RPS6KA3
REACTOME_MAP3K8_TPL2_DEPENDENT _MAPK1_3_ACTIVATION	**3.45E-04*****	16	0	152	NA	-0.18466154	['BTRC', 'CHUK', 'MAP2K1', 'MAP2K4', 'NFKB1']
REACTOME_IRAK2_MEDIATED_ACTIVATION _OF_TAK1_COMPLEX	**3.45E-04*****	10	0	125	NA	-0.18466154	
REACTOME_CHK1_CHK2_CDS1_MEDIATED _INACTIVATION_OF_CYCLIN_B_CDK1_COMPLEX	**3.45E-04*****	13	0	131	NA	-0.18466154	['CCNB1', 'CDK1', 'CHEK1', 'CHEK2', 'YWHAE', 'YWHAZ']
REACTOME_FLT3_SIGNALING_THROUGH _SRC_FAMILY_KINASES	**3.45E-04*****	6	0	82	NA	-0.18466154	['SYK']
REACTOME_PTK6_REGULATES_CELL_CYCLE	**3.45E-04*****	6	0	53	NA	-0.18466154	['CCND1', 'CCNE1', 'CDK2', 'CDK4', 'CDKN1B']
REACTOME_ACTIVATION_OF_BAD_AND _TRANSLOCATION_TO_MITOCHONDRIA	**0.000345102*****	15	0	140	NA	-0.18466154	['AKT1', 'BAD', 'BCL2', 'BID', 'YWHAE', 'YWHAZ']
REACTOME_P130CAS_LINKAGE_TO _MAPK_SIGNALING_FOR_INTEGRINS	**0.000345102*****	15	0	168	NA	-0.18466154	['FN1', 'ITGB3', 'PTK2', 'SRC']
REACTOME_ACTIVATED_NTRK2_SIGNALS_THROUGH_FYN	**0.000345102*****	7	0	120	NA	-0.18466154	['RAC1', 'SRC']
REACTOME_TRIF_MEDIATED_PROGRAMMED_CELL_DEATH	**0.000345102*****	9	0	31	NA	-0.18466154	['CASP8', 'CD14', 'FADD', 'TLR4']
REACTOME_FCERI_MEDIATED_NF_KB_ACTIVATION	**0.000626296*****	136	0	497	NA	-0.18466154	['BTRC', 'CHUK', 'NFKB1', 'NFKBIA', 'PDPK1', 'PSMB8', 'RELA']
REACTOME_GRB2_SOS_PROVIDES_LINKAGE _TO_MAPK_SIGNALING_FOR_INTEGRINS	**0.000626296*****	15	0	183	NA	-0.18466154	['FN1', 'GRB2', 'ITGB3', 'PTK2', 'SRC']
REACTOME_PROLACTIN_RECEPTOR_SIGNALING	**0.0008455*****	15	0	111	NA	-0.18466154	['BTRC', 'JAK2', 'PTPN11', 'RBX1', 'STAT5A']

12.5. Reactome pathways predicted specifically with GNNenrich on the Gastric cancer list for $\beta = 10$. Genes overlapping with MalaCards are highlighted in blue when the overlap with the query is empty.

For GC use case, GNNenrich specific results on Reactome includes 40 pathways (Table 33). For instance, we cite "REACTOME ENERGY DEPENDENT REGULATION OF MTOR BY LKB1 AMPK" [Ciccarese et al. (2019)], "REACTOME DOWNREGULATION OF ERBB2 SIGNALING" Li et al. (2017), "REACTOME DISASSEMBLY OF THE DESTRUCTION COMPLEX AND RECRUITMENT OF AXIN TO THE MEMBRANE" Peng et al. (2021) and "REACTOME NEGATIVE REGULATION OF FGFR2 SIGNALING" [Kunii et al. (2008); Tojjari et al. (2024)].

Table 33. Selection of Reactome pathways predicted specifically with GNNenrich with $\beta = 10$ for the GC use case. Genes overlapping with MalaCards are highlighted in blue when the overlap with the query is empty.

Pathway Name	BH	Size	Overlap	PPI	g:Profiler	EnrichNet	Gene in the overlap
REACTOME_FCERI_MEDIATED_MAPK_ACTIVATION	**2.38E-04*****	87	3	440	6.24E-02	7.63E-01	HRAS;NRAS;KRAS
REACTOME_ENERGY_DEPENDENT_REGULATION _OF_MTOR_BY_LKB1_AMPK	**1.03E-02***	29	2	138	5.08E-02	5.98E-01	PRKAA2;STK11
REACTOME_DOWNREGULATION_OF_ERBB2_SIGNALING	**1.37E-02***	29	2	330	5.08E-02	8.15E-01	ERBB2;EGFR
REACTOME_SIGNALING_BY_CSF3_G_CSF	**8.01E-03****	30	2	377	5.31E-02	8.15E-01	JAK1;KRAS
REACTOME_DISASSEMBLY_OF_THE_DESTRUCTION _COMPLEX_AND_RECRUITMENT _OF_AXIN_TO_THE_MEMBRANE	**3.20E-02***	31	2	202	5.51E-02	5.35E-01	APC;CTNNB1
REACTOME_NEGATIVE_REGULATION_OF_FGFR2_SIGNALING	**2.05E-02***	34	2	502	6.38E-02	1.75E-01	FGFR2;BRAF
REACTOME_MTOR_SIGNALLING	**2.62E-02***	41	2	216	8.59E-02	4.82E-01	PRKAA2;STK11
REACTOME_CYCLIN_D_ASSOCIATED_EVENTS_IN_G1	**1.49E-02***	47	2	389	1.00E-01	9.66E-02	RB1;CDKN2A
REACTOME_G2_M_DNA_DAMAGE_CHECKPOINT	**4.47E-02***	94	2	308	2.15E-01	3.02E-01	ATM;TP53
REACTOME_SENESCENCE_ASSOCIATED _SECRETORY_PHENOTYPE_SASP	**2.55E-02***	111	2	365	2.59E-01	-1.85E-01	RPS6KA3;CDKN2A
REACTOME_DRUG_MEDIATED_INHIBITION _OF_CDK4_CDK6_ACTIVITY	**5.37E-03****	5	0	40	NA	-1.85E-01	['CCND1', 'CCND2', 'CDK4', 'CDK6']
REACTOME_PTK6_REGULATES_CELL_CYCLE	**8.16E-03****	6	0	53	NA	-1.85E-01	['CCND1', 'CCNE1', 'CDK2', 'CDK4', 'CDKN1B']
REACTOME_ERYTHROPOIETIN_ACTIVATES_STAT5	**1.79E-02***	7	0	88	NA	-1.85E-01	['GRB2', 'HRAS', 'JAK2', 'KRAS', 'NRAS', 'PIK3CA', 'PIK3CB', 'PIK3CD', 'PIK3CG', 'PIK3R1', 'PLCG2', 'STAT5A']
REACTOME_INTERLEUKIN_23_SIGNALING	**1.87E-02***	9	0	74	NA	-1.85E-01	['JAK2', 'P4HB', 'STAT3']
REACTOME_ERYTHROPOIETIN_ACTIVATES _PHOSPHOLIPASE_C_GAMMA_PLCG	**2.43E-02***	7	0	97	NA	-1.85E-01	['JAK2', 'PLCG2']
REACTOME_G2_PHASE	**2.54E-02***	5	0	29	NA	-1.85E-01	['CCNA1', 'CCNA2', 'CDK2', 'E2F1']
REACTOME_IRAK2_MEDIATED_ACTIVATION _OF_TAK1_COMPLEX	**2.90E-02***	10	0	125	NA	-1.85E-01	
REACTOME_TRIF_MEDIATED_PROGRAMMED_CELL_DEATH	**3.71E-02***	9	0	31	NA	-1.85E-01	['CASP8', 'CD14', 'FADD', 'TLR4']
REACTOME_TNF_RECEPTOR_SUPERFAMILY _TNFSF_MEMBERS_MEDIATING _NON_CANONICAL_NF_KB_PATHWAY	**3.83E-02***	17	0	36	NA	-1.85E-01	['BIRC2', 'BIRC3', 'CD40', 'CD40LG', 'LTA', 'TNFRSF12A', 'TRAF2']
REACTOME_AKT_PHOSPHORYLATES_TARGETS _IN_THE_CYTOSOL	**4.67E-02***	14	0	100	NA	-1.85E-01	['AKT1', 'BAD', 'CASP9', 'CDKN1A', 'CDKN1B', 'CHUK', 'GSK3B', 'MDM2']

12.6. Reactome pathways predicted specifically with GNNenrich on the Gastric cancer list for $\beta = 40$

Table 34. Selection of Reactome pathways predicted specifically with GNNenrich with $\beta = 40$ for the GC use case.

Pathway Name	BH	Size	Overlap	PPI	g:Profiler	EnrichNet	Gene in the overlap
REACTOME_CELLULAR_RESPONSES_TO_STIMULI	**3.95E-02***	826	11	1909	7.00E-02	-3.08E-02	MAPK14;PRKAA2;CDKN2A; ERN1;RPS6KA3;EIF2AK1; MINK1;TP53; RB1;ATM;MAP4K4
REACTOME_DEUBIQUITINATION	**4.15E-02***	299	6	923	5.09E-02	1.41E-01	TGFBR2;PTEN;APC; SMAD4;TP53;TRRAP
REACTOME_SIGNALING_BY_WNT	**4.66E-02***	330	6	1365	7.10E-02	4.15E-02	KRAS;PRKCA;CTNNB1; MEN1;APC;TRRAP
REACTOME_ESR_MEDIATED_SIGNALING	**2.42E-02***	220	5	1097	5.28E-02	1.52E-01	KRAS;EGFR;NRAS;PIK3CA; HRAS
REACTOME_FCERI_MEDIATED_MAPK_ACTIVATION	**3.64E-04*****	87	3	440	6.24E-02	7.63E-01	KRAS;HRAS;NRAS
REACTOME_DNA_DAMAGE_TELOMERE _STRESS_INDUCED_SENESCENCE	**7.03E-03****	79	3	134	5.10E-02	1.32E+00	TP53;RB1;ATM
REACTOME_FORMATION_OF_THE _BETA_CATENIN_TCF_TRANSACTIVATING _COMPLEX	**9.71E-03** **	91	3	174	6.87E-02	8.95E-01	MEN1;TRRAP;CTNNB1
REACTOME_DISEASES_OF_PROGRAMMED_CELL_DEATH	**2.21E-02***	104	3	192	8.84E-02	3.78E-01	TP53;CDKN2A;MLKL
REACTOME_SIGNALING_BY_CSF3_G_CSF	**8.46E-03****	30	2	377	5.31E-02	8.15E-01	KRAS;JAK1
REACTOME_ENERGY_DEPENDENT _REGULATION_OF_MTOR_BY _LKB1_AMPK	**8.46E-03****	29	2	138	5.08E-02	5.98E-01	STK11;PRKAA2
REACTOME_DOWNREGULATION_OF_ERBB2_SIGNALING	**1.26E-02***	29	2	330	5.08E-02	8.15E-01	ERBB2;EGFR
REACTOME_NEGATIVE_REGULATION_OF_FGFR2_SIGNALING	1.57E-02	34	2	502	6.38E-02	1.75E-01	FGFR2;BRAF
REACTOME_MTOR_SIGNALLING	2.38E-02	41	2	216	8.59E-02	4.82E-01	STK11;PRKAA2
REACTOME_MAPK3_ERK1_ACTIVATION	1.46E-02	10	1	139	1.34E-01	1.62E+00	JAK1
REACTOME_INTERLEUKIN_6_SIGNALING	1.94E-02	11	1	151	1.44E-01	1.10E+00	JAK1
REACTOME_NF_KB_ACTIVATION_THROUGH _FADD_RIP_1_PATHWAY_MEDIATED_BY_CASPASE_8_AND_10	2.09E-02	13	1	49	1.60E-01	8.15E-01	CASP10
REACTOME_INTERLEUKIN_35_SIGNALLING	2.38E-02	12	1	95	1.53E-01	9.40E-01	JAK1
REACTOME_FBXW7_MUTANTS_AND_NOTCH1_IN_CANCER	2.75E-02	5	1	24	8.63E-02	1.62E+00	FBXW7
REACTOME_REGULATION_OF_IFNG_SIGNALING	2.88E-02	14	1	139	1.67E-01	6.34E-01	JAK1
REACTOME_ABERRANT_REGULATION_OF _MITOTIC_G1_S_TRANSITION_IN_CANCER_DUE_TO_RB1_DEFECTS	3.31E-02	17	1	96	1.86E-01	5.08E-01	RB1

13. Synthesis of specific results identified by GNNenrich

Table 35. Distribution of specific pathways based on the number of genes/proteins in the overlap for two β values: 10 and 40

	PD/WikiPatways			GC/WikiPathways			PD/KEGG			GC/KEGG			PD/Reactome			GC/Reactome		
Overlap/β	1	10	40	1	10	40	1	10	40	1	10	40	1	10	40	1	10	40
0		10			9		1							2			10	
1	18	25	12		2	2							3	1	1	22	20	16
2	7	25	27	7	11	21		3	4			1		1	1	5	9	9
3	3	17	16			3		7	7			2	1	3	4	2	1	4
4	1	5	5		1	6		2	2									
5	3	3	3			2						1		1	1			1
6																		2
7								1	1									
8	2	3	3															
9																		
10														1	1			
11																		1

14. Queries for PD and GC

The two lists, PD and GC, are available on the EnrichNet web interface at the following link: https://lcsb-enrichnet.uni.lu/enrichnet/index.php

Table 36. Parkinson and Gastric cancer lists

PARKINSON	LRRK2;PARK2;SNCA;APOE;PINK1;MAPT;GBA;COMT;UCHL1;MAOB;DRD2;CYP2D6;PARK7;SLC6A3;BDNF;NR4A2;GIGYF2;DRD3;GSTM1;MTHFR;PON1;GSTP1;NAT2;HFE;ATP13A2;<br>IL1A;IL1B;MAOA;ND1;ABCB1;FGF20;GSTT1;FMR1;SLC6A4;TNF;SOD2;PITX3;ND3;NQO1;DRD4;LINGO1;SEMA5A;CYP2E1;ND2;ATXN3;NOS1;NOS2A;POLG;ATXN2;<br>TBP;TFAM;TRNG;GSTO2;SERPINA3;DBH;ESR1;ESR2;PARK10;HTRA2;HTR2A;IL6;GSTO1;SNCAIP;BST1;TH;IL10;LMX1B;GSTM3;HNMT;FTL;GRN;GRIN2B;GSK3B;EPHX1;<br>ACE;CYP1A1;CYP1A2;ND5;ATP6;COX2;NQO2;TRNQ;NEDD9;NUCKS1;CACNA1A;PRNP;USP40;CALB1;SNCG;TRNK;NDUFV2;NOS3;MSX1;COX1;ATP8;ASCL1;PARK11;LMX1A;MTR<br>;ND4;CYTB;NUDT1;CYP1B1;ADRA2A;CST3;PACRG;CHRNA4;DRD5;ELAVL4;A2M;USP24;TOR1A;GCH1;GSTZ1;STH;FRAXA;FTH1;HMOX1;IL8;IL1RN;CAST;PDXK;UNC5C;CCK;CCKAR;CCKBR
GASTRIC CANCER	ENSG00000039068;ENSG00000141736;ENSG00000121879;ENSG00000066468;ENSG00000070886;ENSG00000072518;ENSG00000092439;ENSG00000112742;ENSG00000177189;<br>ENSG00000204217;ENSG00000134602;ENSG00000214102;ENSG00000165238;ENSG00000162409;ENSG00000113263;ENSG00000126562;ENSG00000006432;<br>ENSG00000137948;ENSG00000112062;ENSG00000198870;ENSG00000102096;ENSG00000154358;ENSG00000160602;ENSG00000196367;ENSG00000105639;ENSG00000071054;<br>ENSG00000080224;ENSG00000142627;ENSG00000169925;ENSG00000171105;ENSG00000097046;ENSG00000133083;ENSG00000140538;ENSG00000147044;<br>ENSG00000106683;ENSG00000167601;ENSG00000163513;ENSG00000168404;ENSG00000121101;ENSG00000198055;ENSG00000154928;ENSG00000116141;ENSG00000146872;<br>ENSG00000061938;ENSG00000086232;ENSG00000151239;ENSG00000183317;ENSG00000141503;ENSG00000083290;ENSG00000047936;ENSG00000149311;ENSG00000154229;<br>ENSG00000120539;ENSG00000162434;ENSG00000113721;ENSG00000204256;ENSG00000178607;ENSG00000072195;ENSG00000128881;ENSG00000155657;ENSG00000107779;<br>ENSG00000105287;ENSG00000196411;ENSG00000176601;ENSG00000138696;ENSG00000121989;ENSG00000160469;ENSG00000134318;ENSG00000143479;<br>ENSG00000133703;ENSG00000134853;ENSG00000134982;ENSG00000109670;ENSG00000196712;ENSG00000174775;ENSG00000133895;ENSG00000095002;<br>ENSG00000141646;ENSG00000116062;ENSG00000157764;ENSG00000184771;ENSG00000171862;ENSG00000146648;ENSG00000147889;ENSG00000139687;ENSG00000118046;<br>ENSG00000168036;ENSG00000141510;ENSG00000213281;ENSG00000076242;ENSG00000132781;ENSG00000067082;ENSG00000135821;ENSG00000136689;<br>ENSG00000125538;ENSG00000095906;ENSG00000003400;ENSG00000125347;ENSG00000157404;ENSG00000196090

15. EnrichNet thresholds

Table 37. EnrichNet thresholds for Parkinson and Gastric cancer lists

	Query	EnrichNet thresholds
WikiPathways	PD	1,299
	GC	0,814
KEGG	PD	0,889
	GC	0,739
Reactome	PD	2,114
	GC	1,897

16. Computing environment

We used PyTorch as the deep learning framework, along with Torch Geometric for graph-based computations. These frameworks provide efficient tools for handling deep learning models, particularly in graph neural networks (GNNs).
For training, we utilized a GPU-enabled computing environment to speed up model training, as training GNN models on large protein-protein interaction networks can be computationally intensive. Our environment included PyTorch 1.6, along with dependencies such as torch-scatter, torch-sparse.
For inference, a GPU is not mandatory, and the model can be run efficiently on a standard CPU. Since inference primarily involves forward pass computations rather than backpropagation, it has significantly lower computational requirements compared to training. We recommend using a machine with sufficient RAM and processing power to handle matrix operations efficiently, but no specialized hardware is required.

17. Supplementary figures

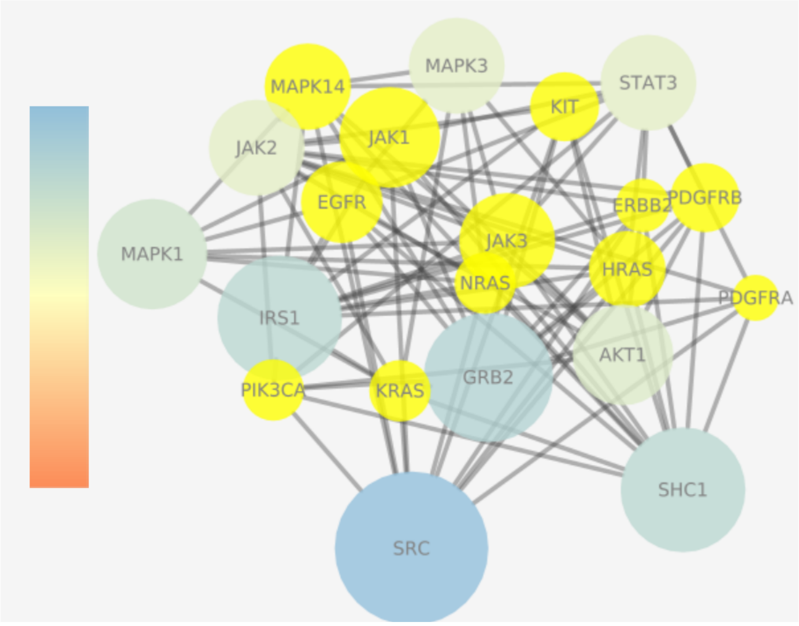

(a) EPO receptor signaling (WP581)

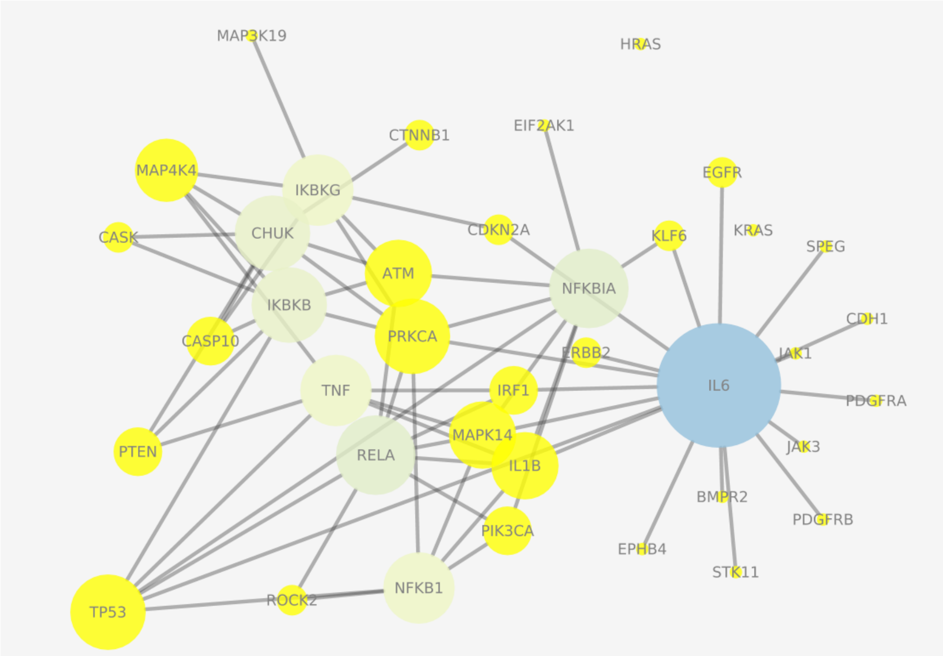

(b) ALTERED GLYCOSYLATION OF MUC1 IN TUMOR MICROENVIRONMENT (WP4480)

Fig. 2: Cytoscape visualization of protein-protein interactions. Gastric cancer proteins are shown in yellow, while proteins in the WP581 and WP4480 pathways are displayed in different colors. The size of each node is proportional to its degree. All nodes with a degree below 8 have been filtered out in WP581.

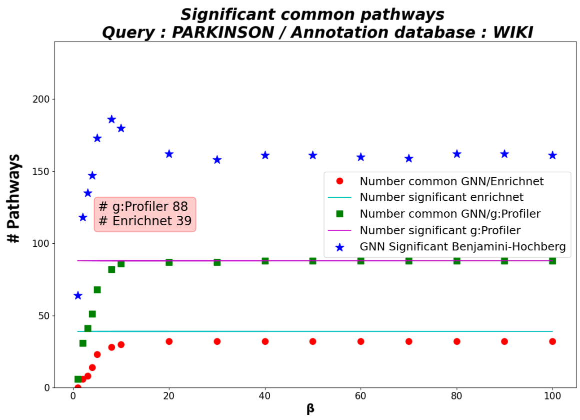

(a)

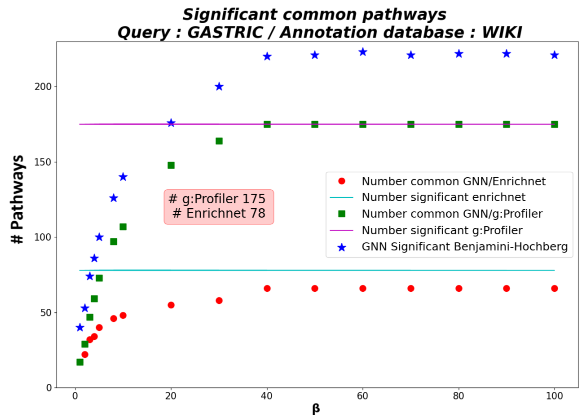

(b)

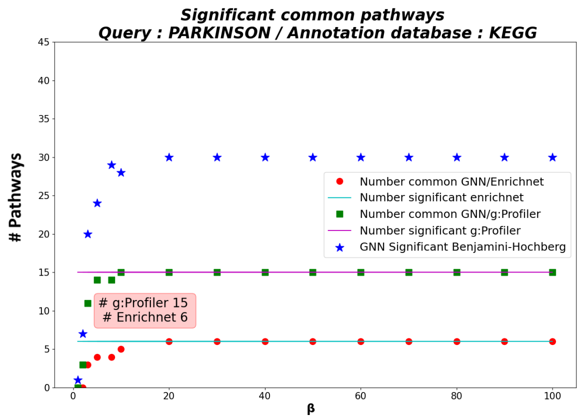

(c)

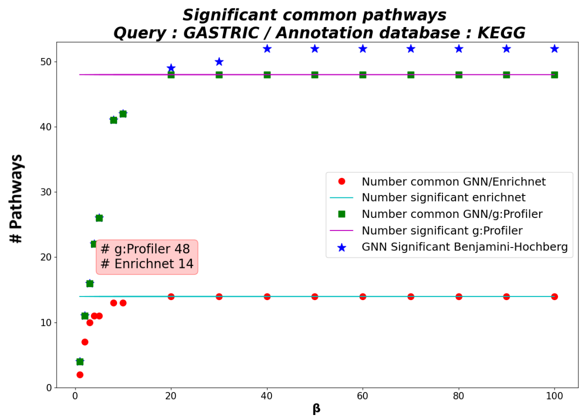

(d)

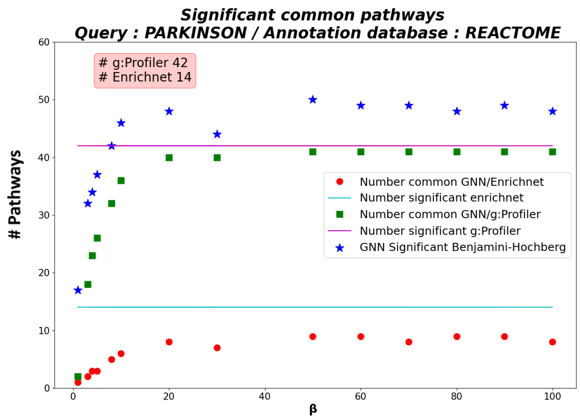

(e)

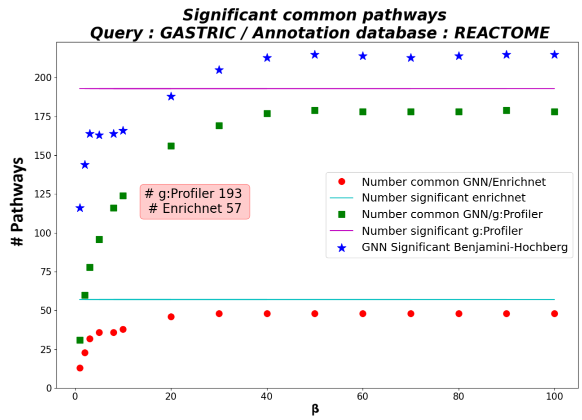

(f)

Fig. 3: Significant pathways variation related to β coefficient and common results with g:Profiler and EnrichNet for β between 1 and 100. Only strongly correlated proteins are considered (correlation threshold > 0.9)

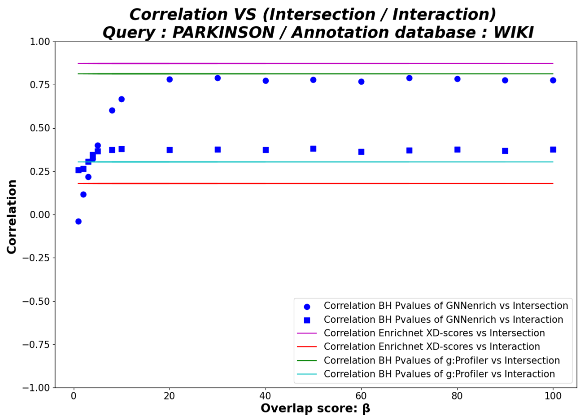

(a)

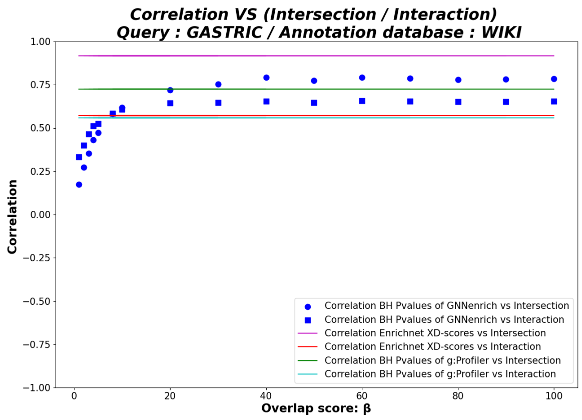

(b)

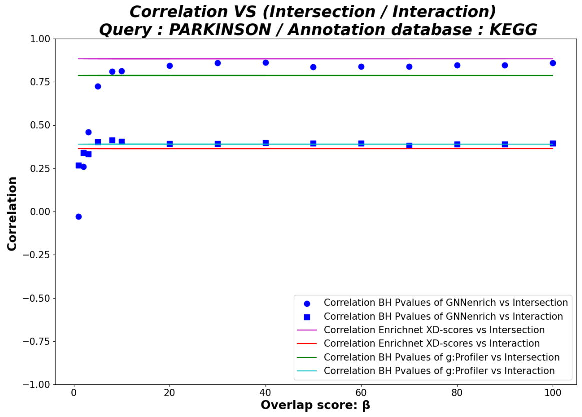

(c)

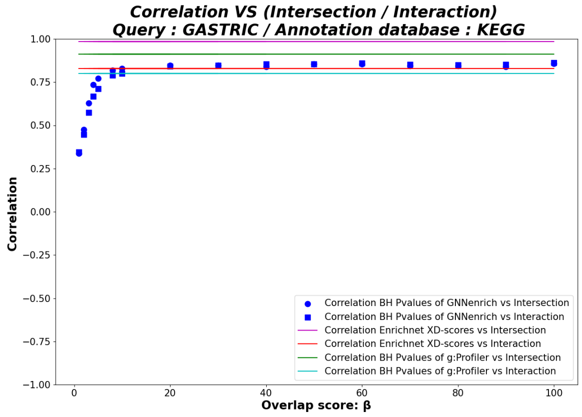

(d)

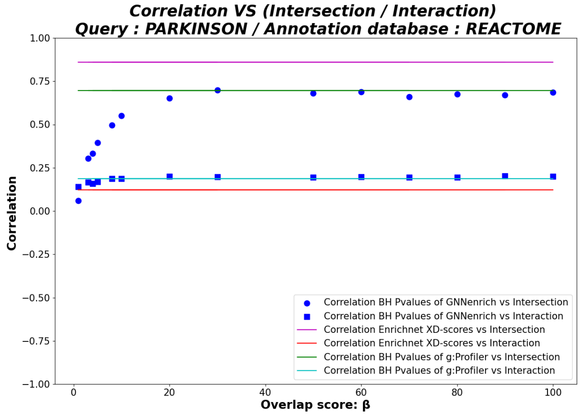

(e)

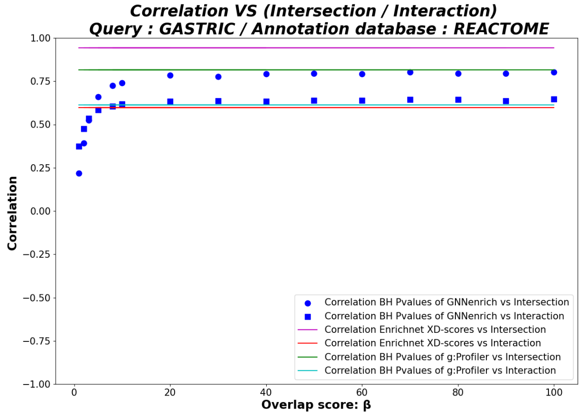

(f)

Fig. 4: Correlation analysis between GNNenrich-based enrichment results and those from g:Profiler and EnrichNet for different pathway databases (WikiPathways, KEGG, and Reactome). The Pearson correlation coefficients were computed for (β) in [1..100] across three methods: g:Profiler, EnrichNet, and GNNenrich. Results are shown separately for Parkinson's disease (left column) and gastric cancer (right column).

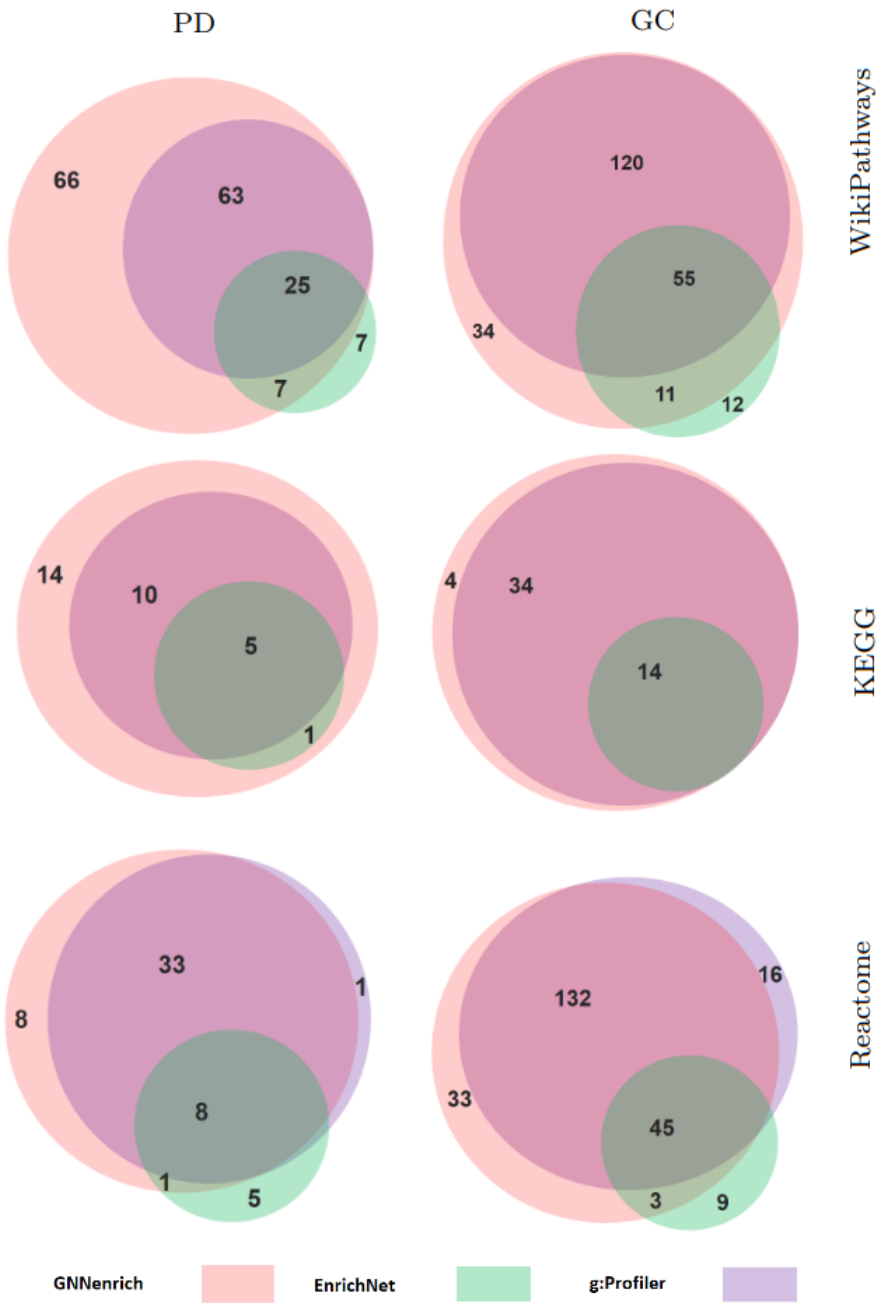

Fig. 5: Common pathways between GNNenrich, EnrichNet and g:Profiler on WikiPathways. Overlap weighting coefficient has been set $\beta = 40$ for GNNenrich. P-values are detailed in table 41

18. The robustness of the GNNenrich.

For the learning phase, GNN-based model robustness was examined in previous studies, ensuring the model's validity when dealing with partial subgraphs extracted from STRING. For instance, Chen et al. (2019) randomly selected 1,690 and 5,189 proteins from the Homo sapiens subset of STRING, ensuring that their sequence identity did not exceed 40%. They generated two distinct subsets, SHS27k and SHS148k, containing 7,624 and 44,488 multi-label interactions, respectively. GNN-PPI achieves an accuracy of 73.1% when trained on the SHS148k dataset and approximatively 66.89% for the smaller set SHS27k when tested on the larger STRING dataset. This approach is specifically designed to assess the model's robustness to generalize when faced with partially missing or unknown data, highlighting its capacity to maintain a fair level of learning across datasets of varying sizes.
For the inference stage, we evaluated the robustness of our method with a particular focus on the pathway scoring step, which constitutes our main contribution. Specifically, we assessed how the method handles missing interactions by analyzing the impact of different correlation thresholds between protein embeddings. We recall the principle of this filtering step in the figure 6. It consists of applying a

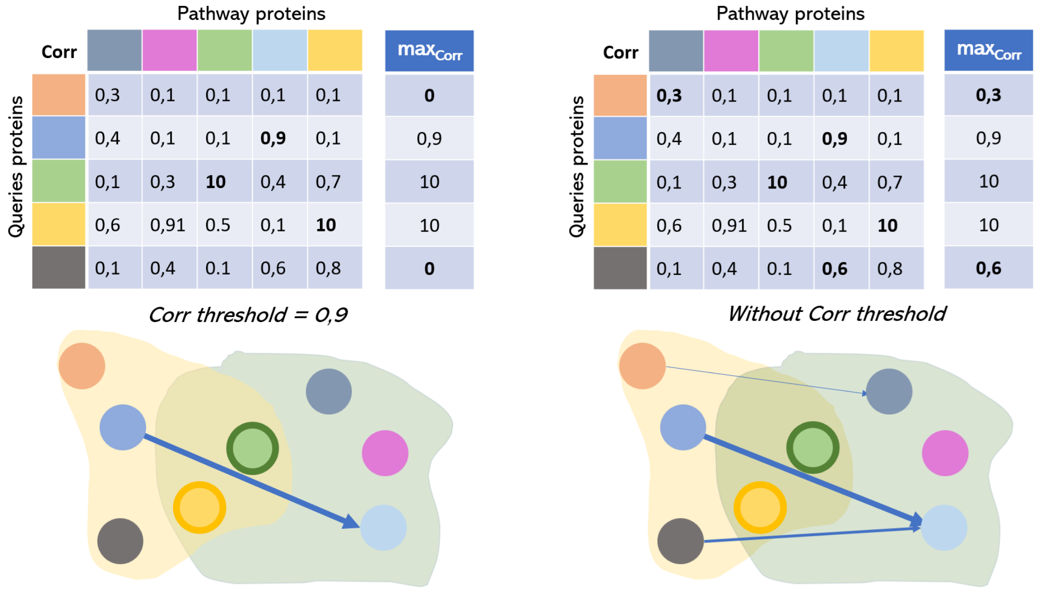

Fig. 6: Illustration of the correlation-based filtering step applied to the embedding similarity matrix. The figure compares two conditions: no filtering (threshold = 0) and stringent filtering (threshold = 0.9), where only strongly correlated embeddings are retained. This step is designed to preserve only the most reliable interaction relationships, as used in the main analysis.

configurable correlation-based filtering to the embedding correlation matrix, retaining only those pairs with strong correlations — and thus more likely to reflect reliable interaction relationships. The 0.9 threshold, as proposed in the article, ensures that only correlations exceeding this value are considered in the enrichment calculation, thereby discarding weaker correlations limiting the noise introduced by weaker interactions in the enrichment determination process. We also tested other thresholds, namely 0.7, 0.5, and 0, with the latter including all interactions without post-filtering. The general model's behavior remains consistent across these different thresholds.
The proportion of STRING database interactions neglected at each step can be inferred from the histogram in 7, which presents the distribution of correlation scores for all interactions in the STRING database.
The results obtained without post-filtering are provided in figure 8. The variation in the number of significant pathways as a function of β, as well as the overlap between those identified by g:Profiler and EnrichNet, is represented. The overall behavior is closely similar to the patterns observed in figures 3a and 3b.

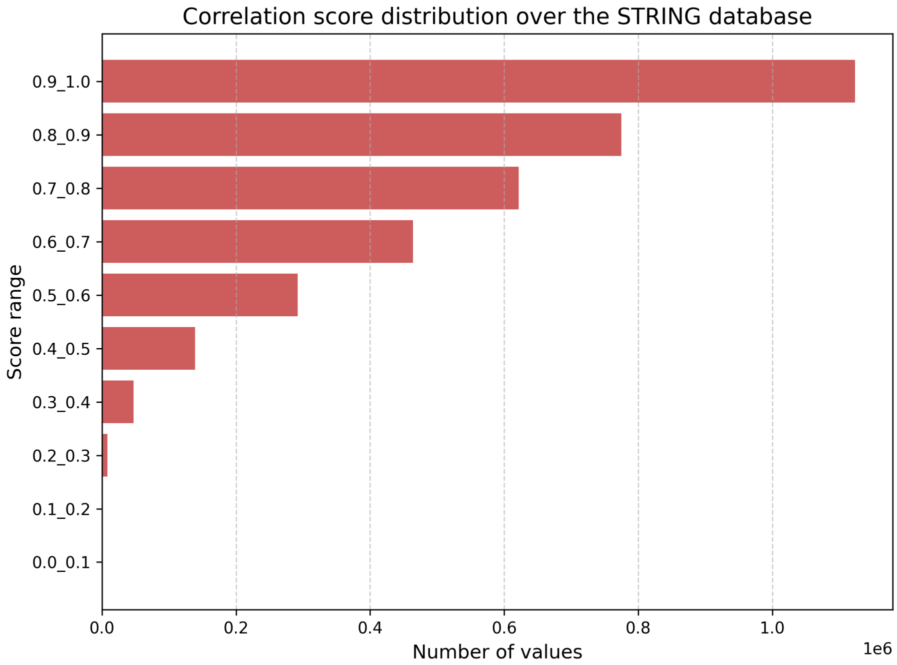

Fig. 7: Cumulative histogram of pairwise correlation scores predicted by the GNN model across the STRING protein–protein interaction database. Each bar reflects the total number of predicted scores for the corresponding range.

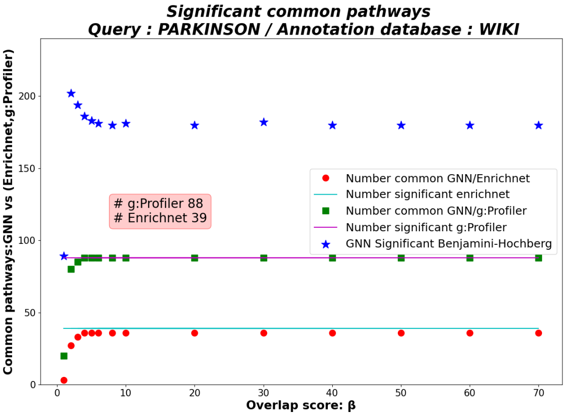

(a)

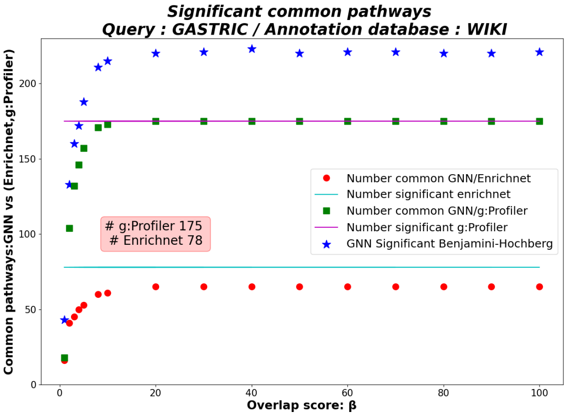

(b)

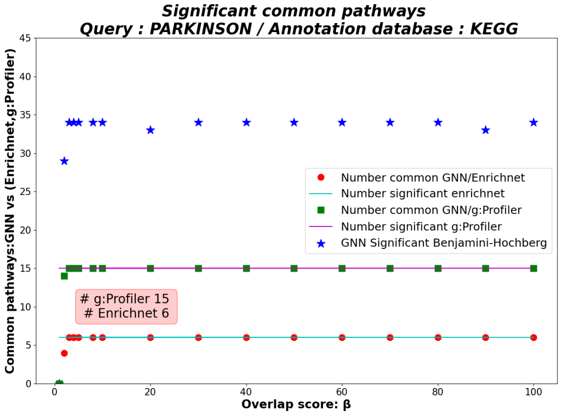

(c)

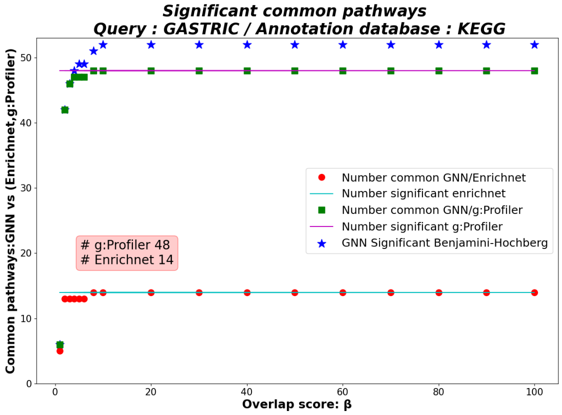

(d)

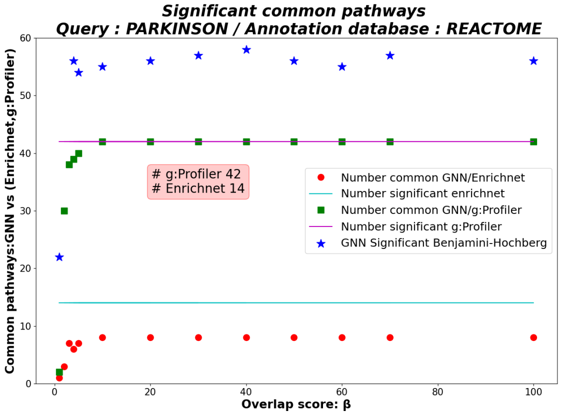

(e)

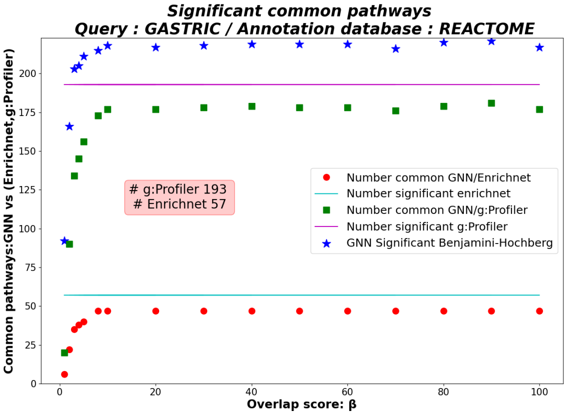

(f)

Fig. 8: Significant pathways variation related to β coefficient and common results with g:Profiler and EnrichNet for β between 1 and 100 without applying any correlation threshold

19. Biais relatif to Hub genes

We acknowledge the concern regarding potential biases introduced by hub proteins—those with a high number of interactions in protein-protein interaction (PPI) networks—which can lead to a skewed amplification of pathway enrichment results.
To address this, we conducted a systematic comparison of hub-related bias across the three functional enrichment methods: GNNenrich, EnrichNet, and g:Profiler. Specifically, we aimed to assess whether GNNenrich tends to favor highly connected proteins more or less than the other two methods.
To define hub proteins, we ranked all entries in the STRING V11 database according to their degree, i.e., the number of known or predicted interactions. We considered the top 15% of proteins—those with more than 142 interactions—as hub proteins since it roughly corresponds to the protein degree value from which the degree distribution starts to flatten, resulting in a total of 2,417 hubs (Figure 9). We then examined how these hub proteins overlapped with disease-associated genes from our two case studies: Parkinson's disease (PD) and gastric cancer (GC). For PD, 22 genes were identified as hubs, among which only IL6 and LRRK2 belonged to the top 1% most connected proteins. In the case of GC, 36 hub genes were identified, with 10 of them—including TP53, EGFR, and KRAS—falling into the top 1%. This setup provides two distinct biological contexts with different proportions of hubs, allowing us to evaluate how hub presence may influence enrichment results.

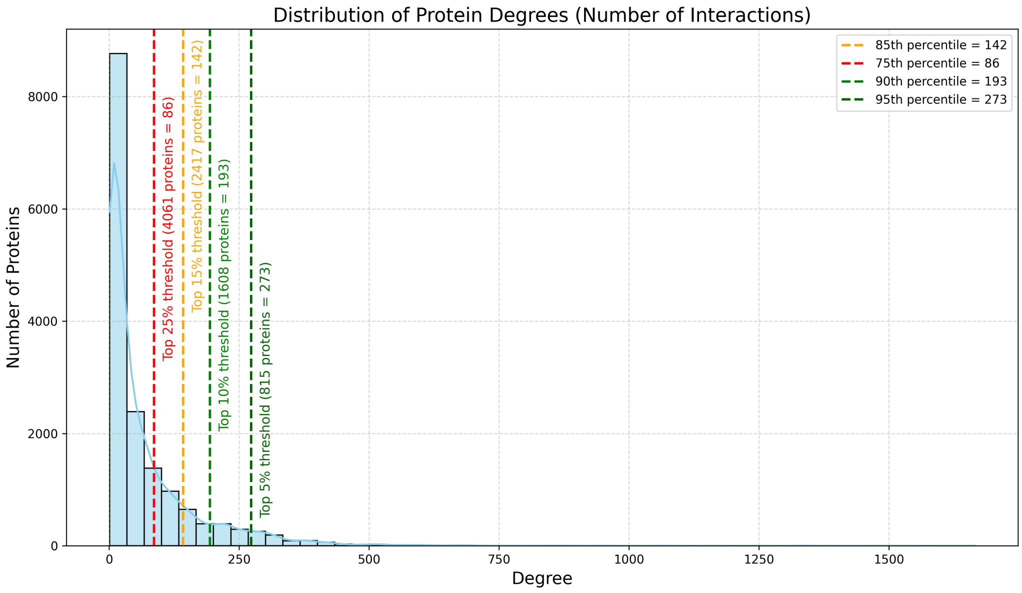

Fig. 9: Distribution of proteins in the STRING database according to their degree: illustration of the top-k proteins considered as hubs for the bias analysis.

To illustrate the presence of hub genes within WikiPathways, we analyzed the degree distribution of genes represented in the database, which contains 32,368 gene entries (including duplicates). Among the hub genes—defined as those in the top 15% by degree—there are 12,822 total occurrences across the pathways. Conversely, 855 hub genes are not represented in any pathway within the WikiPathways database. Overall, hub genes account for approximately 39.61% of all gene occurrences in WikiPathways, highlighting their broad and repeated involvement across multiple biological functions (figure 11).

Assessing the Role of Query Hubs in Enrichment Outcomes: GNNenrich Compared to g:Profiler and EnrichNet

We first assessed whether genes with more interactions in queries tend to be associated with a higher proportion of enriched pathways. Within each query, genes were ranked in ascending order, with those on the left exhibiting higher STRING interaction degrees.
For each gene, we then calculated the proportion of significant pathways in which it was involved, relative to the total number of significant pathways by each method, such as:

$$\text{Normalized occurrence (gene)} = \frac{\text{Number of significant pathways that include the gene}}{\text{Total number of significant pathways}} \tag{5}$$

The resulting distribution, shown in (12) and (13) reveals similar trends between GNNenrich and g:Profiler, particularly for genes with high interaction counts. In contrast, EnrichNet yielded a different pattern, suggesting a divergence in how the methods may treat highly connected proteins. This preliminary observation supports the idea that GNNenrich does not introduce strong hub-related biases in enrichment detection in comparison with the methods studied here. Although the difference observed for $\beta = 10$ is slightly higher than that for $\beta = 40$, overall behavior of GNNenrich with respect to hub genes remains largely consistent with that of g:Profiler. For further

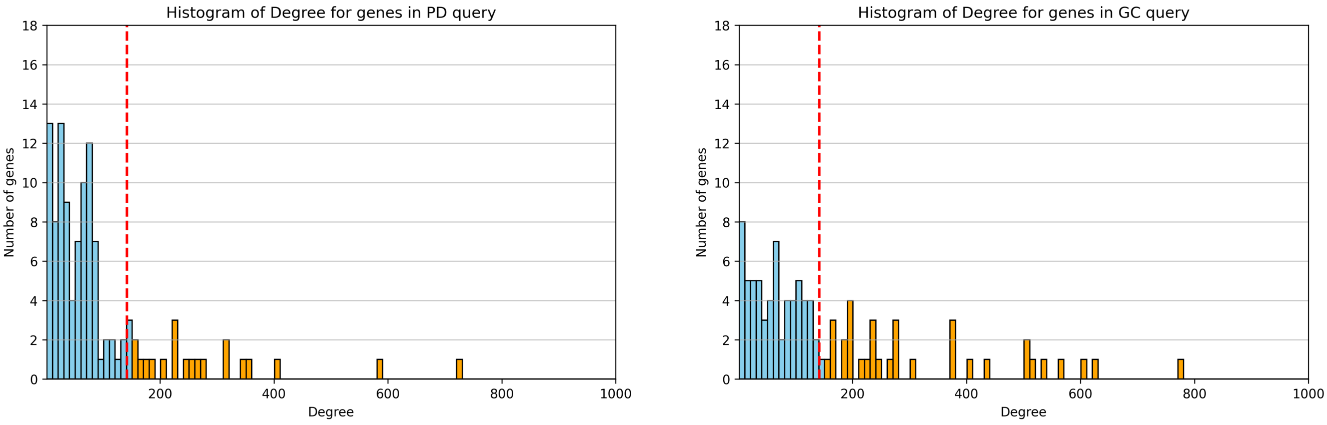

(a) (b)

Fig. 10: Distributions of gene interaction degrees for genes involved in Parkinson and Gastric cancer queries.

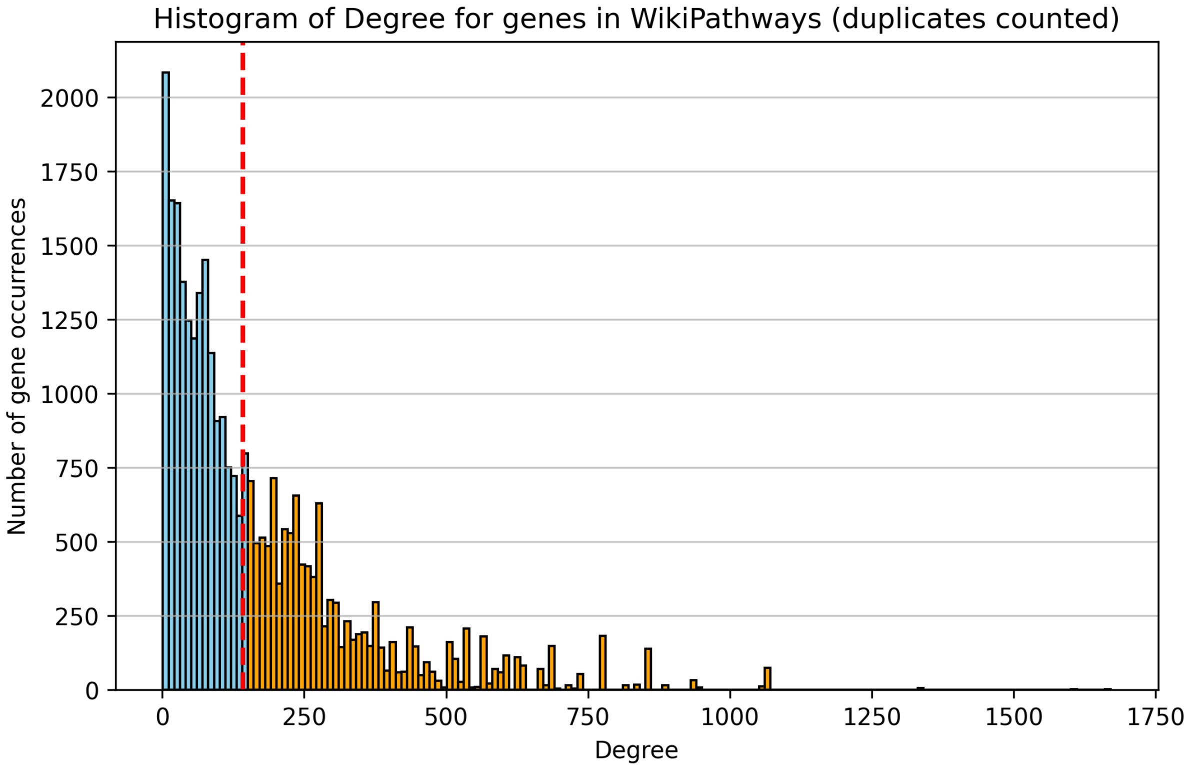

Fig. 11: Distribution of degrees for genes found in WikiPathways. Duplicate gene occurrences across pathways are counted. A vertical red dashed line marks a degree threshold of 142 and all degree ranges above this threshold are highlighted in orange.

illustration, (Figure 17) and (Figure 18) present the results for $\beta = 40$ on WikiPathways. (Figure 19), (Figure 20), (Figure 21) and (Figure 22) present the results for KEGG and Reactome. These results suggest that the pathway differences detected by GNNenrich are not mainly caused by hub genes in the query.

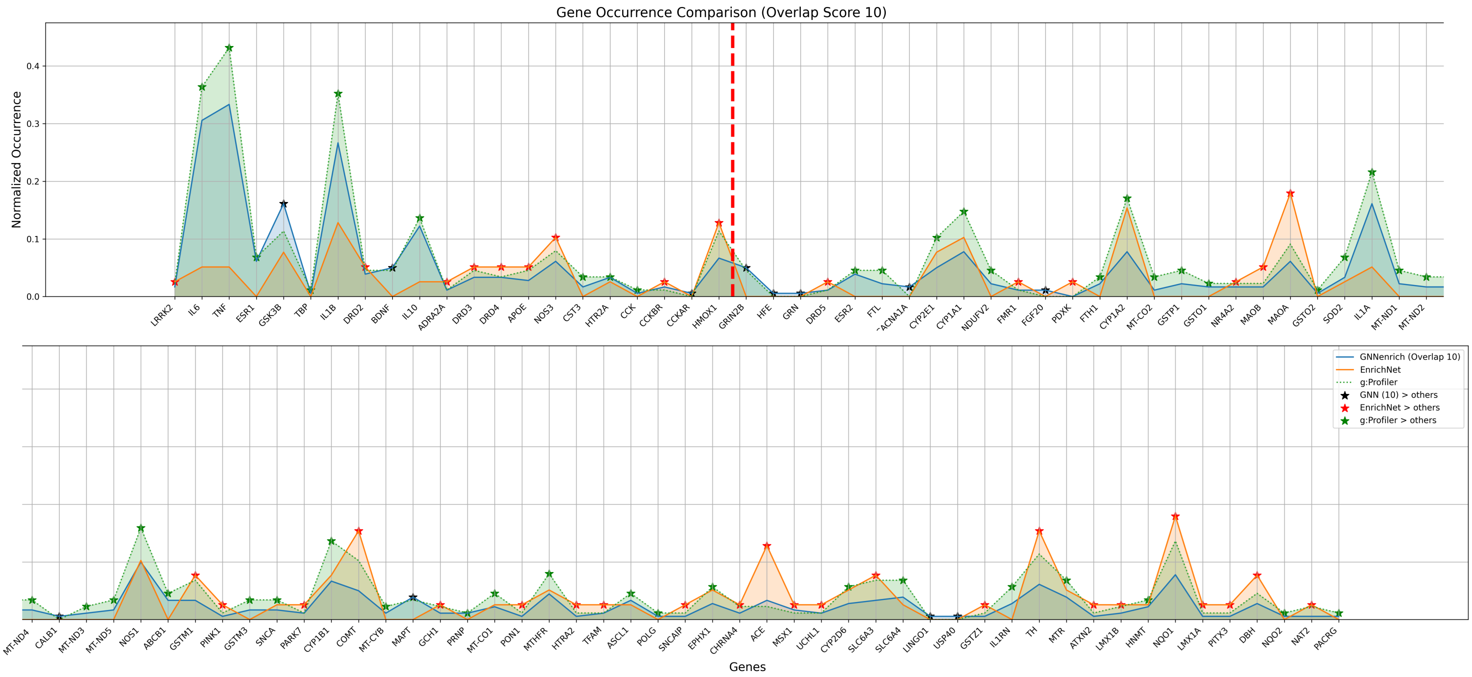

Fig. 12: Normalized gene occurrences across EnrichNet, g:Profiler, and GNNenrich for **WikiPathways / Parkinson** (*overlap score* = 10).

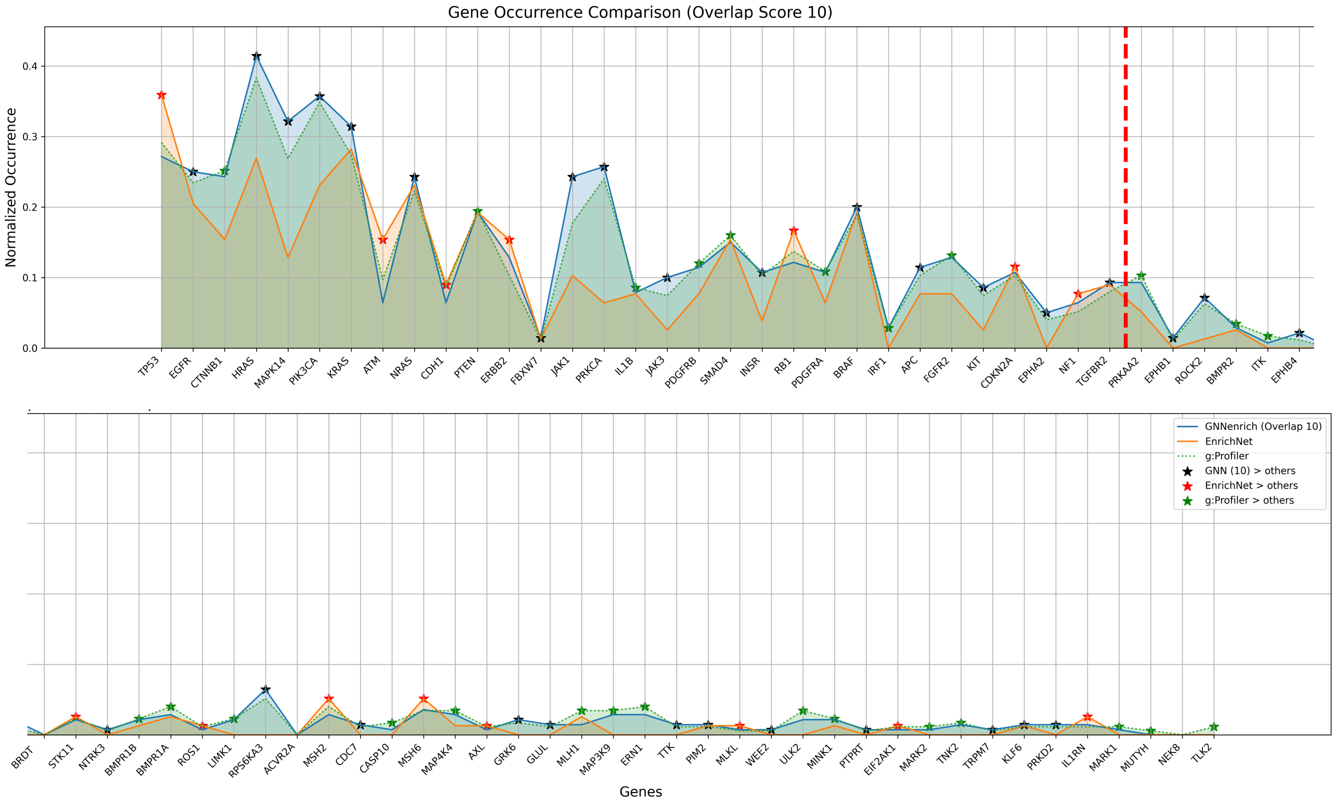

Fig. 13: Normalized gene occurrences across EnrichNet, g:Profiler, and GNNenrich for **WikiPathways / Gastric cancer** (*overlap score* = 10).

Evaluating the Proportion of Hub Proteins in Enriched Pathways

To gain a broader perspective on the impact of hub genes on our results, we further investigated this effect using our previous selection of the top 15% of genes with the highest number of interactions. From this selection, to assess the influence of hub proteins across the three pathway databases (WikiPathways, KEGG, and Reactome), we introduced a metric termed Hub Redundancy. This metric quantifies the proportion of hub gene/protein occurrences among all gene/protein occurrences within significant pathways, reflecting the influence of highly connected nodes on the enrichment results, such as:

$$\text{Hub redundancy} = \frac{\text{Number of Hub gene occurences in significant pathways}}{\text{Total number of gene occurences in significant pathways}} \tag{6}$$

This indicator is intended to highlight potential discrepancies between methods regarding the proportion of hub genes among those

Table 38. Comparison of hub gene redundancy across methods (GNNenrich, g:Profiler, EnrichNet) for different pathway databases (WikiPathways, KEGG, Reactome) and disease contexts (Parkinson, Gastric cancer). Bold values indicate the highest ratio of hub redundancy within each context.

Pathway DB	Method	Parkinson			Gastric cancer		
		Total hubs R	Total genes R	Hub's Red.	Total hubs R	Total genes R	Hub's Red.
WikiPathways	EnrichNet	176	654	26.9	1365	2364	**57.7**
	g:Profiler	1543	4860	31.8	6650	12988	51.2
	GNNenrich(10)	3697	9703	**38.1**	5801	10790	53.7
	GNNenrich(40)	3285	9018	**36.4**	7536	15407	48.9
KEGG	EnrichNet	5	286	1.7	576	826	**69.7**
	g:Profiler	371	1508	24.6	2754	5042	54.6
	GNNenrich(10)	787	2616	**30.1**	2526	4591	55.0
	GNNenrich(40)	788	2653	**29.7**	2925	5396	54.2
Reactome	EnrichNet	17	116	14.7	443	728	**60.8**
	g:Profiler	685	2992	22.9	7137	15866	45.0
	GNNenrich(10)	1079	3901	**27.7**	5267	11403	46.2
	GNNenrich(40)	1088	3974	**27.4**	7752	17462	44.4

referenced in significant pathways.
The analysis of hub redundancy across the three methods does not single out GNNenrich as an outlier. Moreover, although the number of genes and pathways identified by GNNenrich is higher than that reported by EnrichNET and g:Profiler, both the total number of genes and the level of hub redundancy remain comparable to the results produced by g:Profiler.
This observation holds true even for lower values of β (e.g., $\beta = 10$), which favor the influence of interactions in enrichment detection. Interestingly, in the GC use case—which includes the highest number of hub genes—we did not observe a greater deviation in hub redundancy. The most notable differences were observed in the PD use case; nevertheless, hub redundancy remained similar between g:Profiler and GNNEnrich, indicating a limited overrepresentation of hub genes specifically influencing GNNEnrich's results. Furthermore, comparing the hub redundancy observed in these results with that derived from the complete set of the three databases. Table 39 reveals no substantial increase in the ratio in the GNNenrich results, thereby reinforcing the notion of a limited influence exerted by hub genes.

Database	Entries	Hub Occurrences	Hub Not Found	Hub redundancy
WikiPathways	32,368	12,822	855	0.3961
KEGG	12,982	4,922	1,084	0.3791
Reactome	96,917	38,957	255	0.4020

Table 39. Summary of hub gene occurrences in pathway databases. It reports the total number of gene entries (with duplicates), the number of occurrences of hub genes within the pathways, the number of hub genes not found in any pathway, and the hub redundancy relative to the total pathway gene entries

Investigation of the Influence of Hub Genes in Pathways Uniquely Identified by GNNenrich

Finally, we investigated whether the higher number of pathways specifically identified by GNNenrich were predominantly driven by PPI related to hub genes. For each pathway specifically detected by GNNenrich, we examined the organization of PPI between our query set and the corresponding enriched pathways. We selected only the proteins presenting PPI between the query and the detected pathways. Here, the arising idea is to check whether these proteins are hubs or not. To this end, we plot the distribution of their PPIs degree referenced in the STRING database. For comparison, the same approach was applied to pathways commonly detected by GNNenrich and g:Profiler and pathway specifically detected by g:Profiler.
Additionally, histograms are overlaid with the distribution of interaction degree distribution from all proteins referenced in the STRING v11 database. Figure 14a shows the degree distribution of proteins corresponding to genes jointly identified by GNNenrich and g:Profiler in Parkinson's disease on WikiPathways. In contrast, Figure 14b displays the distribution for genes uniquely identified by GNNenrich on WikiPathways. Similar distributions are shown in Figure 15 for gastric cancer.
These representations depict that, even after the careful selection of genes/proteins involved in the interactions between the query and the enriched pathways, no substantial differences were observed between specifically detected pathways and those shared across methods. Notably, the distribution of interaction degree associated with genes involved in pathways specifically detected by g:Profiler on WikiPathways for gastric cancer exhibits a similar profile (figure 16; the analogous figure on Parkinson's disease is not shown as only two pathways appeared to be specifically significant for g:profiler, see Figure 5 in the main text).
Moreover, if we hypothesize that gene hubs were overrepresented in the specific pathways identified by the GNNenrich model, a pattern related to gene/protein exceeding the previously introduced hub threshold (above 142 PPIs) would have been expected. However, the graphs produced here do not show a pronounced shoulder in their distribution for hub proteins as defined in this study. Additionally, the distribution follows a similar trend observed in the STRING database, nevertheless associated with a slight shift relative to protein/gene with no available interaction described in the STRING database. This shift is globally observed for specific and shared pathways across methods. Additionally, this trend may be anticipated, given that GNNenrich models are based on interaction networks. Moreover, the fact that the trend is also observed for g:Profiler results (figure 16) can be explained by the enrichment of all pathways in hub genes (Table 39) .
Overall, the results of this study indicate that the initial assumption characterizing hub genes as a major source of bias underlying the foundation of GNNenrich methods appears to be unfounded.

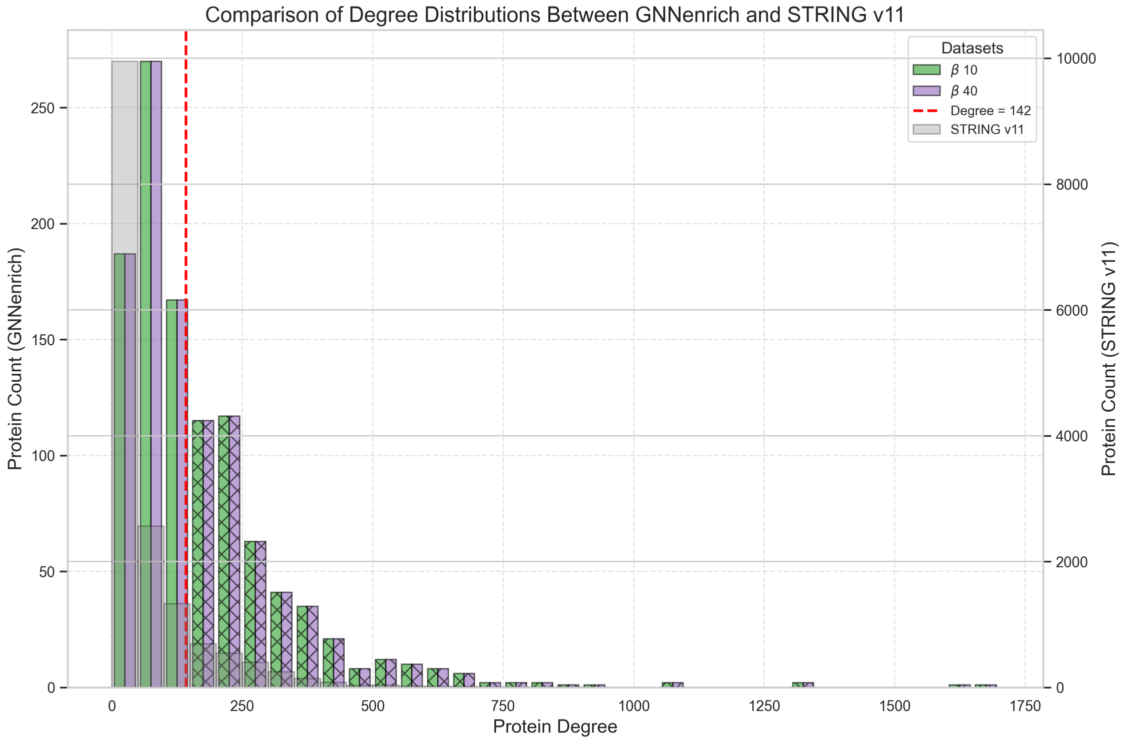

(a) Parkinson – Common results.

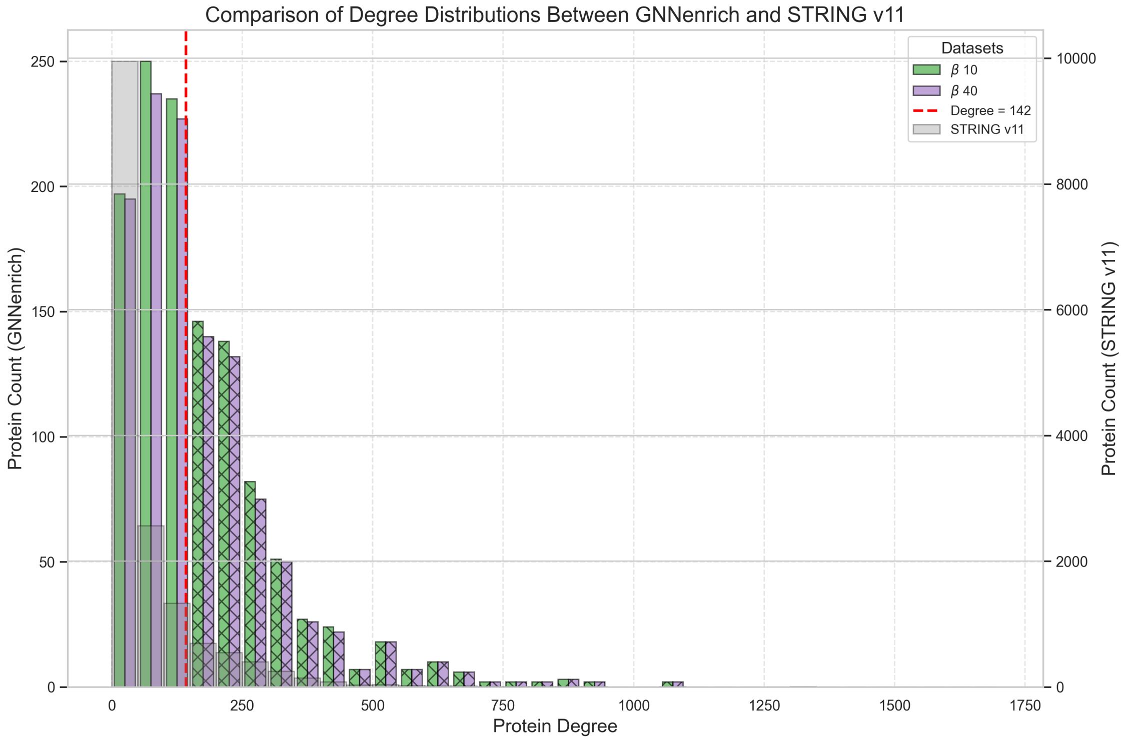

(b) Parkinson – GNNenrich-specific results.

Fig. 14: Comparison of protein degree distributions for genes identified by GNNenrich and g:Profiler for Parkinson's disease on WikiPathways. (a) Degree distribution of genes commonly identified by both tools, showing substantial overlap with high-degree (hub) proteins. (b) GNNenrich-specific genes, illustrating the unique enrichment patterns. In both panels, the STRING v11 background protein degree distribution is shown in gray, and the red dashed vertical line marks the hub threshold (degree = 142).

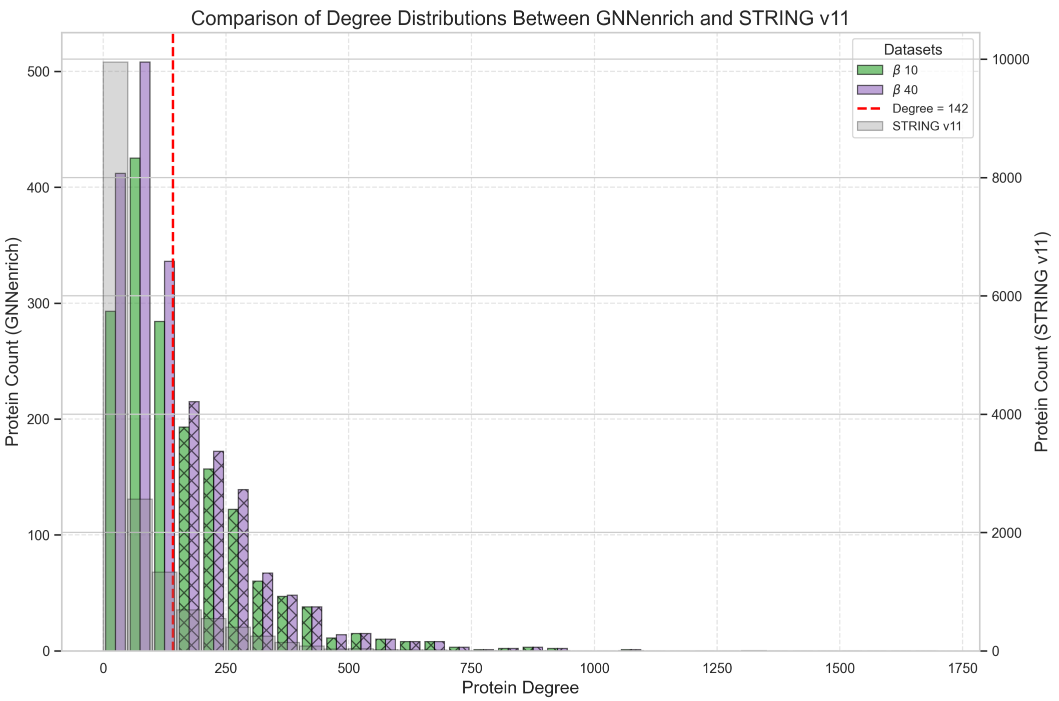

(a) Gastric cancer – Common results.

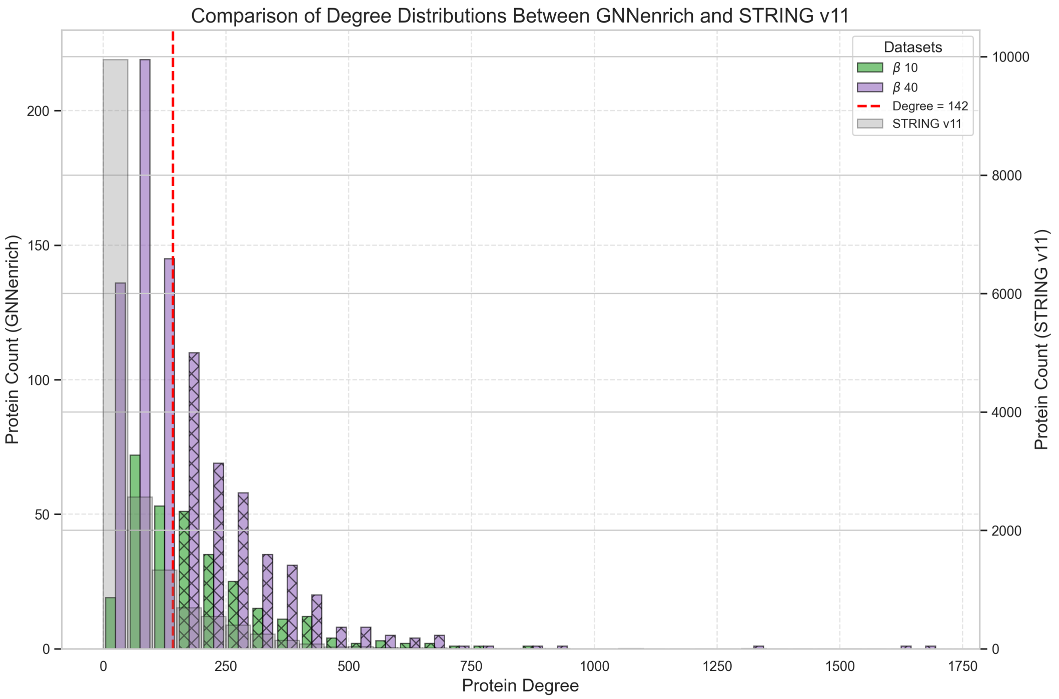

(b) Gastric cancer – GNNenrich-specific results.

Fig. 15: Protein degree distributions for genes identified by GNNenrich and g:Profiler for gastric cancer on WikiPathways. (a) Common gene set shows moderate enrichment in hub proteins. (b) GNNenrich-specific genes highlight stronger hub enrichment, particularly at $\beta = 10$. Gray: STRING v11 background; red dashed line: hub threshold (degree = 142).

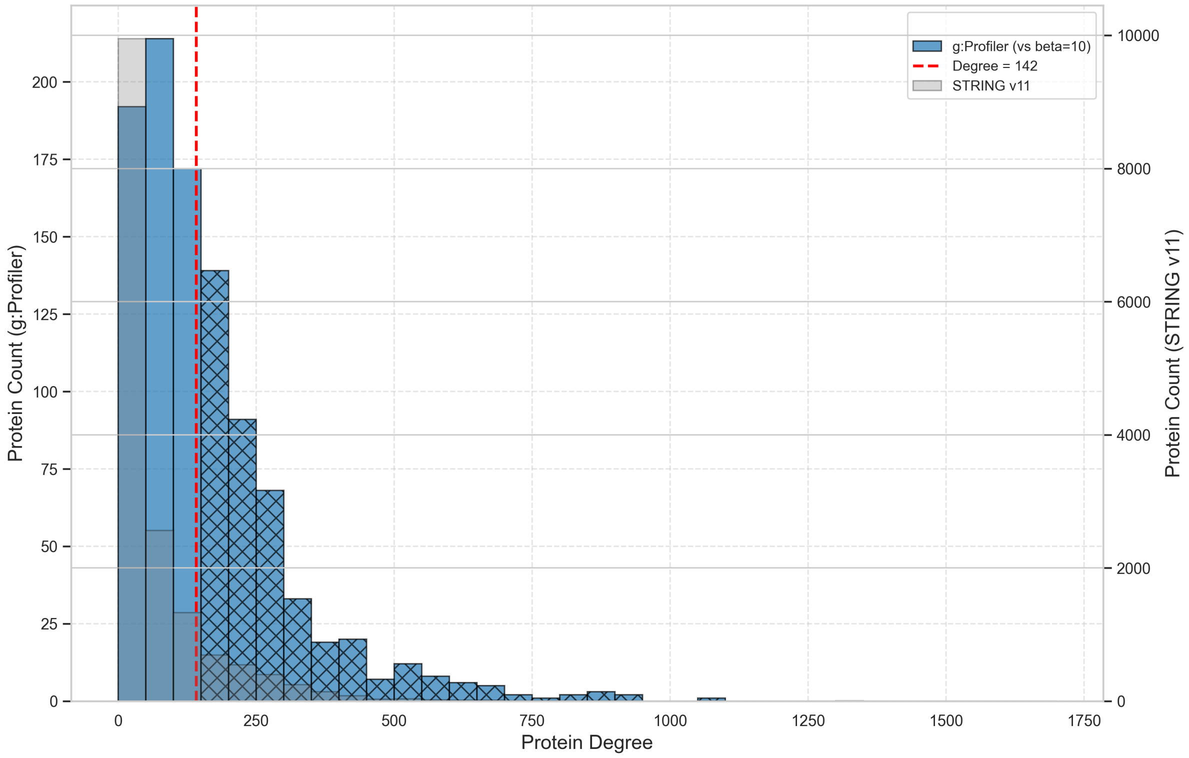

Fig. 16: Protein degree distribution for g:Profiler-specific genes in gastric cancer on WikiPathways. The pattern resembles that of GNNenrich-specific genes, with noticeable presence of hub proteins. Gray: STRING v11 background; red dashed line: hub threshold (degree = 142).

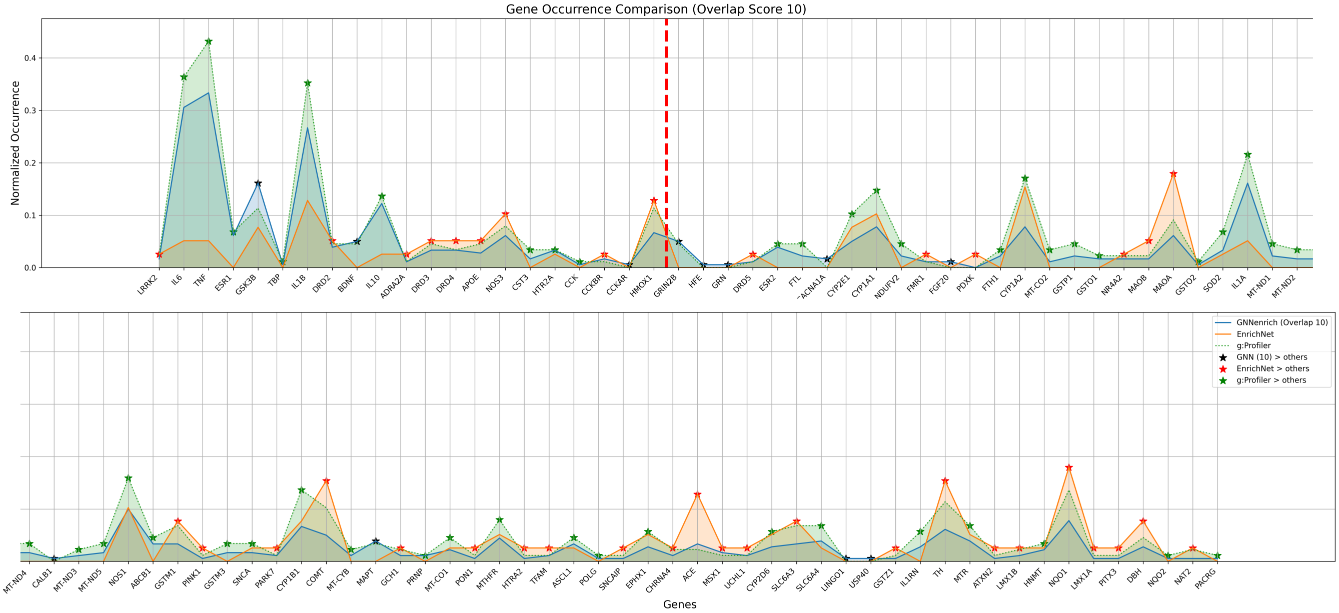

(a) Wikipathways/PD/overlap score = 10

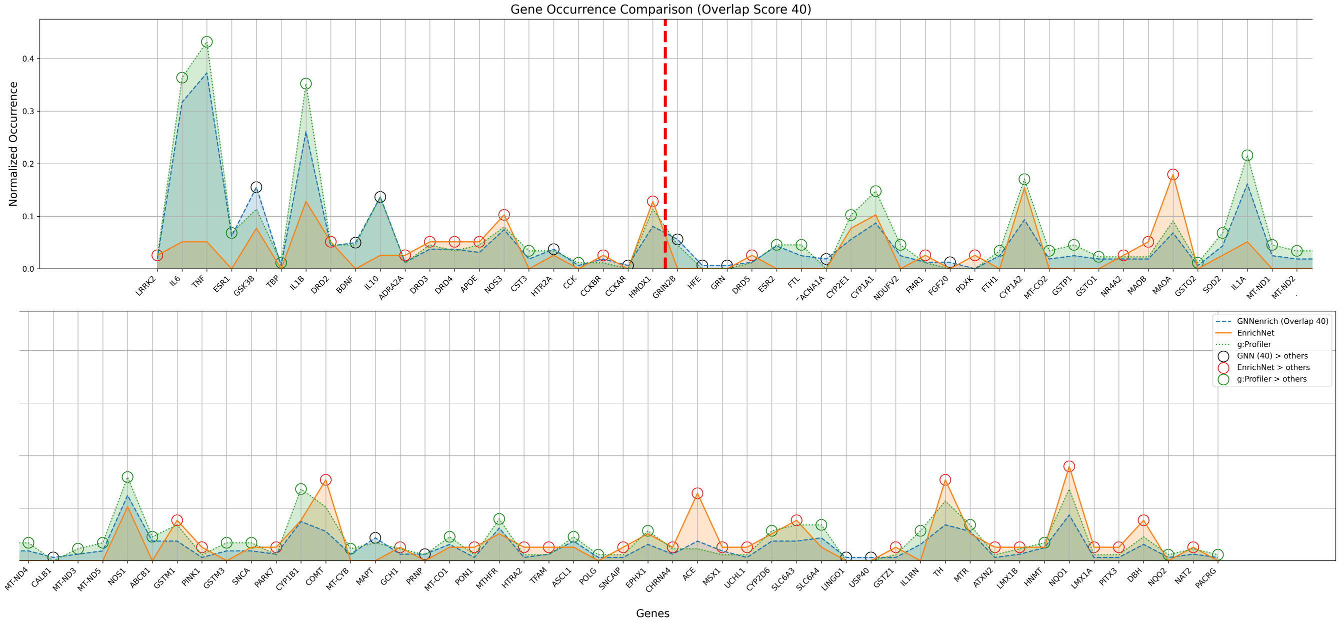

(b) Wikipathways/PD/overlap score = 40

Fig. 17: Normalized gene occurrences across three approaches: EnrichNet, g:Profiler, and GNN-based enrichment (GNNenrich), highlighting genes that are overrepresented in the enriched pathways. Subsets of genes for which each method yields dominant scores are quantified relative to the total number of genes associated with each method. The annotation box reports how many genes are prioritized more strongly by each method compared to the others, highlighting differences in gene prioritization patterns across the three enrichment strategies.

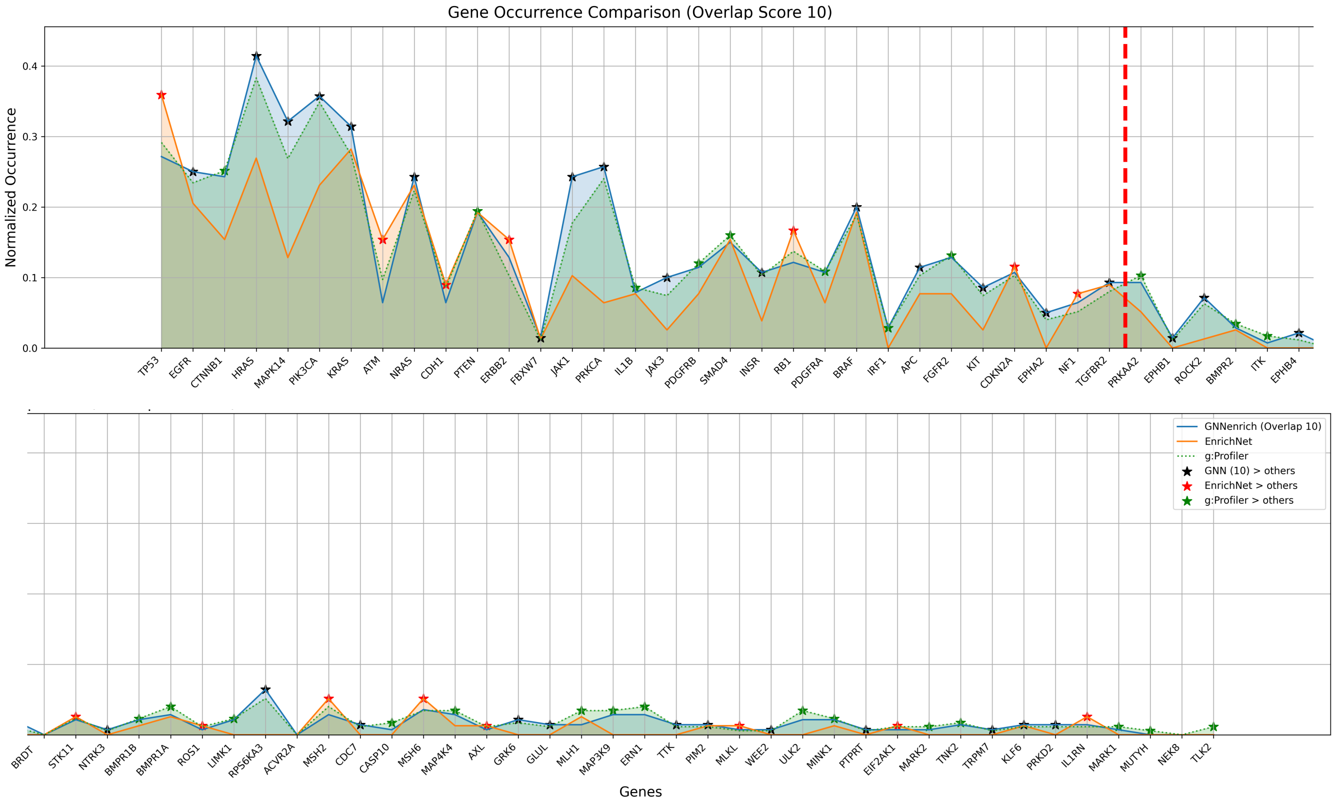

(a) Wikipathways/GC/overlap score = 10

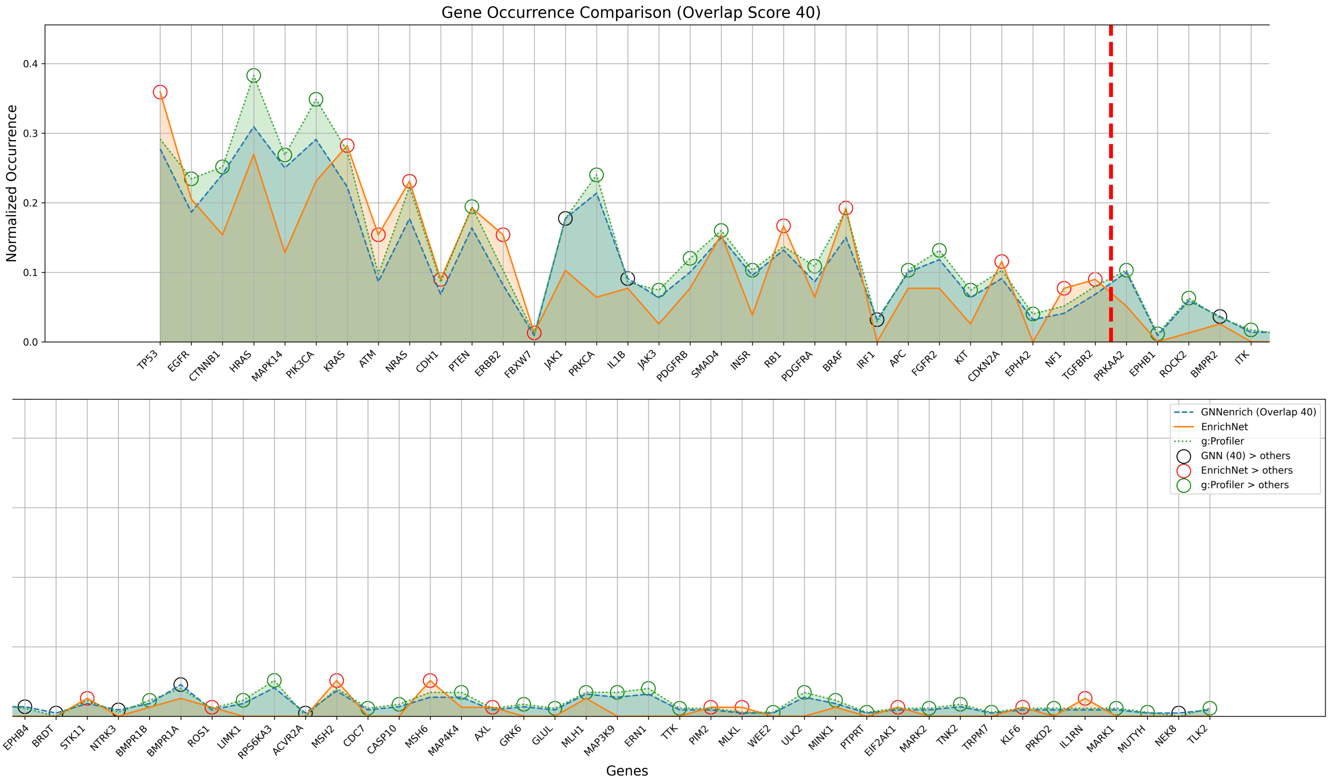

(b) Wikipathways/GC/overlap score = 40

Fig. 18: Normalized gene occurrences across three approaches: EnrichNet, g:Profiler, and GNN-based enrichment (GNNenrich), highlighting genes that are overrepresented in the enriched pathways.

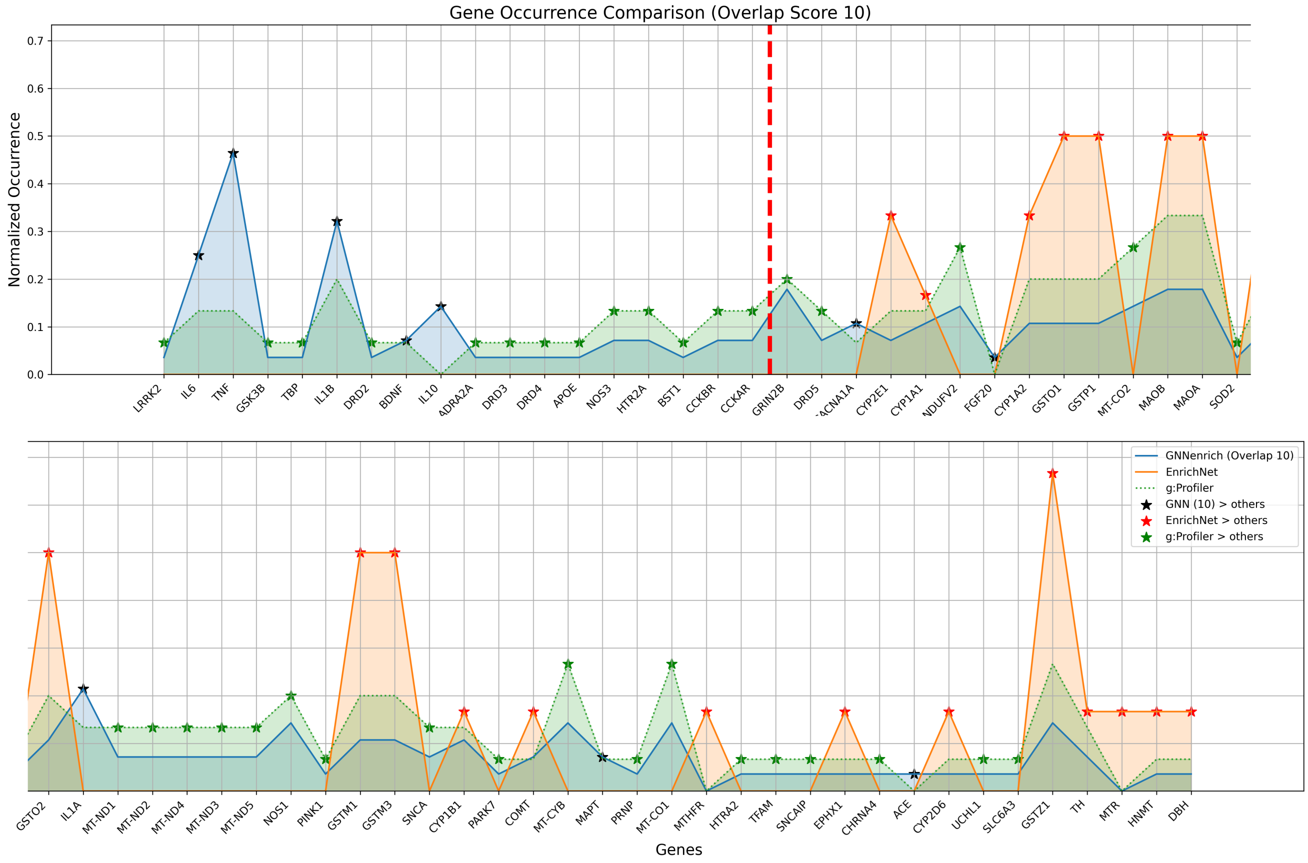

(a) KEGG/PD/overlap score = 10

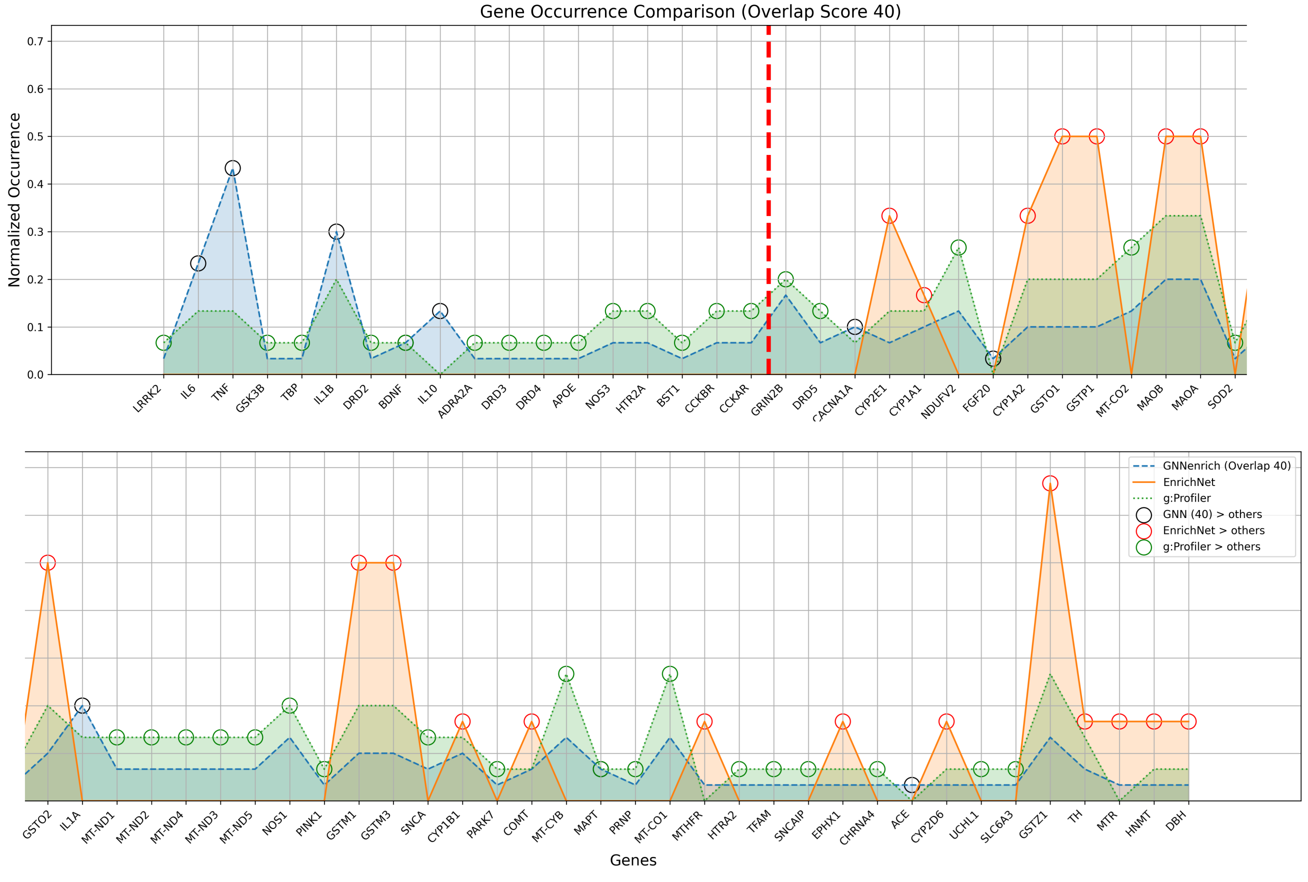

(b) KEGG/PD/overlap score = 40

Fig. 19: Normalized gene occurrences across three approaches: EnrichNet, g:Profiler, and GNN-based enrichment (GNNenrich), highlighting genes that are overrepresented in the enriched pathways.

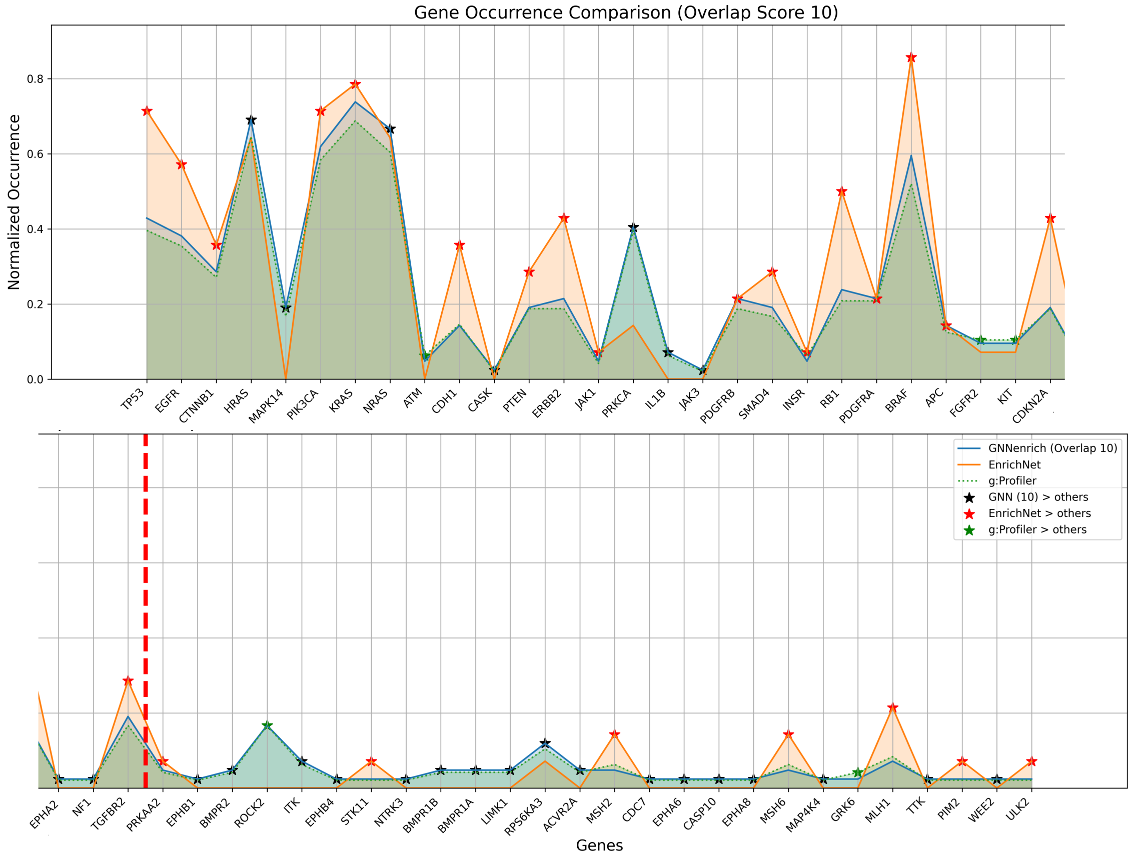

(a) KEGG/GC/overlap score = 10

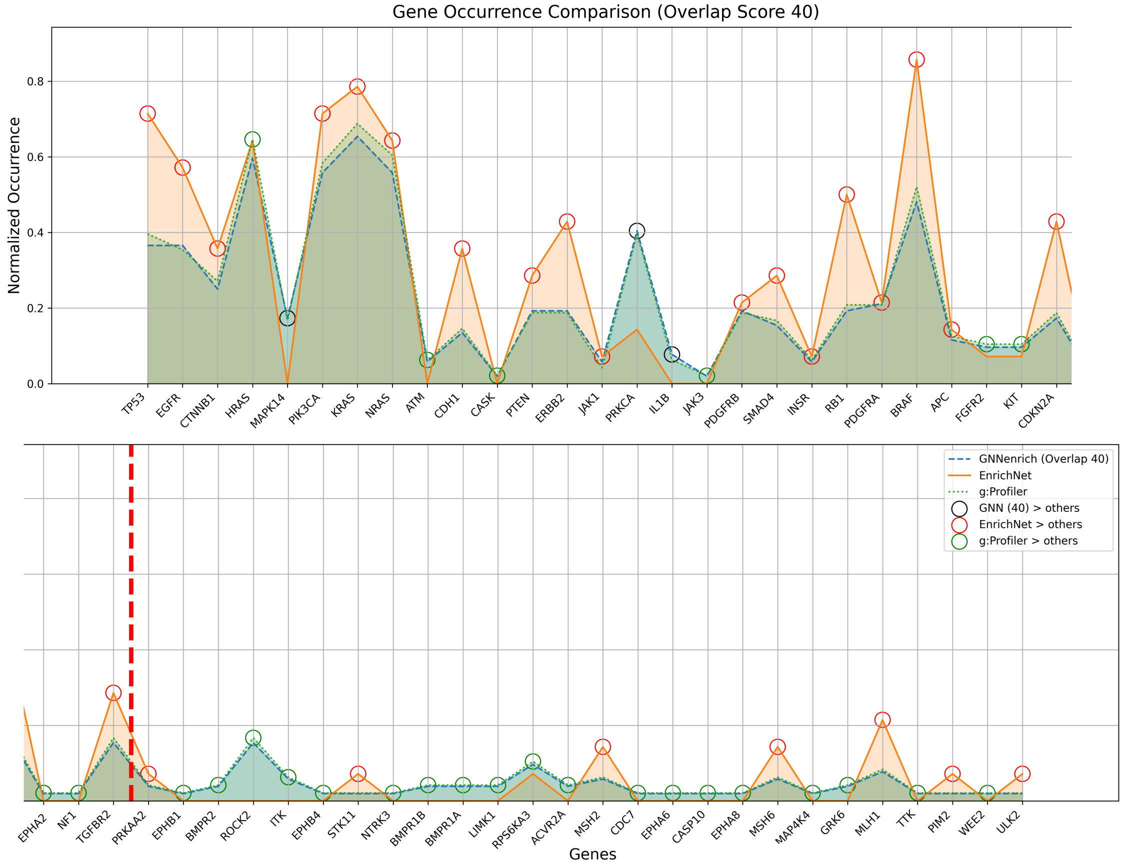

(b) KEGG/GC/overlap score = 40

Fig. 20: Normalized gene occurrences across three approaches: EnrichNet, g:Profiler, and GNN-based enrichment (GNNenrich), highlighting genes that are overrepresented in the enriched pathways.

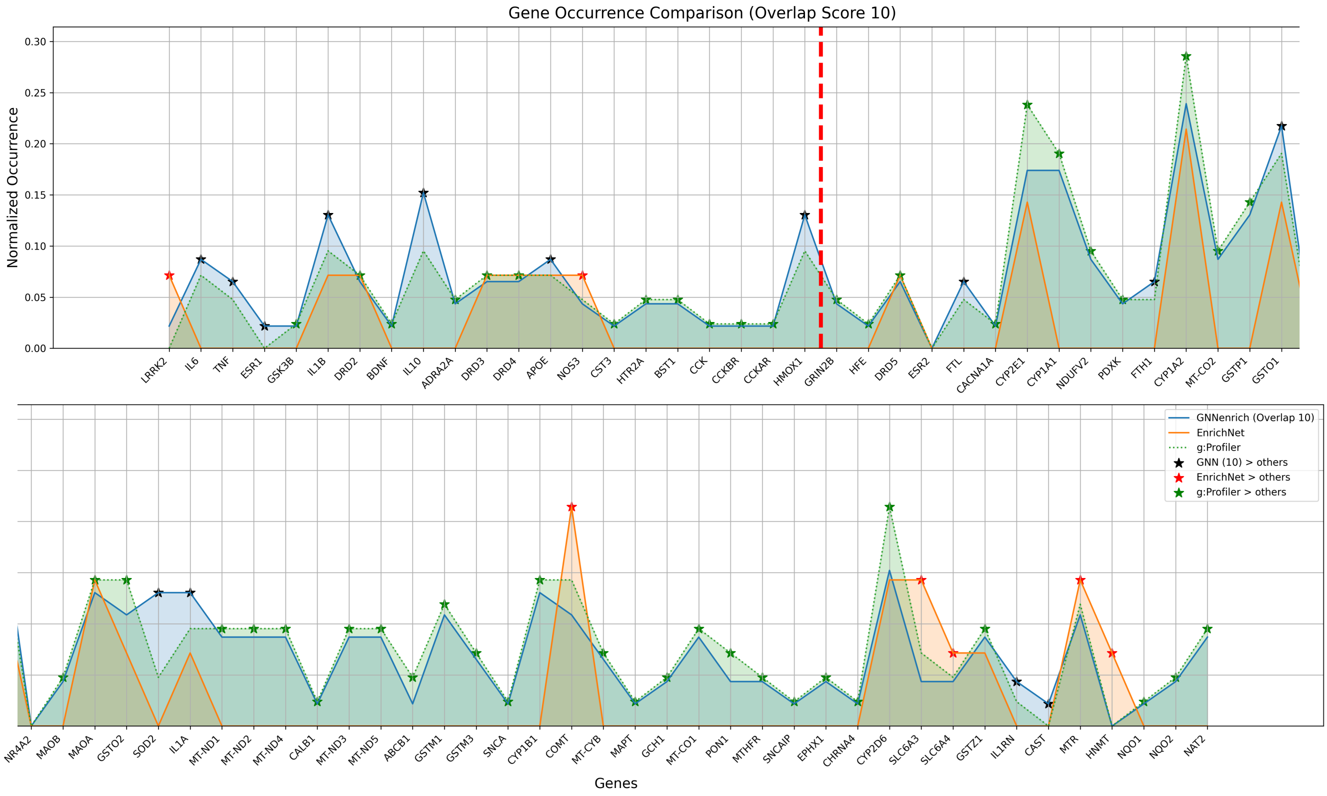

(a) Reactome/PD/overlap score = 10

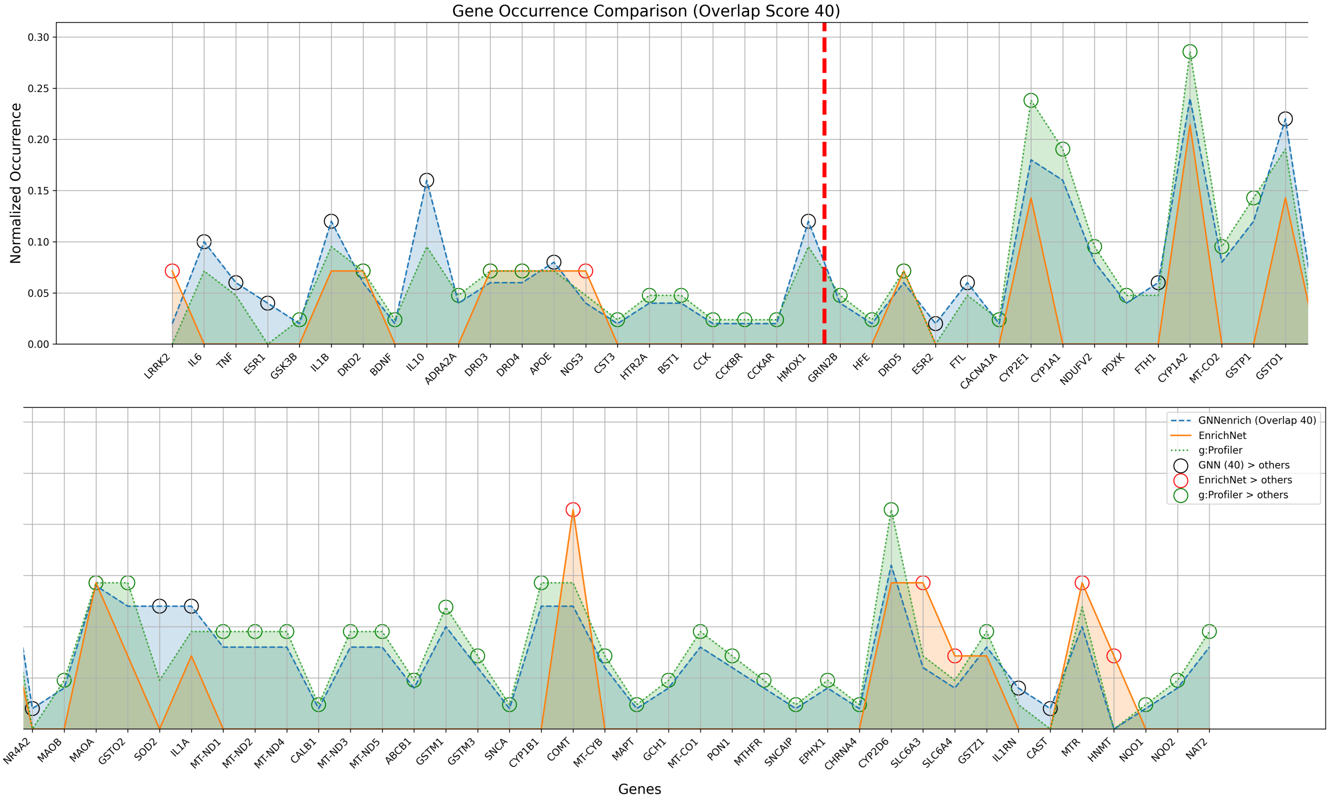

(b) Reactome/PD/overlap score = 40

Fig. 21: Normalized gene occurrences across three approaches: EnrichNet, g:Profiler, and GNN-based enrichment (GNNenrich), highlighting genes that are overrepresented in the enriched pathways.

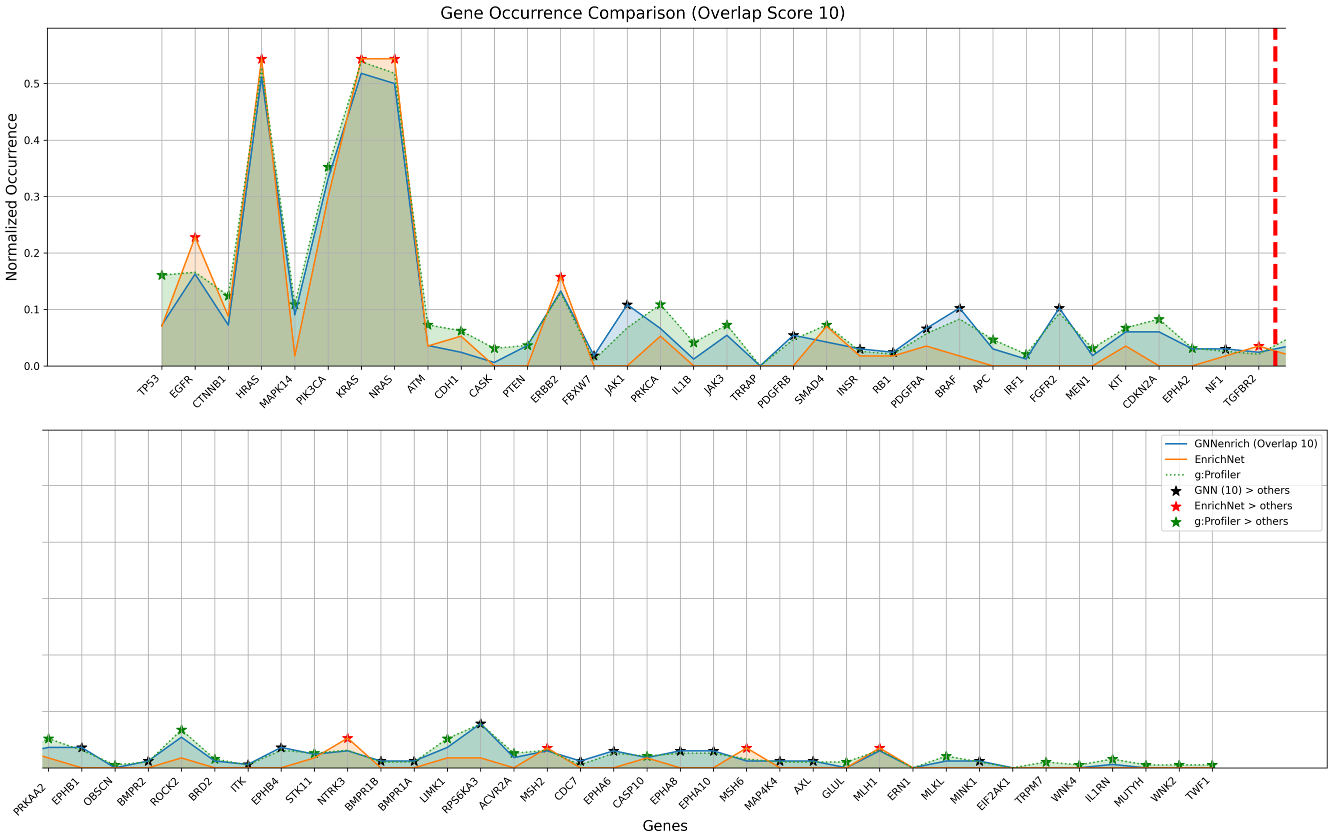

(a) Reactome/GC/overlap score = 10

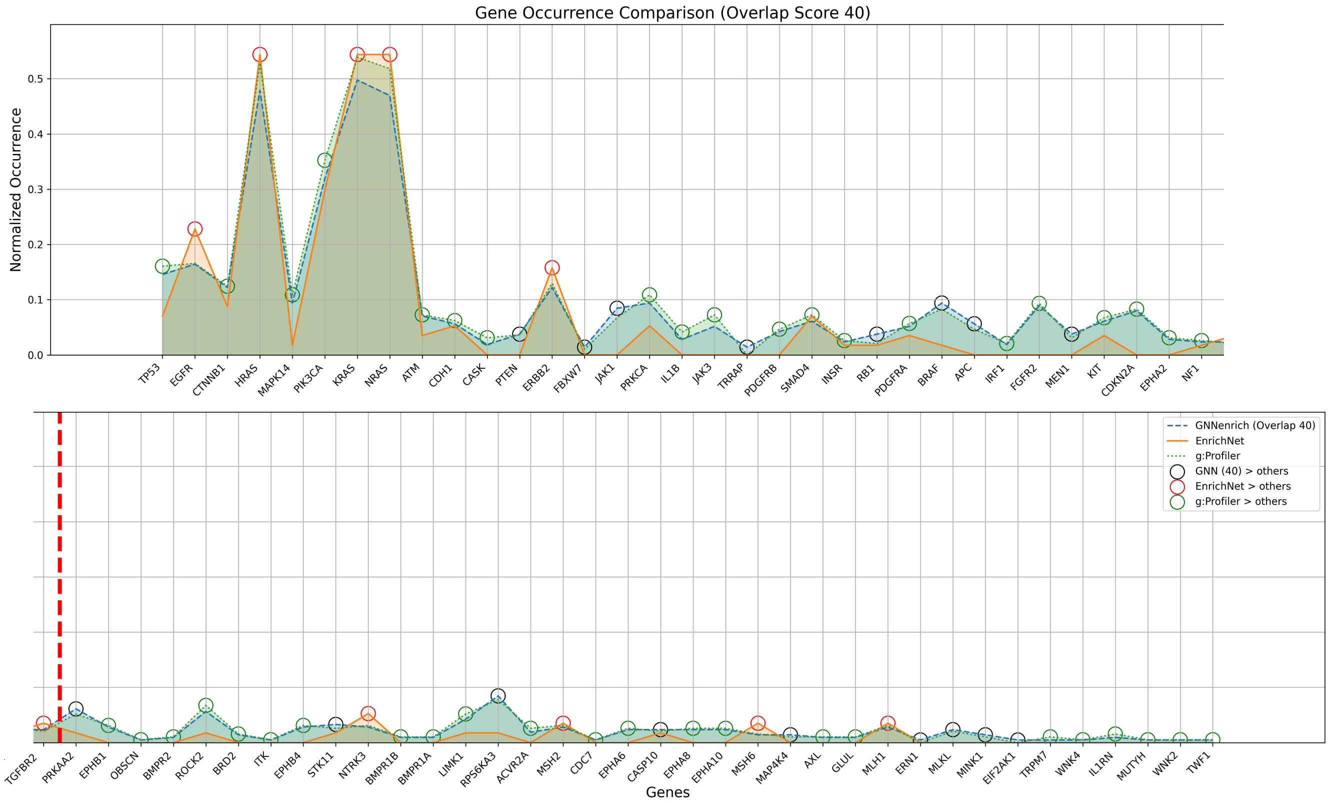

(b) Reactome/GC/overlap score = 40

Fig. 22: Normalized gene occurrences across three approaches: EnrichNet, g:Profiler, and GNN-based enrichment (GNNenrich), highlighting genes that are overrepresented in the enriched pathways.

20. Statistical significance of the Venn diagram

To assess the statistical significance of the overlaps shown in the Venn diagram, SuperExactTest [Wang et al. (2013) was used. Results are presented in 40 and 41.

Dataset	Combined Methods	p-value
	WikiPathways / Parkinson	
	g:Profiler & EnrichNet	1.47×10^{-15}
	GNNenrich & EnrichNet	1.94×10^{-12}
	GNNenrich & g:Profiler	4.22×10^{-58}
	GNNenrich & g:Profiler & EnrichNet	5.05×10^{-29}
	WikiPathways / Gastric cancer	
	g:Profiler & EnrichNet	1.50×10^{-20}
	GNNenrich & EnrichNet	7.54×10^{-19}
	GNNenrich & g:Profiler	1.31×10^{-51}
	GNNenrich & g:Profiler & EnrichNet	3.67×10^{-33}
	KEGG / Parkinson	
	g:Profiler & EnrichNet	1.02×10^{-8}
	GNNenrich & EnrichNet	3.30×10^{-7}
	GNNenrich & g:Profiler	5.97×10^{-24}
	GNNenrich & g:Profiler & EnrichNet	5.85×10^{-16}
	KEGG / Gastric cancer	
	g:Profiler & EnrichNet	1.27×10^{-9}
	GNNenrich & EnrichNet	9.72×10^{-9}
	GNNenrich & g:Profiler	1.61×10^{-35}
	GNNenrich & g:Profiler & EnrichNet	6.94×10^{-17}
	Reactome / Parkinson	
	g:Profiler & EnrichNet	1.95×10^{-10}
	GNNenrich & EnrichNet	7.41×10^{-7}
	GNNenrich & g:Profiler	6.84×10^{-59}
	GNNenrich & g:Profiler & EnrichNet	1.77×10^{-13}
	Reactome / Gastric cancer	
	g:Profiler & EnrichNet	6.12×10^{-34}
	GNNenrich & EnrichNet	1.08×10^{-26}
	GNN & g:Profiler	1.33×10^{-99}
	GNNenrich & g:Profiler & EnrichNet	1.18×10^{-58}

Table 40. P-values of significant overlaps between analysis methods (GNN, g:Profiler, EnrichNet) across six datasets: WikiPathways, KEGG, and Reactome for Parkinson's disease and gastric cancer with an overlap score $\beta = 10$. All p-values are highly significant ($p < 10^{-3}$).

Dataset	Combined Methods	p-value
	WikiPathways / Parkinson	
	g:Profiler & EnrichNet	1.47×10^{-15}
	GNNenrich & EnrichNet	2.53×10^{-16}
	GNNenrich & g:Profiler	3.31×10^{-69}
	GNNenrich & g:Profiler & EnrichNet	3.16×10^{-32}
	WikiPathways / Gastric cancer	
	g:Profiler & EnrichNet	1.50×10^{-20}
	GNNenrich & EnrichNet	2.52×10^{-26}
	GNNenrich & g:Profiler	6.96×10^{-127}
	GNNenrich & g:Profiler & EnrichNet	4.61×10^{-50}
	KEGG / Parkinson	
	g:Profiler & EnrichNet	1.00×10^{-5}
	GNNenrich & EnrichNet	1.16×10^{-5}
	GNNenrich & g:Profiler	3.57×10^{-14}
	GNNenrich & g:Profiler & EnrichNet	8.72×10^{-10}
	KEGG / Gastric cancer	
	g:Profiler & EnrichNet	1.27×10^{-9}
	GNNenrich & EnrichNet	4.64×10^{-9}
	GNNenrich & g:Profiler	4.09×10^{-40}
	GNNenrich & g:Profiler & EnrichNet	5.88×10^{-18}
	Reactome / Parkinson	
	g:Profiler & EnrichNet	1.95×10^{-10}
	GNNenrich & EnrichNet	1.47×10^{-11}
	GNNenrich & g:Profiler	2.53×10^{-72}
	GNNenrich & g:Profiler & EnrichNet	7.14×10^{-23}
	Reactome / Gastric cancer	
	g:Profiler & EnrichNet	6.12×10^{-34}
	GNNenrich & EnrichNet	1.44×10^{-36}
	GNNenrich & g:Profiler	1.01×10^{-181}
	GNNenrich & g:Profiler & EnrichNet	6.49×10^{-76}

Table 41. P-values of significant overlaps between combinations of methods (GNN, g:Profiler, EnrichNet) across WikiPathways, KEGG, and Reactome datasets for Parkinson's disease and gastric cancer with an overlap score $\beta = 40$. All p-values are highly significant ($p < 10^{-3}$).

21. Random permutations for sampling the null distribution

First, we would like to clarify that the random sampling is used to simulate random query lists and that the pathways remain the same at all time. Our use of the term permutations was probably misleading. To determine an appropriate number of random samplings for empirical p-value estimation, we tested several settings: 100, 1,000, 10,000, and 100,000 random samplings.
The figures (23a) and 23b illustrates the null distributions obtained from 100 and 1,000 random samplings. As shown, both distributions exhibit considerable variability and lack stability, especially with only 100 random samplings, indicating that these settings are insufficient for reliable inference.
Based on these observations, we increased the number of random samplings to 10,000 where the null distribution became smoother and more stable. Using 10,000 random samplings ensures a fine-grained estimation, with a minimum possible p-value of 0.0001, and provides a stable and reproducible approximation of statistical significance (figure 24). This level of resolution is generally sufficient to distinguish between noise and meaningful signal. Moreover, visual inspection of the null distribution confirmed its smoothness, indicating that 10,000 random samplings can be an effective balance between computational cost and statistical accuracy.

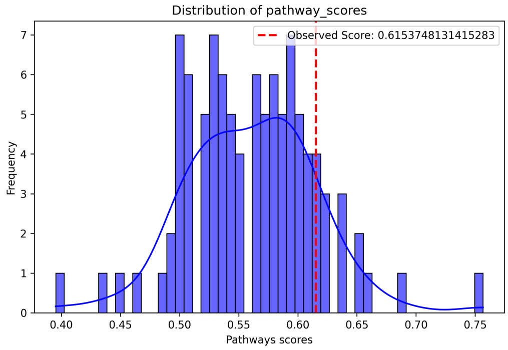

(a) 100 random samplings

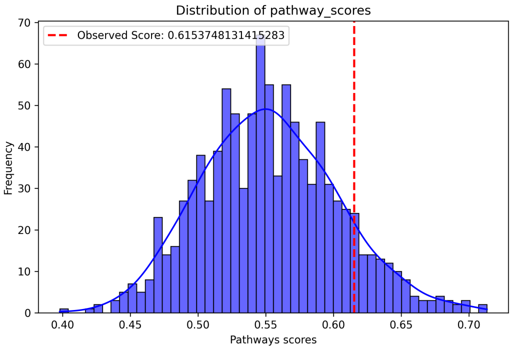

(b) 1,000 random samplings

Fig. 23: Comparison of null distributions for pathway enrichment scores using increasing numbers of random samplings (100 and 1,000). Each histogram shows the distribution under the null hypothesis from randomly sampled gene sets. As the number of random samplings increases, the distribution becomes smoother and more stable, leading to more precise p-value estimation. The vertical dashed line indicates the observed enrichment score.

While a resolution of 10,000 random samplings allows for the estimation of p-values as low as 0.0001, we observed that multiple pathways reached this minimum threshold in the GNNenrich results. This limited our ability to distinguish between highly significant pathways based on their degree of enrichment. To address this, we increased the number of random samplings to 100,000, allowing for finer granularity in p-value estimation (down to 1e-5). This higher resolution enabled us to better differentiate pathways with similar but distinct enrichment levels, thereby improving the interpretability and ranking of biologically relevant signals.
Despite the relatively low number of interactions with the gene list of interest, certain pathways exhibit a well-defined null hypothesis distribution. For instance, the Alanine Aspartate and Glutamate Metabolism pathway shows 28 interactions, including 12 that do not involve GLUL, a gene shared with the gastric cancer-associated list (Figure 25a). Similarly, the Alpha Linolenic Acid Metabolism pathway (Figure 25b), with only 8 interactions and no overlapping genes, and the Aminoacyl tRNA Biosynthesis pathway (Figure 25c), with just 3 interactions, both display a Gaussian-shaped null distribution. This tends to show that the low number of interactions has no impact on the shape of the null distribution, which remains stable and suitable for statistical interpretation.
We can also observed for cases associated to pathways with a high number of interaction gaussian-like null distributions. For example, we can mention The Apoptosis pathway (Figure 26a) displays 88 genes, with 5 overlapping genes from the gastric cancer list. This pathway shows 513 protein–protein interactions (PPIs) with the list, 330 of which involve non-overlapping genes, highlighting the importance of indirect connectivity. We may observe similar trends for pathways like the Axon Guidance pathway (Figure 26b) exhibiting 680 PPIs and includes 4 overlapping genes or The Bladder Cancer pathway (Figure 26c) with 10 genes shared with the gastric cancer list and 478 total interactions.
In a whole, the null hypothesis obtained from 100,000 random gene lists are closely approximated by a Gaussian, confirming that the statistical behavior remains robust regardless of the number of overlapping genes or the total number of interactions.

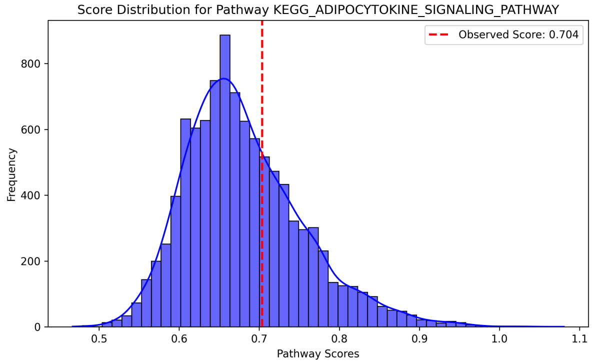

(a) Adipocytokine signaling pathway

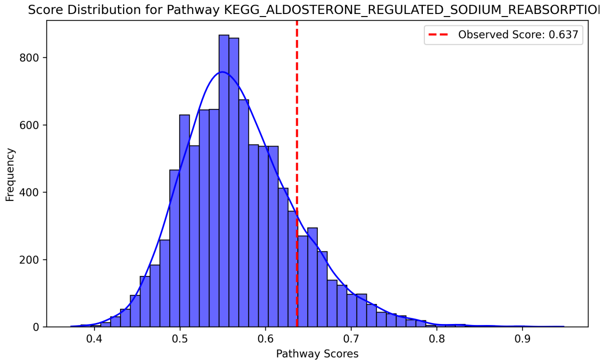

(b) Aldosterone-regulated sodium reabsorption

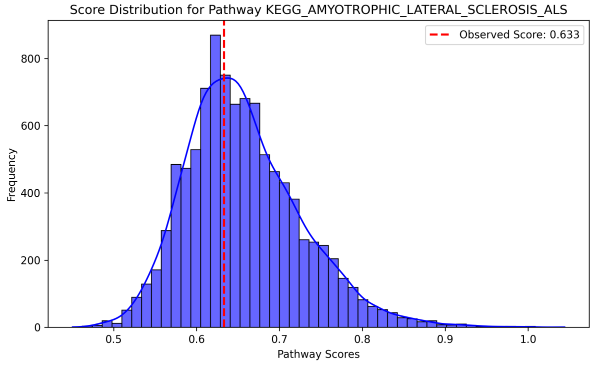

(c) Amyotrophic lateral sclerosis (ALS)

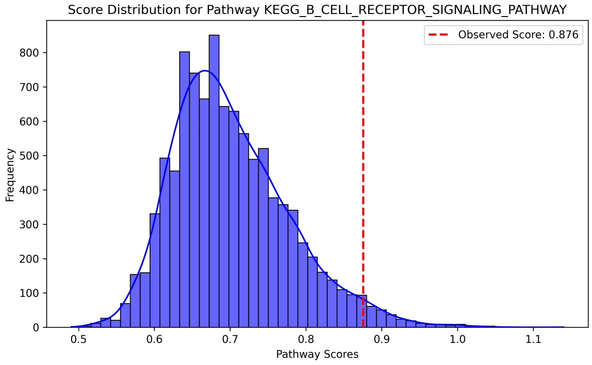

(d) B cell receptor signaling pathway

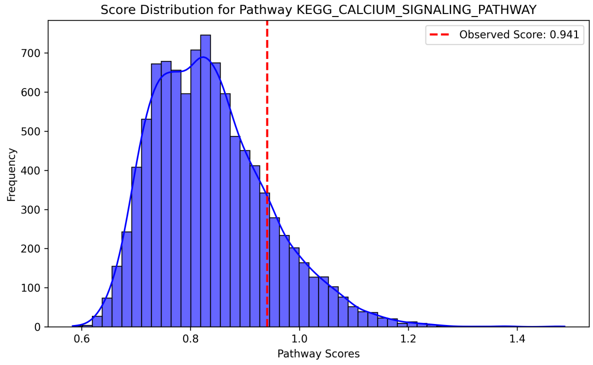

(e) Calcium signaling pathway

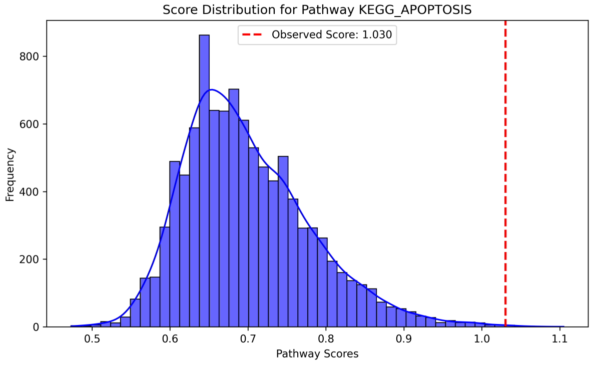

(f) Apoptosis

Fig. 24: Null distributions of enrichment scores for six KEGG pathways using 10,000 random samplings on the GC gene list. Each histogram shows the empirical null distribution. The vertical dashed line represents the observed enrichment score. The GNNenrich method detects significant enrichment when the score lies at the tail of the distribution.

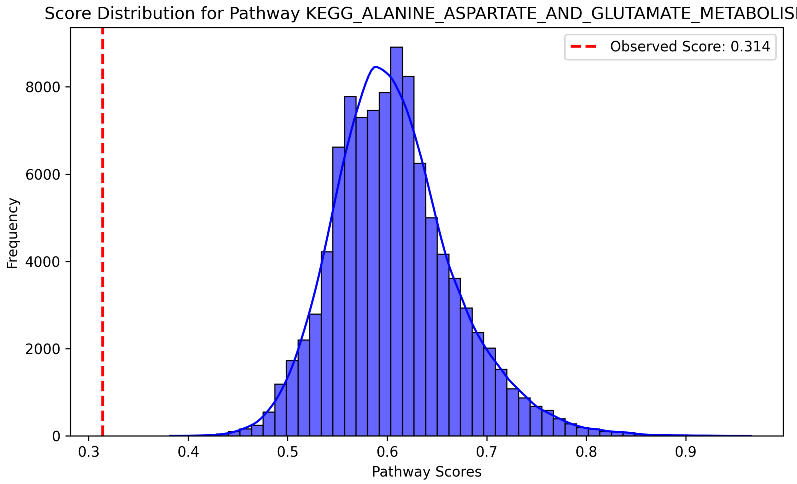

(a) Alanine, Aspartate and Glutamate Metabolism

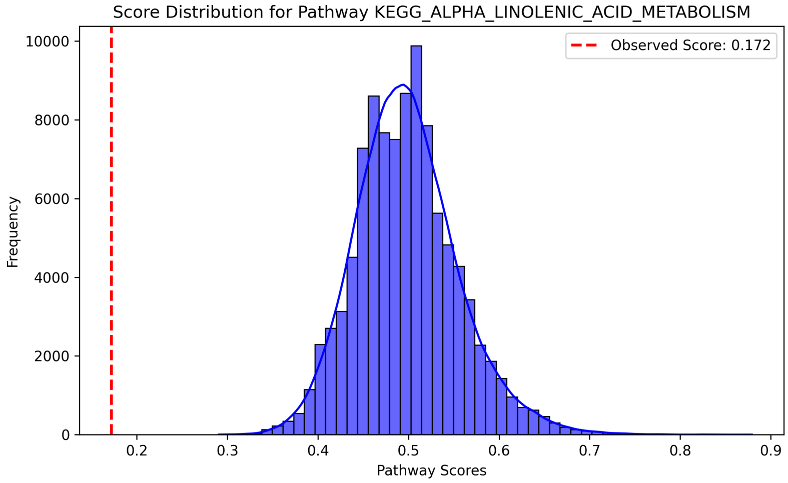

(b) Alpha Linolenic Acid Metabolism

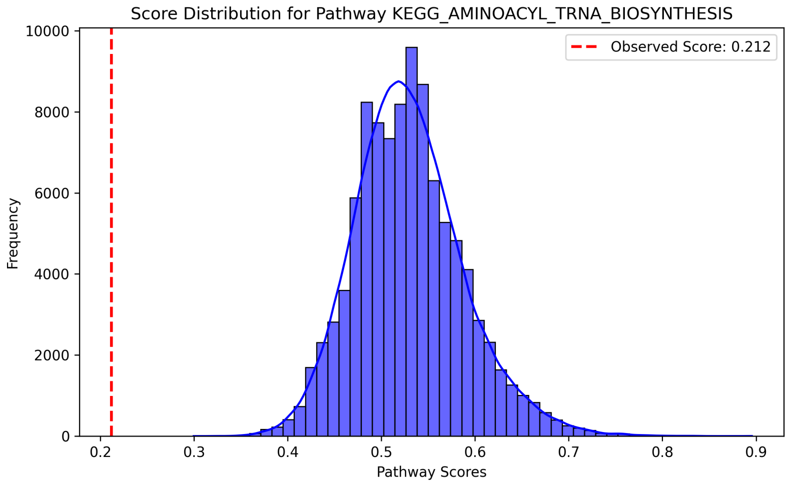

(c) Aminoacyl tRNA Biosynthesis

Fig. 25: Null distributions of pathway enrichment scores based on 100,000 random samplings for three KEGG pathways with few interactions with the query. Despite low interaction counts, all three distributions exhibit Gaussian-like shapes, indicating statistical behavior under the null is preserved.

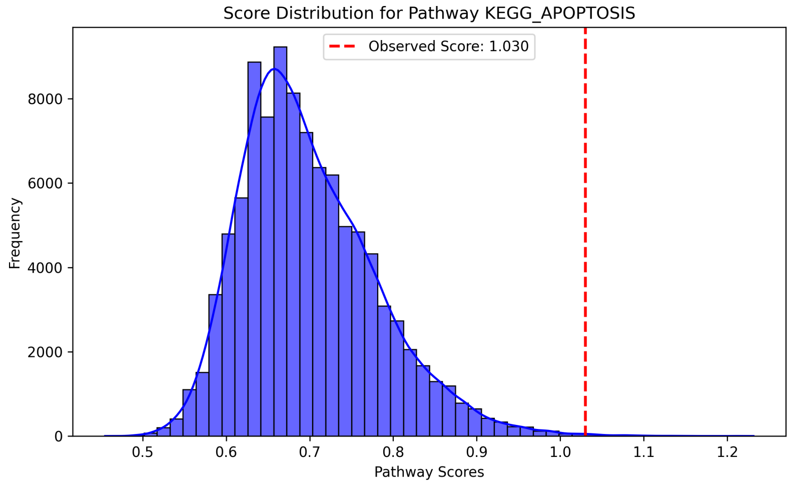

(a) Apoptosis (5 genes, 513 interactions)

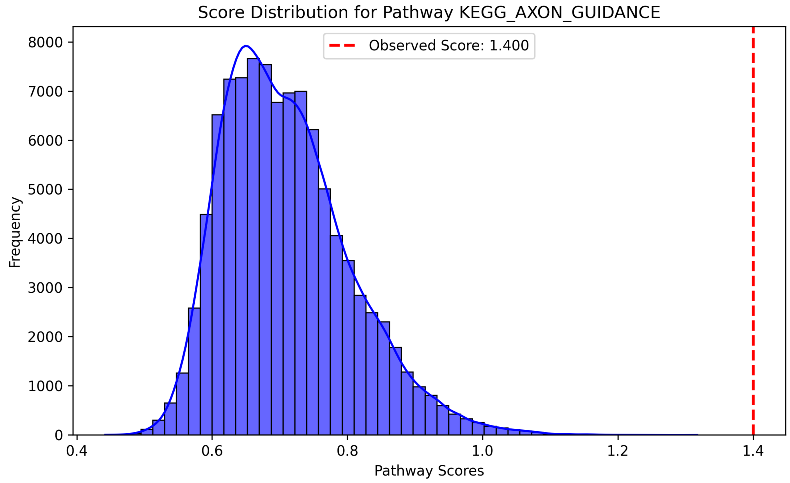

(b) Axon Guidance (4 genes, 680 interactions)

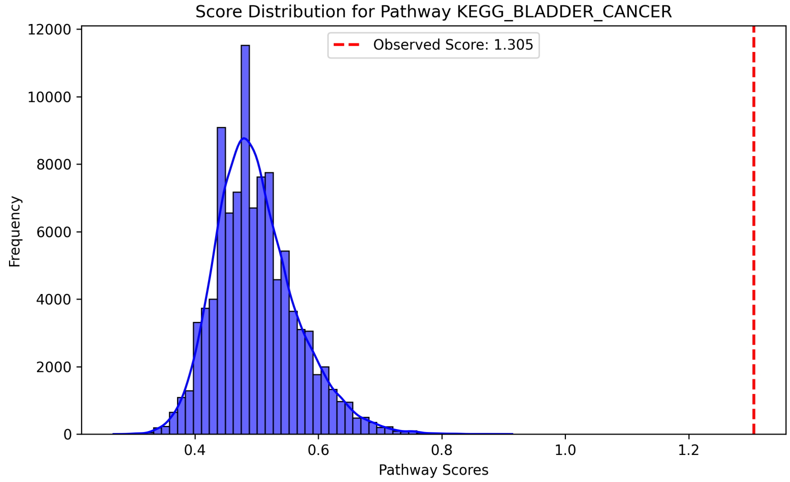

(c) Bladder Cancer (10 genes, 478 interactions)

Fig. 26: Null distributions of enrichment scores based on 100,000 random samplings for three significantly enriched KEGG pathways. Each plot shows a clear separation from the bulk of the null, indicating robust statistical enrichment.

22. Computational efficiency and scalability of GNNenrich

GNNenrich consists of two main components: a first phase to train a deep learning model based on a GNN using the STRING database and a second phase dedicated to inference for enrichment analysis.
The training phase, which involves generating protein encoding requires GPUs and dedicated computational resources. Bayesian optimization was performed on compute nodes equipped with NVIDIA A100 GPUs. The GNN training was repeated six times using embedding sizes selected through the optimization process. The durations of these training runs are reported in the table 42. Once generated, these protein encodings are then used in the enrichment analysis phase.

Embedding Size	Duration
100	3h 25m
748	3h 35m
475	3h 53m
372	3h 27m
183	3h 59m
232	3h 57m

Table 42. For each embedding size, the total training duration is reported. These durations correspond to selected runs performed during Bayesian optimization. A global training duration of 1,336 minutes (approximately 22h 16m), and an average duration per embedding of 223 minutes (3h 43m).

During this second phase, enrichment scores are computed between biological pathways and a given query or a random list. This operation is repeated $T * Q$ times, where T is the number of pathways in the annotation database and Q is the number of random sampling. In the following, we analyze the behavior of GNNenrich with respect to these two factors.
The execution time of GNNenrich is primarily influenced by the number of random samplings and the size of the annotation database (i.e., the number of pathways), while the choice of β has no impact on runtime. GNNenrich was run on the high-performance computing (HPC) infrastructure, using 72 CPU cores for all tests. Each compute node is equipped with *Intel(R) Xeon(R) Gold 6338 CPUs @ 2.00GHz*. These CPUs use the `x86_64` architecture. The results, summarized in Table 43, show that the runtime increases approximately linearly with the number of random samplings.
To reduce computation time in repeated analyses, random sampling can be cached rather than regenerated for each run. This optimization is implemented in the demo provided in the GitLab project and significantly improves performance.
In future versions of GNNenrich, especially when deployed as an API, we plan to precompute and store these random lists on the server side.
We also assessed the empirical impact of the number of random samplings on the final results. For the KEGG database, the set of significant pathways remained stable between 10,000 and 100,000 samplings (28 pathways identified in both cases), with only minor variation at 1,000 samplings (2 additional pathways). For WikiPathways, 178 pathways were identified as significant at 100,000 samplings, of which 174 were already found with both 10,000 and 1,000 samplings. These results suggest that 10,000 samplings already provide stable and reliable enrichment outcomes for practical use.

Use Case	Number of random samplings	CPU Cores	Execution Time
	1,000,000	72	$> 10h$
WikiPathways	100,000	72	1h 50min
	10,000	72	12min 39s
	1,000	72	2min 12s
	1,000,000	72	6h 54 min 37s
KEGG	100,000	72	44min 14s
	10,000	72	5min 26s
	1,000	72	1min 24s

Table 43. Execution time of GNNenrich for analyses on WikiPathways and KEGG

23. Overlap-free GNNenrich results

MalaCards as a comprehensive database of human diseases, provides integrated information on disease-gene associations, biological pathways, clinical trials, medications, and phenotypes.

Using MalaCards, we explore gene associations with Parkinson's disease and Gastric cancer, focusing on pathways specifically identified by GNNenrich with any overlap between the initial query and the pathway detected (Table 6).

Some of these pathways specific to GNNenrich have a high number of genes in common with the MalaCards list, compared to the total number of genes they contain. This means they are likely to be functionally related, even if they don't overlap with the original queries. For example, "SCFA and skeletal muscle substrate metabolism pathway" is entirely composed (ratio = 1) of genes found in the PD-associated list from MalaCards, suggesting a potential association with this neurodegenerative disease. In gastric cancer, the "Osteopontin signaling" pathway exhibits a high ratio (0.85), indicating strong representation of pro-inflammatory and pro-metastatic axes involving key players such as SPP1, RELA, and MAP kinases. Similarly, the "PTK6 regulates cell cycle" pathway (ratio = 0.83) is enriched in the Reactome predictions (β = 10), reflecting the engagement of major cell cycle regulators (e.g., CDK2, CDK4, CCND1) in tumor proliferation mechanisms. These findings support the idea that the integration of protein-protein interaction (PPI) networks enables the identification of biologically significant disease associations, beyond direct gene overlap. Among the specific pathways with zero overlap highlighted by GNNenrich (β = 10), the pathways "Removal of aminoterminal propeptides from gamma-carboxylated proteins" and "Gamma-carboxylation, transport and amino-terminal cleavage of proteins" were identified as significant. Despite showing no direct overlap with the Parkinson's disease gene list from MalaCards, their enrichment suggests potential indirect or unexplored functional associations. According to the references in table 50, the genes involved in these two gamma-carboxylation-related pathways are associated not only with Parkinson's disease, but also more broadly with neurodegenerative disorders. This supports the potential functional relevance of these pathways, despite their absence from conventional gene lists used in standard enrichment analyses.

Table 6, Table 17, Table 22, Table 23, Table 32, Table 33 have been updated with blue text.

Selection of significant pathways specific to GNNenrich with $\beta = 10$ for the PD use case on WikiPathways.

Pathway Name	BH	Size	Overlap	PPI	Gene annotated by Malacards
OSTEOBLAST SIGNALING	1.22E-02	14	0	35	PDGFB and PDGFRB are related to Parkinson's disease
OSTEOCLAST SIGNALING	1.51E-02	16	0	40	AIMP2, IFNB1, MAPK8, PDGFB, SPP1
SCFA AND SKELETAL MUSCLE SUBSTRATE METABOLISM	1.90E-02	6	0	24	PPARD, FFAR2, FFAR3, GCG, PYY, SLC2A4
BMP2WNT4FOXO1 PATHWAY IN PRIMARY ENDOMETRIAL STROMAL CELL DIFFERENTIATION	2.29E-02	13	0	35	BCL2L11, BMP2, CTNNB1, DKK1, FOXO1, SST
LET7 INHIBITION OF ES CELL REPROGRAMMING	4.49E-02	15	0	22	EGR1, KLF4, MIRLET7C, MIRLET7D, MIRLET7G
OSX AND MIRNAS IN TOOTH DEVELOPMENT	4.56E-02	34	0	32	CTNNB1, DKK1, KLF4, MIR145, MIR204, MIR200
ANGIOTENSIN II RECEPTOR TYPE 1 PATHWAY	4.65E-02	28	0	95	ACE2, AGT, AGTR1, HIF1A, MAPK1, NOX4
CALORIC RESTRICTION AND AGING	4.75E-02	8	0	77	AKT1, IGF1, MTOR, NAMPT, PPARGC1A
BMP SIGNALING IN EYELID DEVELOPMENT	4.82E-02	20	0	80	BMP4, EGFR, FGF10, JUN, MAP3K1, MAP2K1
MICRORNA NETWORK ASSOCIATED WITH CHRONIC LYMPHOCYTIC LEUKEMIA	4.86E-02	10	0	26	BCL2, MCL1, MIR34A, MIR34B, MIR34C, TP53

Selection of significant pathways specific to GNNenrich with $\beta = 10$ for the PD use case on Reactome

Pathway Name	BH	Size	Overlap	PPI	Gene annotated by Malacards
REMOVAL OF AMINOTERMINAL PROPEPTIDES FROM GAMMA CARBOXYLATED PROTEINS	2.85E-02	10	0	18	
GAMMA CARBOXYLATION TRANSPORT AND AMINO TERMINAL CLEAVAGE OF PROTEINS	3.26E-02	11	0	18	

Selection of significant pathways specific to GNNenrich with $\beta = 1$ for the GC use case on WikiPathways.

Pathway Name	BH	Size	Overlap	PPI	Gene annotated by Malacardsp
MAPK AND NFKB SIGNALING PATHWAYS INHIBITED BY YERSINIA YOPJ	2.82E-04	12	0	107	CHUK, MAPK1, NFKB1, NFKBIA, RAF1
4HYDROXYTAMOXIFEN DEXAMETHASONE AND RETINOIC ACIDS REGULATION OF P27 EXPRESSION	2.82E-04	18	0	176	AKT1, EIF4E, EIF4EBP1, MAP2K1, MAP3K11, MAPK1, MAPK3, MTOR, RAF1
OSTEOPONTIN SIGNALING	2.82E-04	13	0	128	CHUK, ITGAV, ITGB3, MAP2K1, MAPK1, MAPK3, MMP9, NFKB1, PLAU, RELA, SPP1
SARSCOV2 B117 VARIANT ANTAGONISES INNATE IMMUNE ACTIVATION	2.82E-04	9	0	35	~
ALTERED GLYCOSYLATION OF MUC1 IN TUMOR MICROENVIRONMENT	2.82E-04	9	0	73	CHUK, IL6, MUC1, NFKB1, NFKBIA, RELA, TNF
EPO RECEPTOR SIGNALING	2.82E-04	26	0	390	AKT1, GRB2, JAK2, MAP2K1, MAPK1, MAPK3, PIK3CG, PTPRC, RAF1, SOCS1, SRC, STAT1, STAT3, STAT5A
NODLIKE RECEPTOR NLR SIGNALING PATHWAY	2.82E-04	9	0	73	CD40, CHUK, EPHB2, MAPK8, RELA

Selection of significant pathways specific to GNNenrich with $\beta = 10$ for the GC use case on WikiPathways.

Pathway Name	BH	Size	Overlap	PPI	Gene annotated by Malacards
NODLIKE RECEPTOR NLR SIGNALING PATHWAY	5.94E-04	9	0	73	CD40, CHUK, EPHB2, MAPK8, RELA
MAPK AND NFKB SIGNALING PATHWAYS INHIBITED BY YERSINIA YOPJ	6.75E-03	12	0	107	CHUK, MAPK1, NFKB1, NFKBIA, RAF1
4HYDROXYTAMOXIFEN DEXAMETHASONE AND RETINOIC ACIDS REGULATION OF P27 EXPRESSION	2.17E-02	18	0	176	AKT1, EIF4E, EIF4EBP1, MAP2K1, MAP3K11, MAPK1, MAPK3, MTOR, RAF1
OSTEOPONTIN SIGNALING	2.66E-02	13	0	128	CHUK, ITGAV, ITGB3, MAP2K1, MAPK1, MAPK3, MMP9, NFKB1, PLAU, RELA, SPP1
NANOPARTICLE TRIGGERED REGULATED NECROSIS	2.97E-02	12	0	55	CASP8, FADD, MAPK8, PARP1, TNF
ALTERED GLYCOSYLATION OF MUC1 IN TUMOR MICROENVIRONMENT	3.01E-02	9	0	73	CHUK, IL6, MUC1, NFKB1, NFKBIA, RELA, TNF
EPO RECEPTOR SIGNALING	4.31E-02	26	0	390	AKT1, GRB2, JAK2, MAP2K1, MAPK1, MAPK3, PIK3CG, PTPRC, RAF1, SOCS1, SRC, STAT1, STAT3, STAT5A
PROSURVIVAL SIGNALING OF NEUROPROTECTIN D1	5.00E-02	15	0	81	BAX, BIRC3, CASP3, CASP8, CASP9, CYCS, FADD, TNF, TRAF2
SARSCOV2 B117 VARIANT ANTAGONISES INNATE IMMUNE ACTIVATION	6.52E-03	9	0	35	~

Selection of significant pathways specific to GNNenrich with $\beta = 1$ for the GC use case on Reactome.

Pathway Name	BH	Size	Overlap	PPI	Gene annotated by Malacards
MAP3K8 TPL2 DEPENDENT MAPK1 3 ACTIVATION	3.45E-04	16	0	152	BTRC, CHUK, MAP2K1, MAP2K4, NFKB1
IRAK2 MEDIATED ACTIVATION OF TAK1 COMPLEX	3.45E-04	10	0	125	
CHK1 CHK2 CDS1 MEDIATED INACTIVATION OF CYCLIN B CDK1 COMPLEX	3.45E-04	13	0	131	CCNB1, CDK1, CHEK1, CHEK2, YWHAE, YWHAZ
FLT3 SIGNALING THROUGH SRC FAMILY KINASES	3.45E-04	6	0	82	SYK

PTK6 REGULATES CELL CYCLE	3.45E-04	6	0	53	CCND1, CCNE1, CDK2, CDK4, CDKN1B
ACTIVATION OF BAD AND TRANSLOCATION TO MITOCHONDRIA	3.45E-04	15	0	140	AKT1, BAD, BCL2, BID, YWHAE, YWHAZ
P130CAS LINKAGE TO MAPK SIGNALING FOR INTEGRINS	3.45E-04	15	0	168	FN1, ITGB3, PTK2, SRC
ACTIVATED NTRK2 SIGNALS THROUGH FYN	3.45E-04	7	0	120	RAC1, SRC
TRIF MEDIATED PROGRAMMED CELL DEATH	3.45E-04	9	0	31	CASP8, CD14, FADD, TLR4
FCERI MEDIATED NF KB ACTIVATION	6.26E-04	136	0	497	BTRC, CHUK, NFKB1, NFKBIA, PDPK1, PSMB8, RELA
GRB2 SOS PROVIDES LINKAGE TO MAPK SIGNALING FOR INTEGRINS	6.26E-04	15	0	183	FN1, GRB2, ITGB3, PTK2, SRC
PROLACTIN RECEPTOR SIGNALING	8.46E-04	15	0	111	BTRC, JAK2, PTPN11, RBX1, STAT5A

Selection of significant pathways specific to GNNenrich with $\beta = 10$ for the GC use case on Reactome.

Pathway Name	BH	Size	Overlap	PPI	Gene annotated by Malacards
DRUG MEDIATED INHIBITION OF CDK4 CDK6 ACTIVITY	5.37E-03	5	0	40	CCND1, CCND2, CDK4, CDK6
PTK6 REGULATES CELL CYCLE	8.16E-03	6	0	53	CCND1, CCNE1, CDK2, CDK4, CDKN1B
ERYTHROPOIETIN ACTIVATES STAT5	1.79E-02	7	0	88	IRS2,JAK2,STAT5A
INTERLEUKIN 23 SIGNALING	1.87E-02	9	0	74	JAK2, P4HB, STAT3
ERYTHROPOIETIN ACTIVATES PHOSPHOLIPASE C GAMMA PLCG	2.43E-02	7	0	97	JAK2, PLCG2
G2 PHASE	2.54E-02	5	0	29	CCNA1, CCNA2, CDK2, E2F1
IRAK2 MEDIATED ACTIVATION OF TAK1 COMPLEX	2.90E-02	10	0	125	
TRIF MEDIATED PROGRAMMED CELL DEATH	3.71E-02	9	0	31	CASP8, CD14, FADD, TLR4
TNF RECEPTOR SUPERFAMILY TNFSF MEMBERS MEDIATING NON CANONICAL NF KB PATHWAY	3.83E-02	17	0	36	BIRC2, BIRC3, CD40, CD40LG, LTA, TNFRSF12A, TRAF2
AKT PHOSPHORYLATES TARGETS IN THE CYTOSOL	4.67E-02	14	0	100	AKT1, BAD, CASP9, CDKN1A, CDKN1B, CHUK, GSK3B, MDM2

Table 50. List of genes from pathways identified by GNNenrich ($\beta = 10$), with no overlap with the MalaCards Parkinson's disease gene set.

Gene	References
BGLAP	[Cao et al. (2022)]
F2,F7,F9,F10	[Rurak (2019)]
FURIN	[Zhang et al. (2022)]
GAS6	[Nunez Santos (2022)]
GGCX	[Ferland (2012)]
PROC	[Vayyat et al. (2025)]
PROS1	[Xing et al. (2022)]
PROZ	[Ferland (2013)]

24. Selection of the 10 top and bottom lines of the heatmaps

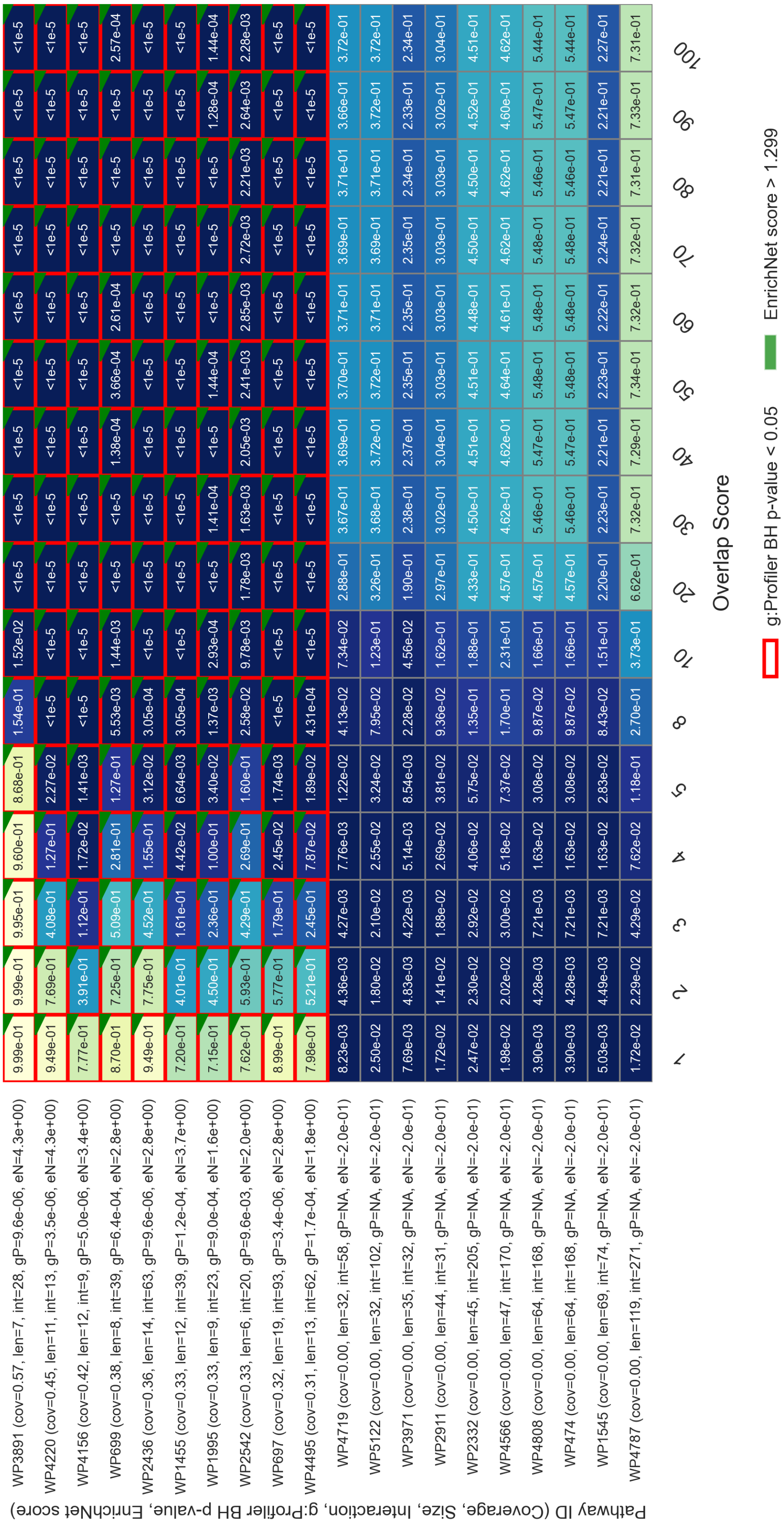

Fig. 27: Selection of the 10 top and bottom lines of the heatmap for **Parkinson**, illustrating pathway evolution detected in WikiPathways with their BH-adjusted p-values for $\beta \in [1, 100]$. Each cell shows GNNenrich adjusted p-values; red borders mark significant g:Profiler pathways, green corners mark EnrichNet detections.

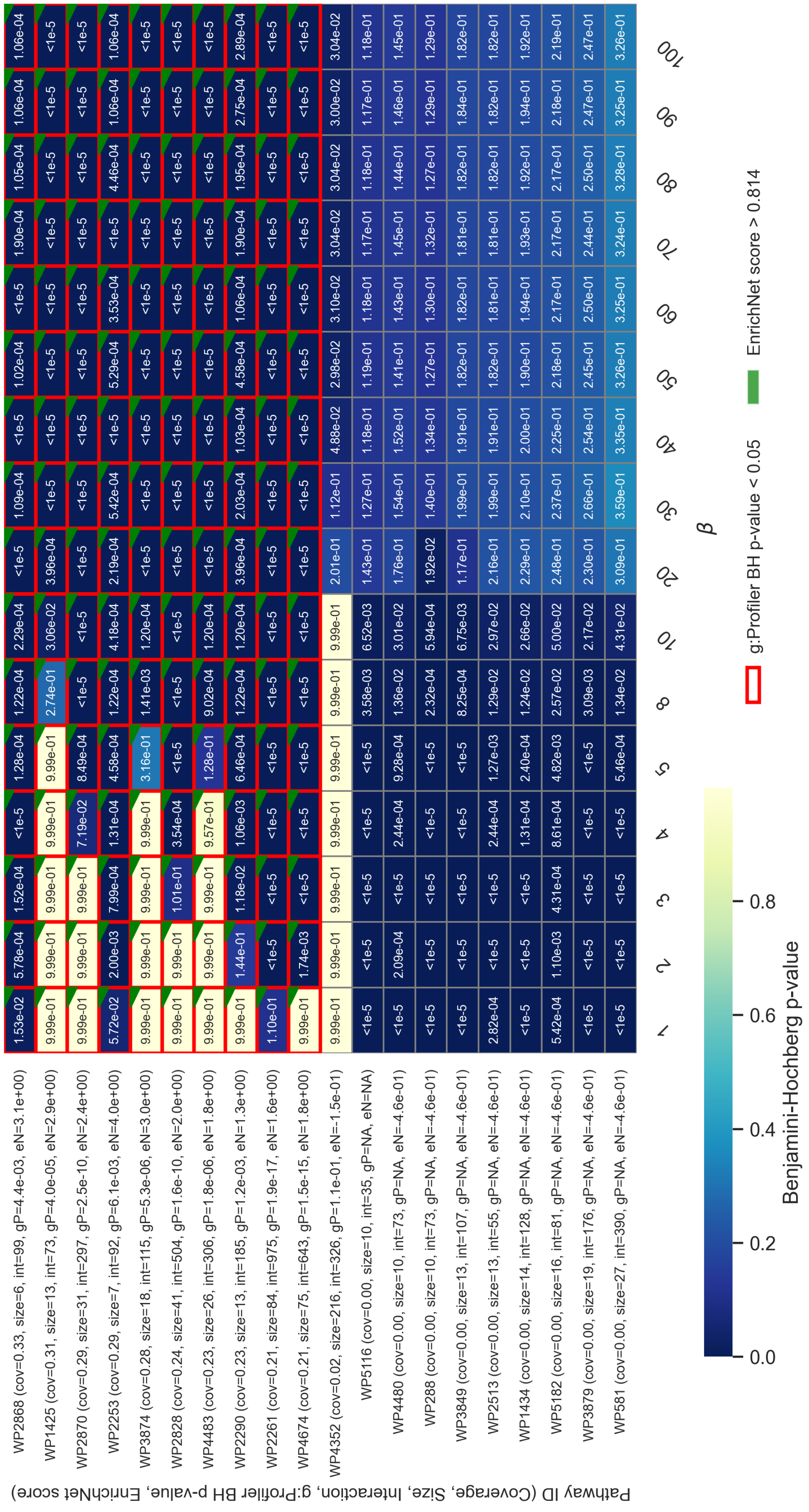

Fig. 28: Selection of the 10 top and bottom lines of the heatmap for **Gastric cancer**, same interpretation as Figure 27.

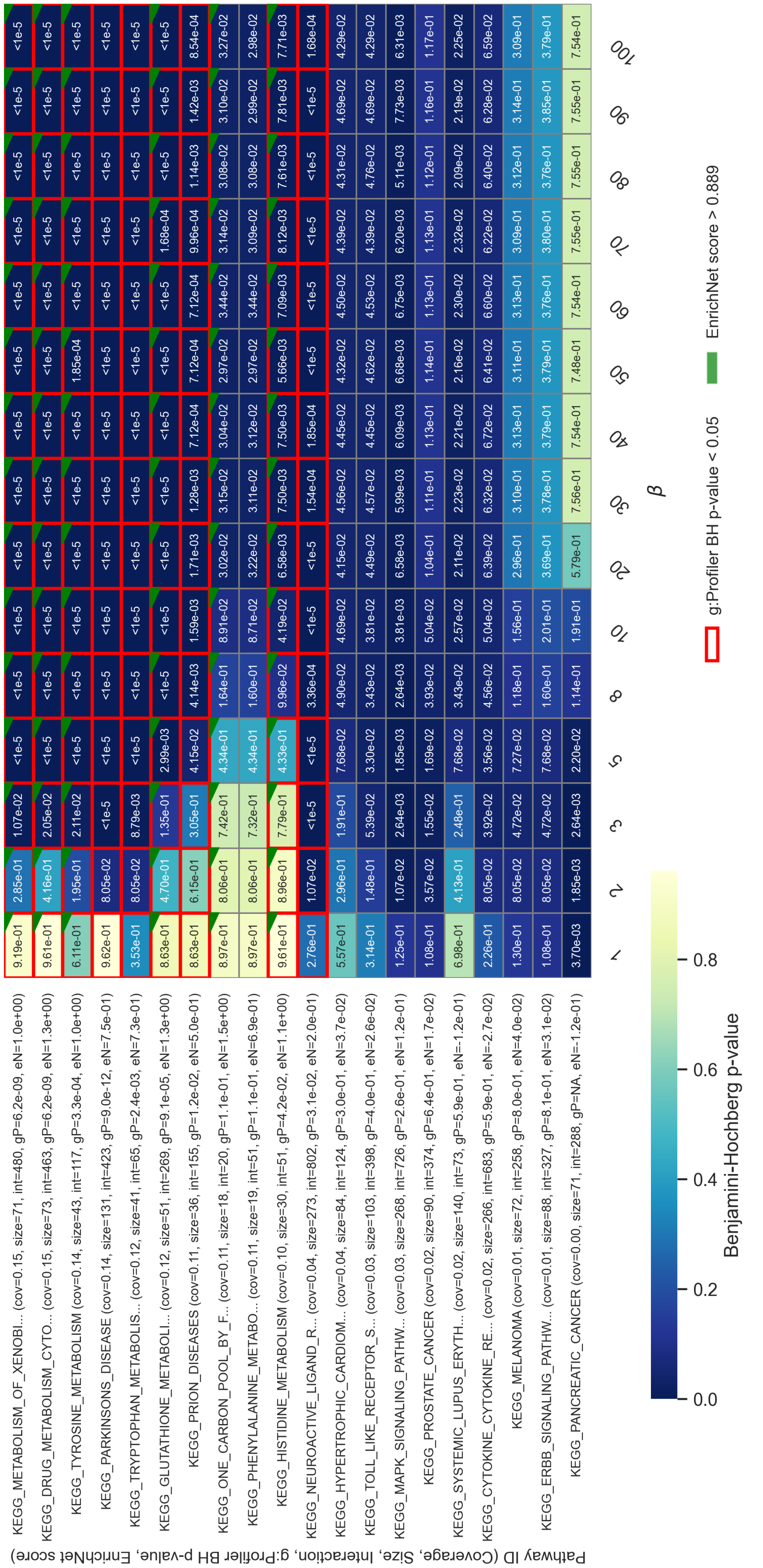

Fig. 29: Selection of the 10 top and bottom lines of the heatmap for **Parkinson** (KEGG database), illustrating pathway evolution and BH-adjusted p-values for $\beta \in [1, 100]$. Each cell shows GNNenrich adjusted p-values; red borders mark significant g:Profiler pathways, green corners indicate EnrichNet detections.

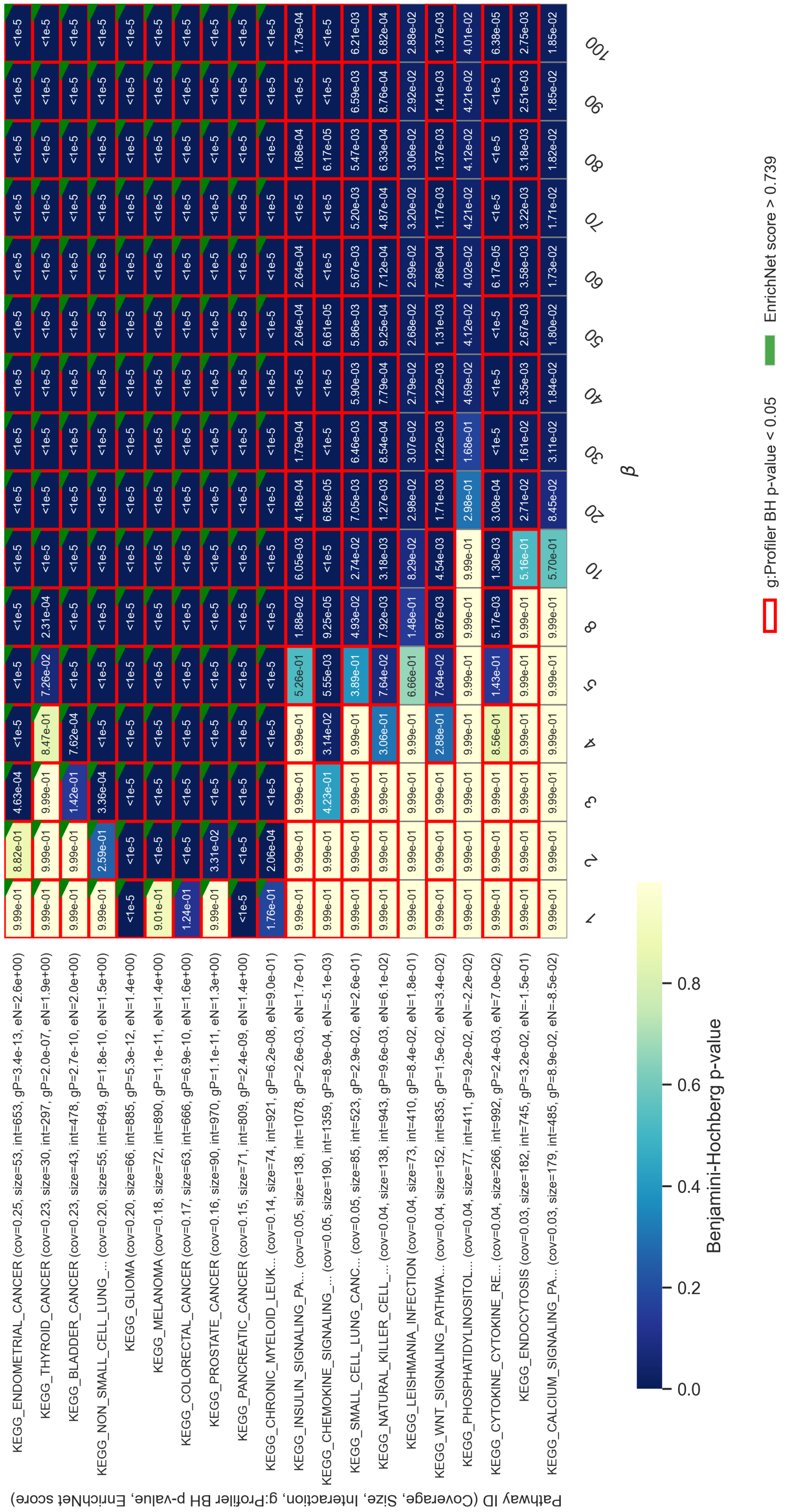

Fig. 30: {Selection of the 10 top and bottom lines of the heatmap for **Gastric cancer** (KEGG database), same interpretation as Figure 29.

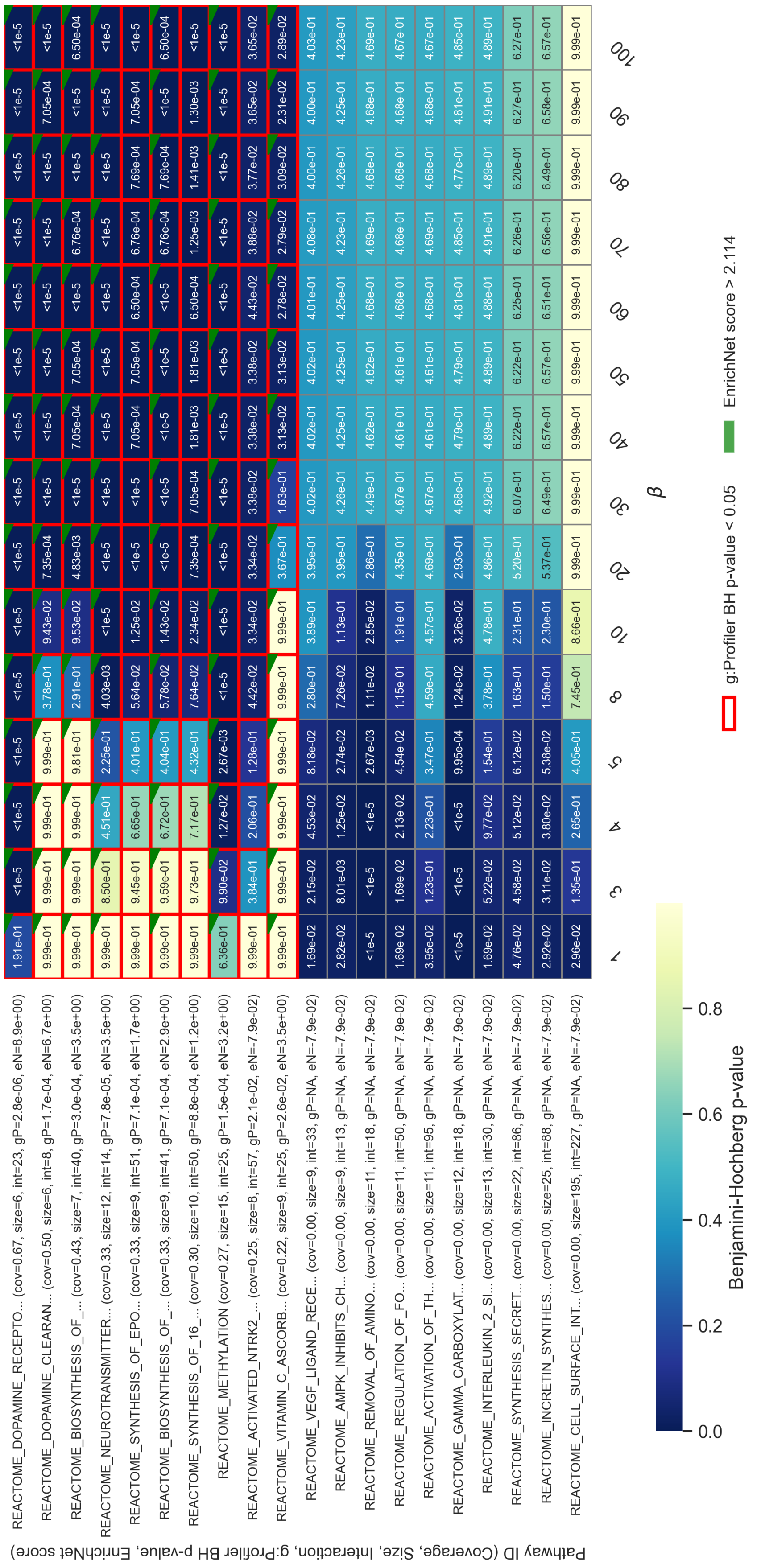

Fig. 31: Selection of the 10 top and bottom lines of the heatmap for **Parkinson** (Reactome database), illustrating pathway evolution and BH-adjusted p-values for $\beta \in [1, 100]$. Each cell shows GNNenrich adjusted p-values; red borders mark significant g:Profiler pathways, green corners indicate EnrichNet detections.

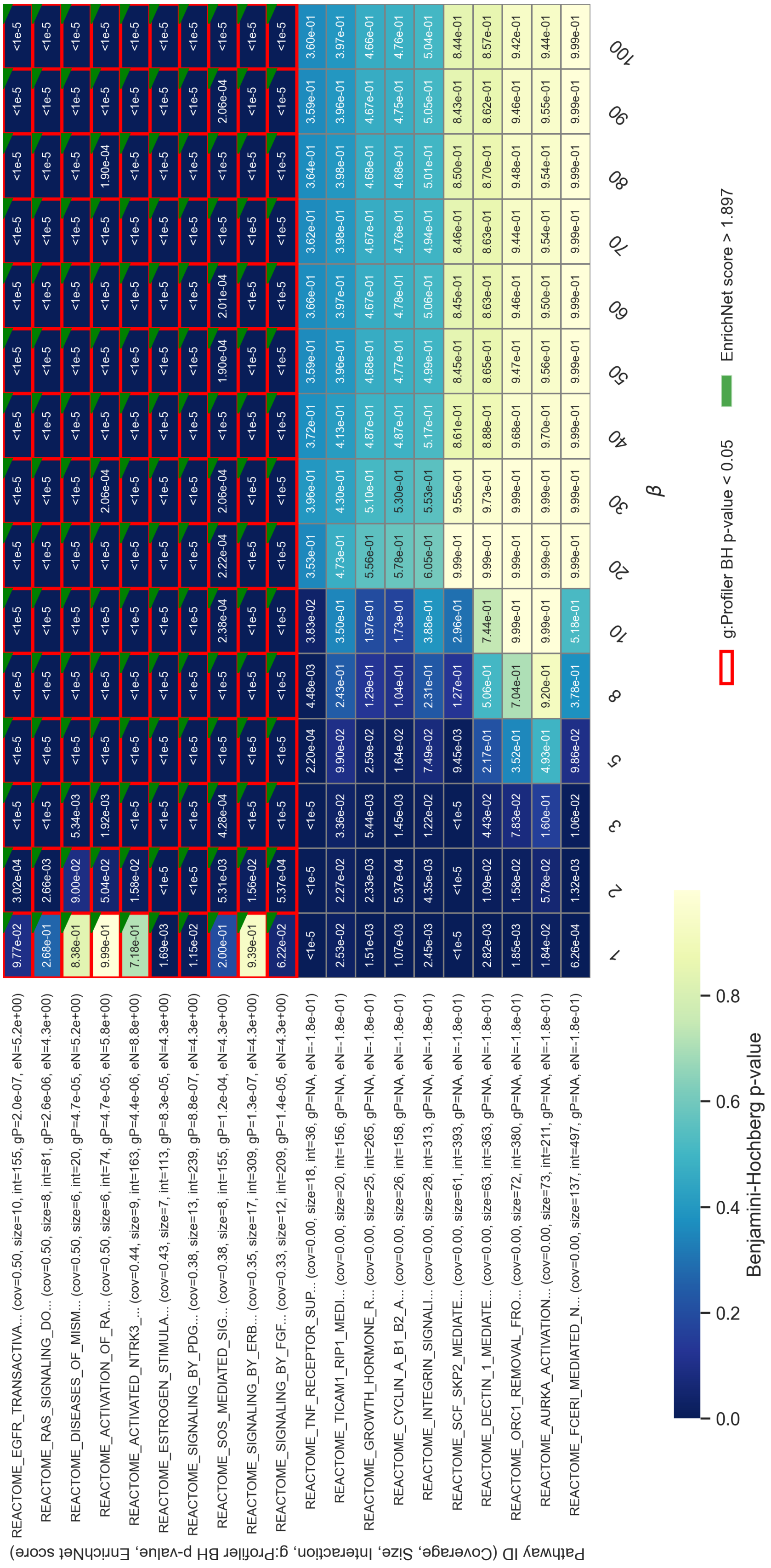

Fig. 32: Selection of the 10 top and bottom lines of the heatmap for **Gastric cancer** (Reactome database), same interpretation as Figure 31.

References

E. H. Ahn, S. S. Kang, and K. Ye. Netrin-1/receptors regulate the pathogenesis in parkinson's diseases. *Precision and Future Medicine*, 5(2):50–61, 2021.

T. Ashizawa, R. Okada, Y. Suzuki, M. Takagi, T. Yamazaki, T. Sumi, T. Aoki, S. Ohnuma, and T. Aoki. Clinical significance of interleukin-6 (il-6) in the spread of gastric cancer: role of il-6 as a prognostic factor. *Gastric Cancer*, 8:124–131, 2005.

S. Bamford, E. Dawson, S. Forbes, J. Clements, R. Pettett, A. Dogan, A. Flanagan, J. Teague, P. A. Futreal, M. R. Stratton, et al. The cosmic (catalogue of somatic mutations in cancer) database and website. *British journal of cancer*, 91(2):355–358, 2004.

M.-A. Bonte, F. El Idrissi, B. Gressier, D. Devos, and K. Belarbi. Protein network exploration prioritizes targets for modulating neuroinflammation in parkinson's disease. *International Immunopharmacology*, 95:107526, 2021.

E. L. Brown, H. T. Essigmann, K. L. Hoffman, A. S. Alexander, M. Newmark, Z.-D. Jiang, J. Suescun, M. C. Schiess, C. L. Hanis, and H. L. DuPont. Iga-biome profiles correlate with clinical parkinson's disease subtypes. *Journal of Parkinson's Disease*, 13(4):501–513, 2023.

M. Cao, J. Xie, Y. Hu, X. Gao, and Z. Xie. Dynamic molecular profiles of bone marrow-derived osteoblasts at the single-cell level. *Folia Biologica (Praha)*, 68:97–104, 2022.

M. Chen, C. J.-T. Ju, G. Zhou, X. Chen, T. Zhang, K.-W. Chang, C. Zaniolo, and W. Wang. Multifaceted protein–protein interaction prediction based on siamese residual rcnn. *Bioinformatics*, 35(14):i305–i314, 2019.

F. Ciccarese, E. Zulato, and S. Indraccolo. Lkb1/ampk pathway and drug response in cancer: a therapeutic perspective. *Oxidative medicine and cellular longevity*, 2019(1):8730816, 2019.

T. S. de Carvalho. Calorie restriction or dietary restriction: How far they can protect the brain against neurodegenerative diseases? *Neural Regeneration Research*, 17(8):1640–1644, 2022.

J. G. de Oliveira and A. E. Silva. Polymorphisms of the tlr2 and tlr4 genes are associated with risk of gastric cancer in a brazilian population. *World journal of gastroenterology: WJG*, 18(11):1235, 2012.

E. A. M. Enciso, M. A. López-Lizarraga, and J. F. Plascencia-Félix. Pre-excited atrial fibrillation in a patient with hypertrophic cardiomyopathy and wolff parkinson white syndrome. *Journal of the American College of Cardiology*, 81(8_Supplement):2829–2829, 2023.

Y.-f. Fan, Y.-m. Wu, H. Liu, Y. Yu, Y.-y. Jiang, Y.-z. Xue, Z.-l. Liu, and M.-X. Wei. Tlr4 polymorphisms associated with developing gastric pre-cancer lesions in a chinese han population. *Human Immunology*, 75(2):176–181, 2014.

G. Ferland. Vitamin k, an emerging nutrient in brain function. *Biofactors*, 38(2):151–157, 2012.

G. Ferland. Vitamin k and brain function. In *Seminars in thrombosis and hemostasis*, volume 39, pages 849–855. Thieme Medical Publishers, 2013.

J. R. Gardner, M. J. Kusner, Z. E. Xu, K. Q. Weinberger, and J. P. Cunningham. Bayesian optimization with inequality constraints. In *Proceedings of the 31st International Conference on Machine Learning (ICML)*, pages 937–945, 2014.

A. Hamosh, A. F. Scott, J. Amberger, D. Valle, and V. A. McKusick. Online mendelian inheritance in man (omim). *Human mutation*, 15(1):57–61, 2000.

M. Joling, O. A. van den Heuvel, H. W. Berendse, J. Booij, and C. Vriend. Serotonin transporter binding and anxiety symptoms in parkinson's disease. *Journal of Neurology, Neurosurgery & Psychiatry*, 89(1):89–94, 2018.

I. Y. Kim, Y. Eun, J. Lee, K. Han, D. H. Kim, J.-H. Min, H.-S. Cha, E.-M. Koh, D. W. Shin, and H. Kim. Systemic lupus erythematosus is associated with increased risk of parkinson's disease. *Therapeutic Advances in Musculoskeletal Disease*, 15:1759720X231152653, 2023.

N. Kim and H. J. Lee. Target enzymes considered for the treatment of alzheimer's disease and parkinson's disease. *BioMed Research International*, 2020(1):2010728, 2020.

T. N. Kipf and M. Welling. Semi-supervised classification with graph convolutional networks. *arXiv preprint arXiv:1609.02907*, 2016.

K. Kunii, L. Davis, J. Gorenstein, H. Hatch, M. Yashiro, A. Di Bacco, C. Elbi, and B. Lutterbach. Fgfr2-amplified gastric cancer cell lines require fgfr2 and erbb3 signaling for growth and survival. *Cancer research*, 68(7):2340–2348, 2008.

J. Leibold, K. M. Tsanov, C. Amor, Y.-J. Ho, F. J. Sánchez-Rivera, J. Feucht, T. Baslan, H.-A. Chen, S. Tian, J. Simon, et al. Somatic mouse models of gastric cancer reveal genotype-specific features of metastatic disease. *Nature cancer*, 5(2):315–329, 2024.

C. Li, X. Li, S. Gao, and L. Ma. Microrna-133a inhibits proliferation of gastric cancer cells by downregulating erbb2 expression. *Oncology research*, 25(7):1169, 2017.

G. Lv, Z. Hu, Y. Bi, and S. Zhang. Learning unknown from correlations: Graph neural network for inter-novel-protein interaction prediction. In *Proceedings of the Thirtieth International Joint Conference on Artificial Intelligence (IJCAI-21)*, 2021.

N. Maswood, J. Young, E. Tilmont, Z. Zhang, D. M. Gash, G. A. Gerhardt, R. Grondin, G. S. Roth, J. Mattison, M. A. Lane, et al. Caloric restriction increases neurotrophic factor levels and attenuates neurochemical and behavioral deficits in a primate model of parkinson's disease. *Proceedings of the National Academy of Sciences*, 101(52):18171–18176, 2004.

T. Mikolov, I. Sutskever, K. Chen, G. S. Corrado, and J. Dean. Distributed representations of words and phrases and their compositionality. *Advances in neural information processing systems*, 26, 2013.

J. A. Morales-Garcia, M. Redondo, S. Alonso-Gil, C. Gil, C. Perez, A. Martinez, A. Santos, and A. Perez-Castillo. Phosphodiesterase 7 inhibition preserves dopaminergic neurons in cellular and rodent models of parkinson disease. *PLOS One*, 6(2):e17240, 2011.

M. G. Murer, Q. Yan, and R. Raisman-Vozari. Brain-derived neurotrophic factor in the control human brain, and in alzheimer's disease and parkinson's disease. *Progress in Neurobiology*, 63(1):71–124, 2001.

R. T. Murphy, J. Mogensen, K. McGarry, A. Bahl, A. Evans, E. Osman, P. Syrris, G. Gorman, M. Farrell, J. L. Holton, et al. Adenosine monophosphate-activated protein kinase disease mimicks hypertrophic cardiomyopathy and wolff-parkinson-white syndrome: natural history. *Journal of the American College of Cardiology*, 45(6):922–930, 2005.

I. Nandi and B. Aroeti. Mitogen-activated protein kinases (mapks) and enteric bacterial pathogens: a complex interplay. *International Journal of Molecular Sciences*, 24(15):11905, 2023.

F. Niccolini, H. Wilson, G. Pagano, C. Coello, M. A. Mehta, G. E. Searle, R. N. Gunn, E. A. Rabiner, T. Foltynie, and M. Politis. Loss of phosphodiesterase 4 in parkinson disease: Relevance to cognitive deficits. *Neurology*, 89(6):586–593, 2017.

C. Niu, M. Dong, and Y. Niu. Role of glutathione in parkinson's disease pathophysiology and therapeutic potential of polyphenols. *Phytotherapy Research*, 2024.

C. Noelker, L. Morel, T. Lescot, A. Osterloh, D. Alvarez-Fischer, M. Breloer, C. Henze, C. Depboylu, D. Skrzydelski, P. P. Michel, et al. Toll like receptor 4 mediates cell death in a mouse mptp model of parkinson disease. *Scientific reports*, 3(1):1393, 2013.

M. Nunez Santos. Interactions between progranulin, cd68, and gas6 and implications for inflammation and neurodegeneration. 2022.

C. Pellegrini, L. Antonioli, R. Colucci, C. Blandizzi, and M. Fornai. Interplay among gut microbiota, intestinal mucosal barrier and enteric neuro-immune system: a common path to neurodegenerative diseases? *Acta neuropathologica*, 136:345–361, 2018.

Y. Peng, Y. Xu, X. Zhang, S. Deng, Y. Yuan, X. Luo, M. T. Hossain, X. Zhu, K. Du, F. Hu, et al. A novel protein axin1-295aa encoded by circaxin1 activates the wnt/β-catenin signaling pathway to promote gastric cancer progression. *Molecular cancer*, 20:1–19, 2021.

B. Picconi, G. Piccoli, and P. Calabresi. Synaptic dysfunction in parkinson's disease. *Synaptic Plasticity: Dynamics, Development and Disease*, pages 553–572, 2012.

P. Riederer, J. Bartl, G. Laux, and E. Grünblatt. Diabetes type ii: A risk factor for depression–parkinson–alzheimer? *Neurotoxicity research*, 19:253–265, 2011.

G. M. e. a. Rurak. Translatomic database of cortical astroglia across male and female mouse development reveals two distinct developmental phenotypes. *bioRxiv*, page 681684, 2019.

T. Schirinzi, G. Madeo, G. Martella, M. Maltese, B. Picconi, P. Calabresi, and A. Pisani. Early synaptic dysfunction in parkinson's disease: Insights from animal models. *Movement Disorders*, 31(6):802–813, 2016.

M. Sereno, J. De Castro, C. Belda-Iniesta, M. A. Garcia-Cabezas, P. Cejas, E. Casado, J. Barriuso, J. Feliu, and J. Larrauri. Epo-r expression patterns in resected gastric adenocarcinoma followed by adjuvant chemoradiation treatment. *Pathology & Oncology Research*, 15:1–10, 2009.

J. Shen, J. Zhang, X. Luo, W. Zhu, K. Yu, K. Chen, Y. Li, and H. Jiang. Predicting protein–protein interactions based only on sequences information. *Proceedings of the National Academy of Sciences*, 104(11):4337–4341, 2007.

M. Smeyne and R. J. Smeyne. Glutathione metabolism and parkinson's disease. *Free Radical Biology and Medicine*, 62:13–25, 2013.

S. Srivastava and M. C Haigis. Role of sirtuins and calorie restriction in neuroprotection: implications in alzheimer's and parkinson's diseases. *Current pharmaceutical design*, 17(31):3418–3433, 2011.

A. Tojjari, S. Nagdas, A. Saeed, and A. Saeed. Deciphering the fgfr2 code: innovative targets in gastric cancer therapy. *Current Oncology*, 31(8):4305–4317, 2024.

T. Trotta, C. Porro, R. Calvello, and M. A. Panaro. Biological role of toll-like receptor-4 in the brain. *Journal of neuroimmunology*, 268 (1-2):1–12, 2014.

S. Vayyat, S. C. Singh, and P. Mishra. Coagulation system and its neurophysiology. In *Brain and Organ Communication*, pages 215–227. Elsevier, 2025.

J. Wang, F. Wang, D. Mai, and S. Qu. Molecular mechanisms of glutamate toxicity in parkinson's disease. *Frontiers in neuroscience*, 14:585584, 2020.

M. Wang, Y. Zhao, B. Zhang, M. M. Wang, and C. M. Wang. Package 'superexacttest'. *Nature*, 502:333–339, 2013.

C. Wantaneeyawong, N. Kasitanon, K. Kumchana, and W. Louthrenoo. Acute parkinsonism in patients with systemic lupus erythematosus: a case report and review of the literature. *International Journal of Neuroscience*, 132(9):868–873, 2022.

J. Wu, B. Liu, J. Zhang, Z. Wang, and J. Li. Dl-ppi: a method on prediction of sequenced protein–protein interaction based on deep learning. *BMC bioinformatics*, 24(1):473, 2023.

N. Xing, Z. Dong, Q. Wu, P. Kan, Y. Han, X. Cheng, and B. Zhang. Identification and validation of key molecules associated with humoral immune modulation in parkinson's disease based on bioinformatics. *Frontiers in Immunology*, 13:948615, 2022.

S.-Y. Yang, M. Beavan, K.-Y. Chau, J.-W. Taanman, and A. H. Schapira. A human neural crest stem cell-derived dopaminergic neuronal model recapitulates biochemical abnormalities in gba1 mutation carriers. *Stem Cell Reports*, 8(3):728–742, 2017a.

Y.-W. Yang, T.-F. Hsieh, C.-I. Li, C.-S. Liu, W.-Y. Lin, J.-H. Chiang, T.-C. Li, and C.-C. Lin. Increased risk of parkinson disease with diabetes mellitus in a population-based study. *Medicine*, 96(3):e5921, 2017b.

W. Yu, M. Clyne, M. J. Khoury, and M. Gwinn. Phenopedia and genopedia: disease-centered and gene-centered views of the evolving knowledge of human genetic associations. *Bioinformatics*, 26(1):145–146, 2010.

T. Zhan, N. Rindtorff, and M. Boutros. Wnt signaling in cancer. *Oncogene*, 36(11):1461–1473, 2017.

Y. Zhang, X. Gao, X. Bai, S. Yao, Y.-Z. Chang, and G. Gao. The emerging role of furin in neurodegenerative and neuropsychiatric diseases. *Translational Neurodegeneration*, 11(1):39, 2022.

Y. Zhou, S. Yu, and W. Zhang. Nod-like receptor signaling pathway in gastrointestinal inflammatory diseases and cancers. *International Journal of Molecular Sciences*, 24(19):14511, 2023.
